# Landscape of genome-wide age-related DNA methylation in breast tissue

**DOI:** 10.18632/oncotarget.22754

**Published:** 2017-11-29

**Authors:** Min-Ae Song, Theodore M. Brasky, Daniel Y. Weng, Joseph P. McElroy, Catalin Marian, Michael J. Higgins, Christine Ambrosone, Scott L. Spear, Adana A. Llanos, Bhaskar V.S. Kallakury, Jo L. Freudenheim, Peter G. Shields

**Affiliations:** ^1^ Comprehensive Cancer Center, The Ohio State University and James Cancer Hospital, Columbus, OH, USA; ^2^ College of Public Health, The Ohio State University, Columbus, OH, USA; ^3^ Center for Biostatistics and Department of Bioinformatics, The Ohio State University, Columbus, OH, USA; ^4^ Biochemistry and Pharmacology Department, Victor Babes University of Medicine and Pharmacy, Timisoara, Romania; ^5^ Department of Molecular and Cellular Biology, Roswell Park Cancer Institute, Buffalo, NY, USA; ^6^ Department of Cancer Prevention and Control, Roswell Park Cancer Institute, Buffalo, NY, USA; ^7^ Department of Plastic Surgery, Georgetown University, Washington, DC, USA; ^8^ Department of Epidemiology, Rutgers School of Public Health and Rutgers Cancer Institute of New Jersey, New Brunswick, NJ, USA; ^9^ Department of Pathology, Georgetown University, Washington, DC, USA; ^10^ Department of Epidemiology and Environmental Health, School of Public Health and Health Professions, University at Buffalo, Buffalo, NY, USA

**Keywords:** genome-wide DNA methylation, age, breast, normal, cancer

## Abstract

Despite known age-related DNA methylation (aDNAm) changes in breast tumors, little is known about aDNAm in normal breast tissues. Breast tissues from a cross-sectional study of 121 cancer-free women, were assayed for genome-wide DNA methylation. mRNA expression was assayed by microarray technology. Analysis of covariance was used to identify aDNAm’s. Altered methylation was correlated with expression of the corresponding gene and with DNA methyltransferase protein DNMT3A, assayed by immunohistochemistry. Publically-available TCGA-BRCA data were used for replication. 1,214 aDNAm’s were identified; 97% with increased methylation, and all on autosomes. Sites with increased methylation were predominantly in CpG lslands and non-enhancers. aDNAm’s with decreased methylation were generally located in intergenic regions, non-CpG Islands, and enhancers. Of the aDNAm’s identified, 650 are known to be involved in cancer, including *ESR1* and beta-estradiol responsive genes. Expression of DNMT3A was positively associated with age. Two aDNAm’s showed borderline significant associations with DNMT3A expression; *KRR1* (OR 6.57, 95% CI: 2.51–17.23) and *DHRS12* (OR 6.08, 95% CI: 2.33–15.86). A subset of aDNAm’s co-localized within vulnerable regions for somatic mutations in cancers including breast cancer. Expression of C19orf48 was inversely and significantly correlated with its methylation level. In the TCGA dataset, 84% and 64% of the previously identified aDNAm’s were correlated with age in both normal-adjacent and tumor breast tissues, with differential associations by histological subtype. Given the similarity of findings in the breast tissues of healthy women and breast tumors, aDNAm’s may be one pathway for increased breast cancer risk with age.

## INTRODUCTION

It is well established that breast cancer incidence increases with age [1, 2]. Among U.S. women, approximately 12% of invasive breast cancers are diagnosed in women < 45 years of age while 68% are diagnosed among women who are over age 55 [1, 2]. The underlying mechanism for this large difference in risk is not well understood. One potential mechanism is epigenetic alterations, including DNA methylation, which is one of the hallmarks of aging [3] and breast carcinogenesis [4–7].

Increased age is associated with global hypomethylation of CpG loci outside of CpG Islands and also regional hypermethylation of CpG Islands [6–11]. Methylation of the tumor suppressor genes *ESR1*, *IGFBP3,* and *RASSF1A* specifically increases with age [6, 7, 10, 12], but the converse occurs with methylation of repetitive elements [11]. Little is known about the timing of altered DNA methylation or age-related DNA methylation (aDNAm) in breast carcinogenesis, and whether it is different from normal aging in normal breast epithelial cells. A few studies that consist of only small sample sizes (*n* = 23 and *n* = 15 in two separate analyses) have examined aDNAm in normal breast tissues from healthy individuals [7, 13]. A recently published study with 100 samples showed aDNAm at regulatory regions [14]. However, to date, there has been no study of the role of aDNAm with gene expression in normal breast tissues, although, there some contradictory evidence for this in blood cells [13, 15–18].

In this study, aDNAm was assessed in the breasts of women with no prior history of cancer, and the results were compared to aDNAm tumor tissues from The Cancer Genome Atlas (TCGA). Further, to better understand the mechanism of these changes and their impact on carcinogenesis, gene expression for these aDNAm were assessed, and if the aDNAm varied with DNA methyltransferase protein levels.

## RESULTS

### Characteristics of study subjects

Characteristics of study subjects are given in Table 1. Subjects’ ages ranged from 17 to 76 years, with a mean of 38 years. The women were 77% premenopausal and 67% were of European American ancestry. Consistent with patients who typically seek breast reduction surgery, many women were overweight or obese (mean BMI: 30 kg/m^2^; range 21–46).

**Table 1:** Characteristics of study participants

Characteristic	Study samples (*n* = 121)		
	No.	Mean (range) or %	
*Demographic and clinical characteristics*			
Age, years	121	38	(17–76)
Race			
European American	81		67%
African American	40		33%
BMI, kg/m^2^	121	30	(21–46)
Age at menarche	91	13	(9–16)
Missing	30	-	
Parity			
Nulliparous	53		55%
1	14		14%
2	20		21%
3+	10		10%
Missing	24	-	
Age at first birth, years	34	27	(17–38)
Missing	87	-	
Alcohol status^a^			
Ever drinker	99		88%
Never drinker	14		12%
Missing	8	-	
Smoking status^b^			
Ever smoker	33		34%
Never smoker	65		66%
Missing	23		
Menopausal status			
Pre-menopausal	91		77%
Post-menopausal	27		23%
Missing	3		
1st degree relatives with breast cancer			
No	81		91%
Yes	8		9%
Missing	32		

^a^ ≥12 [ever] and < 12 [never] beverages over the course of a lifetime

^b^Ever smoked >= 100 and Never < 100 cigarettes over the course of your lifetime.

### Landscape of age-related DNA methylation

Using a Bonferroni corrected *P* < 0.05, after adjusting for race and BMI, 1,214 aDNAm’s were identified. As shown in Supplementary Table 1, for most of the aDNAm’s there was a gain of methylation (97%, 1,179/1,214). The aDNAm’s were located in 803 unique coding genes (978 CpGs), 8 unique long-noncoding RNAs (lncRNAs) (11 CpGs), and 8 unique microRNAs (miRNAs; mir-7-3, mir-15b, mir-16-2, mir-148A, mir-425, mir-596, mir-935, and mir-1253) (15 CpGs). All the miRNA aDNAm’s were located in core promoter regions, TSS1500 or TSS200.

**Figure 1:**
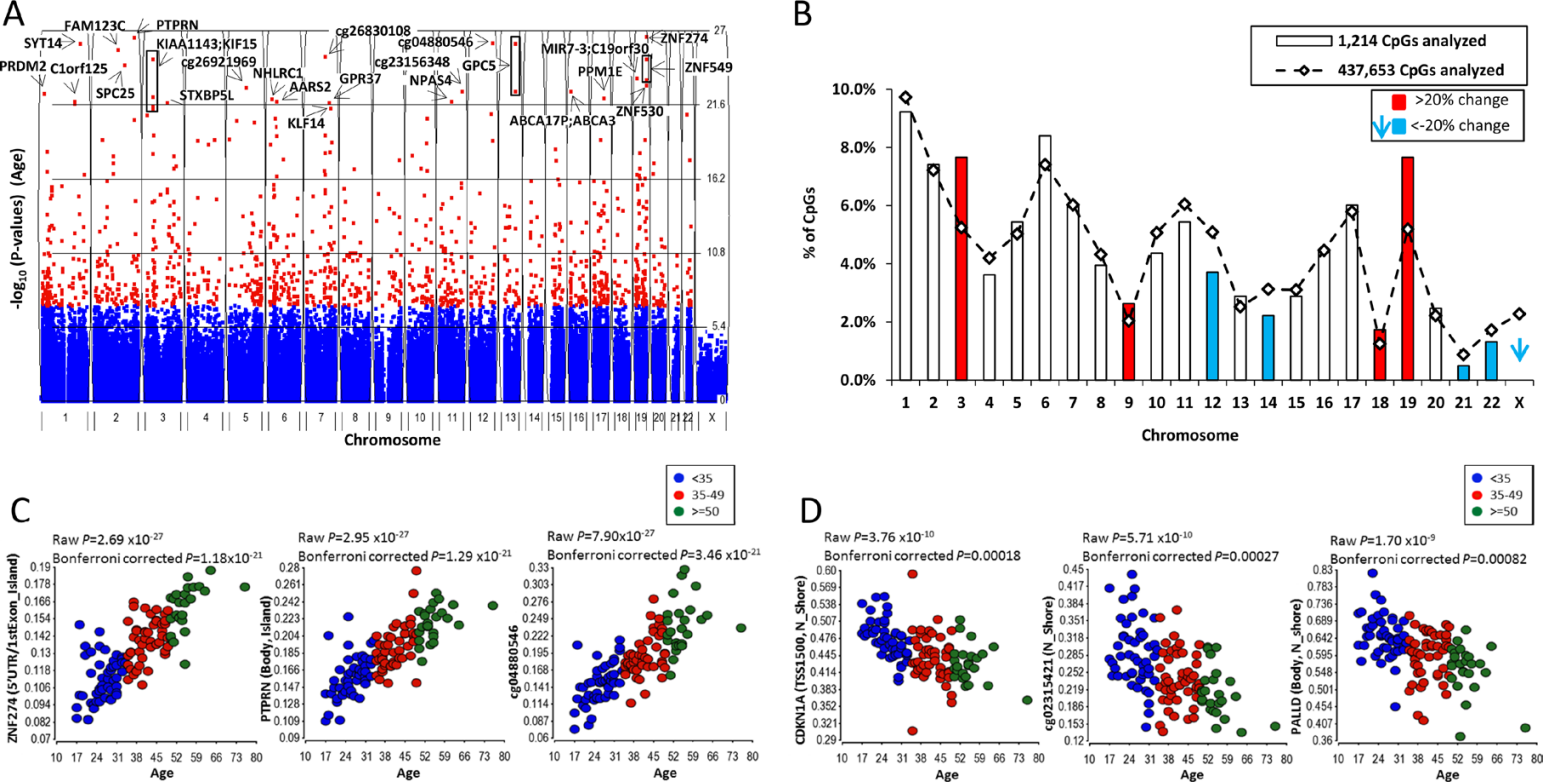
Age-related DNA methylation (**A**) Manhattan plot showing aDNAm’s identified in normal breast tissues (*n* = 121). The significance −log_10_ (*P*-value) of the associations of DNA methylation with aging by chromosomes is shown. Each dot indicates each CpG. Red and blue dots reporesent Bonferroni corrected *P*-value < 0.05 and > 0.05, respectively. The top 30 aDNAm’s are indicated with an arrow and gene name. If there were no corresponding gene to aDNAm’s, a target ID (Illumina array) is shown. A box shows multiple aDNAm’s corresponding to one gene. (**B**) Comparison of the proportion of expected (line) and observed (box) aDNAm’s by chromosome. An expected % was calculated based on a total 437,653 CpGs analyzed on the array and the observed % was calculated among 1,214 aDNAm’s at Bonferroni corrected *P* < 0.05. Chromosomes in red and blue are those substantialy (20%) higher or lower than expected, respectively. Dot plots of beta-values for top 3 aDNAm’s for gain (**C**) and loss (**D**) of methylation based on the *p*-value. Each point represents the beta-value for an individual (*n* = 121). Three age groups are colored for < 35 (blue), 35–49 (red), and > = 50 (green) for visualization. The raw and bonferroni corrected *P*-value for the association of DNA methylation with age are shown.

A Manhattan plot of the aDNAm’s is shown in Figure 1. The aDNAm’s are spread across all of the autosomes; although 2.3% of aDNAm’s would be expected to be on the X chromosome by chance, none were found. The top 30 aDNAm’s are listed. A substantially higher frequency of aDNAm’s were found on chromosomes 3, 9, 18, and 19 (> 20%, indicated in red), and a substantially lower frequency on chromosomes 12, 14, 21, and 22 (< −20%, blue) than expected given the proportion of CpGs on the array (Figure 1B).

Overall, the correlations for those aDNAm’s with increased methylation were stronger (partial correlations 0.41 to 0.78) than for those with decreased methylation (partial correlations −0.53 to −0.45) (Supplementary Table 1). aDNAm’s most strongly associated with age, with increased and decreased methylation are shown as dot plots in Figures 1C and 1D. The three aDNAm’s with the strongest statistical association with age were localized in the 5′UTR or 1stExon of *ZNF274*, the gene body of *PTPRN*, and an intergenic CpG locus (cg04880546) (Figure 1C). Those which decreased mostly strongly in association with age were localized in the TSS1500 of *CDKN1A*, the body of *PALLD*, and one intergenic CpG (cg02315421) (Figure 1D).

### Genomic features of aDNAm’s

**Figure 2:**
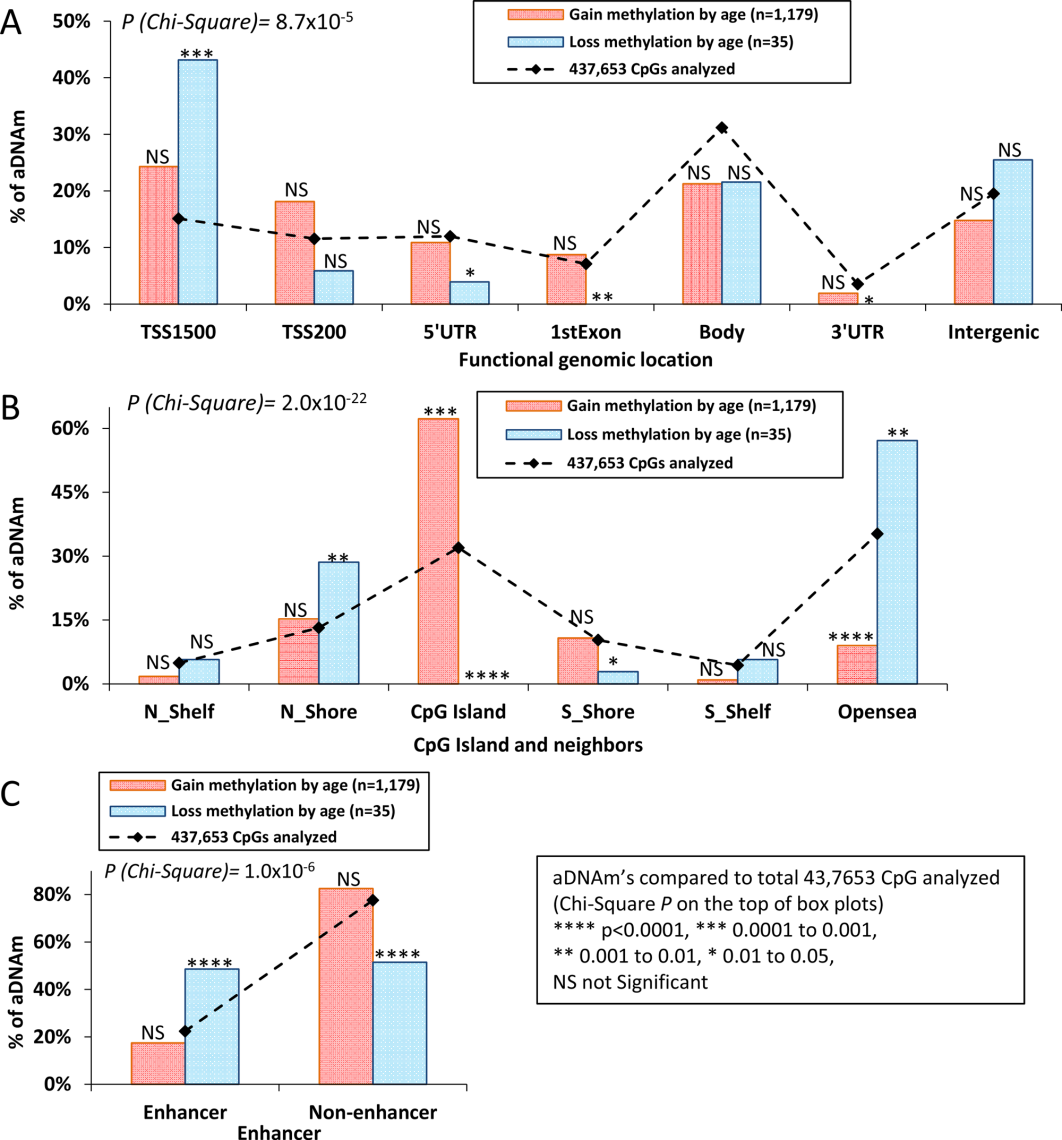
Box plots of proportion of aDNAm’s Box plots showed the expected (line) and observed (box) aDNAm’s by functional genomic location (**A**) CpGIslands/shores/shelf/opensea (**B**) and enhancer (**C**) Pink and blue boxes represent gain and loss of methylation, respectively. A proportion was tested (Chi-Squre) for a difference across regions between gain and loss of methylation (*P*-value in the box) and for a difference of gain/loss methylation compared to the expected aDNAm’s (*P-* value on the topt of box plot).

Of the 1,214 aDNAm’s identified, 73% and 60% were located in annotated promoters and CpG Islands, respectively. The distribution of aDNAm’s between gain and loss of methylation was significantly different across functionally annotated genomic locations and CpG Islands/shores/shelf at *P* = 8.7 × 10^−5^ and *P* = 2.0×10^−22^, respectively (Figure 2A–2B). Compared to the total number of CpGs analyzed, overall, aDNAm’s with increased methylation (*n* = 1,179) were enriched in core promoter regions [TSS1500 (24%) and TSS200 (18%)] (Figure 2A) and CpG Islands (Figure 2B), while aDNAm’s with decreased methylation (*n* = 35) were enriched in TSS1500 (43%), intergenic (25%) (Figure 2A) and open sea (57%) regions (Figure 2B). About half of the aDNAm’s (49%) with decreased methylation were located in enhancer regions, while a majority of aDNAm (83%) with increased methylation was located in non-enhancers (*P* = 1.6 × 10^−6^) (Figure 2C).

The overlapping genomic location of aDNAm’s with sites prone to mutation in human cancer was found using the COSMIC mutation database. Fifteen aDNAm’s were co-localized within vulnerable genomic regions where somatic mutations occur, including for breast cancer (*NEFM*) (Supplementary Table 2). Three of these loci were in genes for transcriptional regulators (*PAX5*, *SOX21*, and *ZGPAT*). All overlapped aDNAm’s were located in CpG Islands (*n* = 10) or shores/shelf (*n* = 4) except one locus (Supplementary Table 2).

### Potential biological implications of identified aDNAm’s

**Figure 3:**
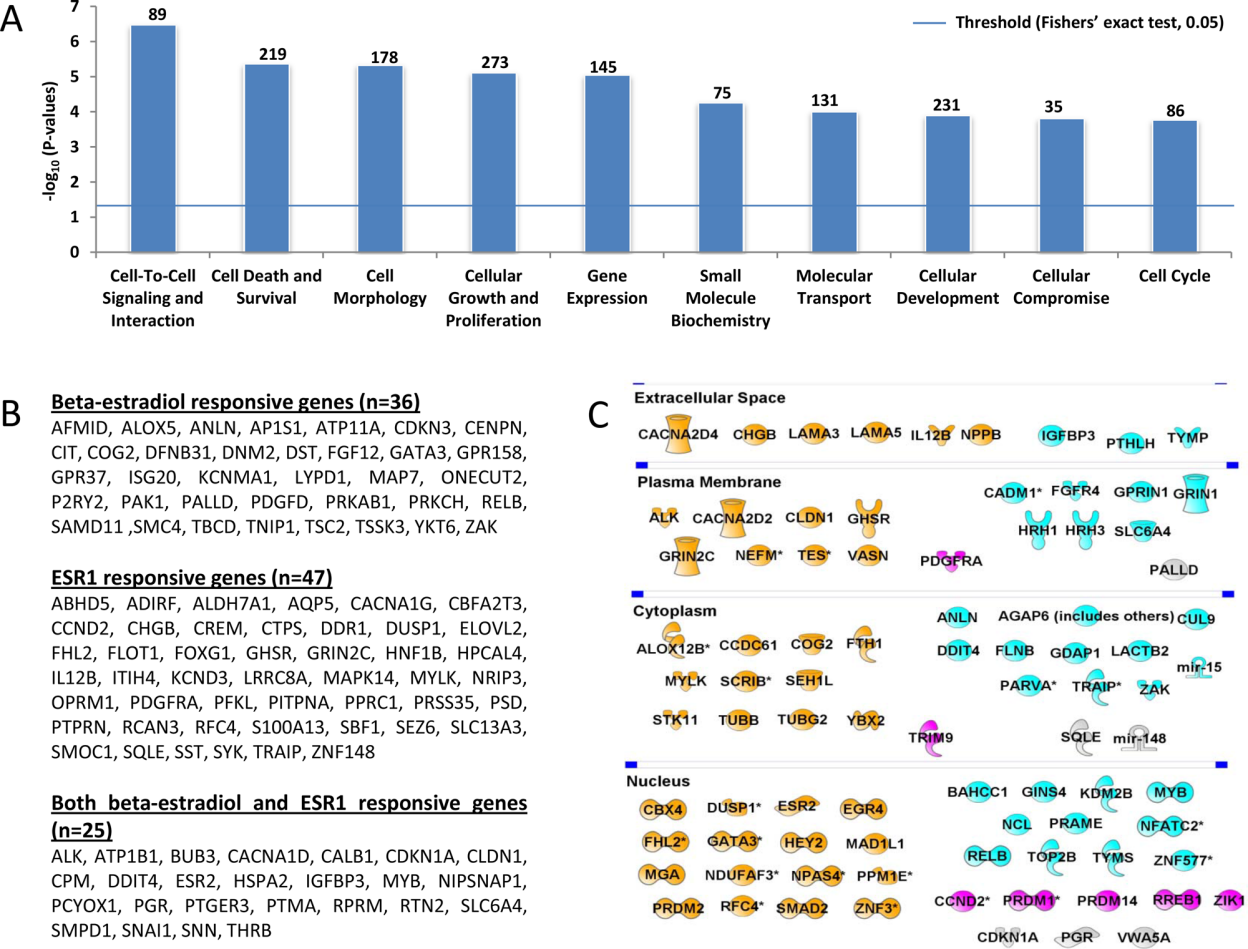
The most important molecular and cellular functions of the 1,214 aDNAm’s, beta-estradiol or/and ESR1 responsive, and breast cancer related genes identified by IPA (**A**) IPA categorized genes corresponding to aDNAm’s by molecular and cellular function. The number above a box plot indicates the number of aDNAm’s in a functional group. The horizontal line represents threshold *p*-value of 0.05 (Fishers’ exact test). (**B**) Lists of beta-estradiol or/and *ESR1* responsive genes corresponding to aDNAm’s are shown. (**C**) Among genes corresponding to aDNAm’s, 86 breast cancer related genes are shown by spatial location of molecules. Genes confirmed to be associated with age are colored. Orange colored genes was confirmed to be associated with age in both breast adjacent normal and tumor tissues from TCGA independent datasets. Light blue or pink colored genes were associated with age in either adjacent normal or tumor, respectively. Gray colored genes were not validated.

Among the 829 unique genes containing aDNAm’s, 784 were included in the IPA dataset. Molecular and cellular functions of genes containing aDNAm’s were significantly enriched for cell-to-cell signaling and interactions (*n* = 89), cell death and survival (*n* = 219), cell morphology (*n* = 178), cellular growth and proliferation (*n* = 273), and gene expression (*n* = 145) (Figure 3A). IPA’s upstream analysis showed enrichment for genes for *ESR1* (*n* = 72) and beta-estradiol responsive genes (*n* = 61) (Figure 3B). Of these, 25 genes were responsive to both *ESR1* and beta-estradiol responsive genes.

Among the 784 genes available in the IPA dataset, 650 (83%) were involved in cancer of some type. About 13% of these cancer-related genes (*n* = 86) are known to play a role in breast cancer (Figure 3C). Of the breast cancer related genes, thirty-five (41%) are nuclear proteins and 15 (17%) are transcription regulators [*CBX4, EGR4, FHL2, GATA3, HEY2, MGA, MYB, NFATC2, NPAS4, PRDM1, PRDM2, RELB, RREB1, SMAD2,* and *ZNF3* (Figure 3C)]. Among breast cancer-related genes, 50% were also associated with age in both breast tumor tissues and adjacent normal tissues in the TCGA-BRCA dataset (orange in Figure 3C). An additional 30 (35%) and 7 (8%) were associated with age in the adjacent normal (light blue in Figure 3C) or tumor (pink in Figure 3C), respectively.

### Association of DNMT3A protein expression with age and aDNAm’s

**Figure 4:**
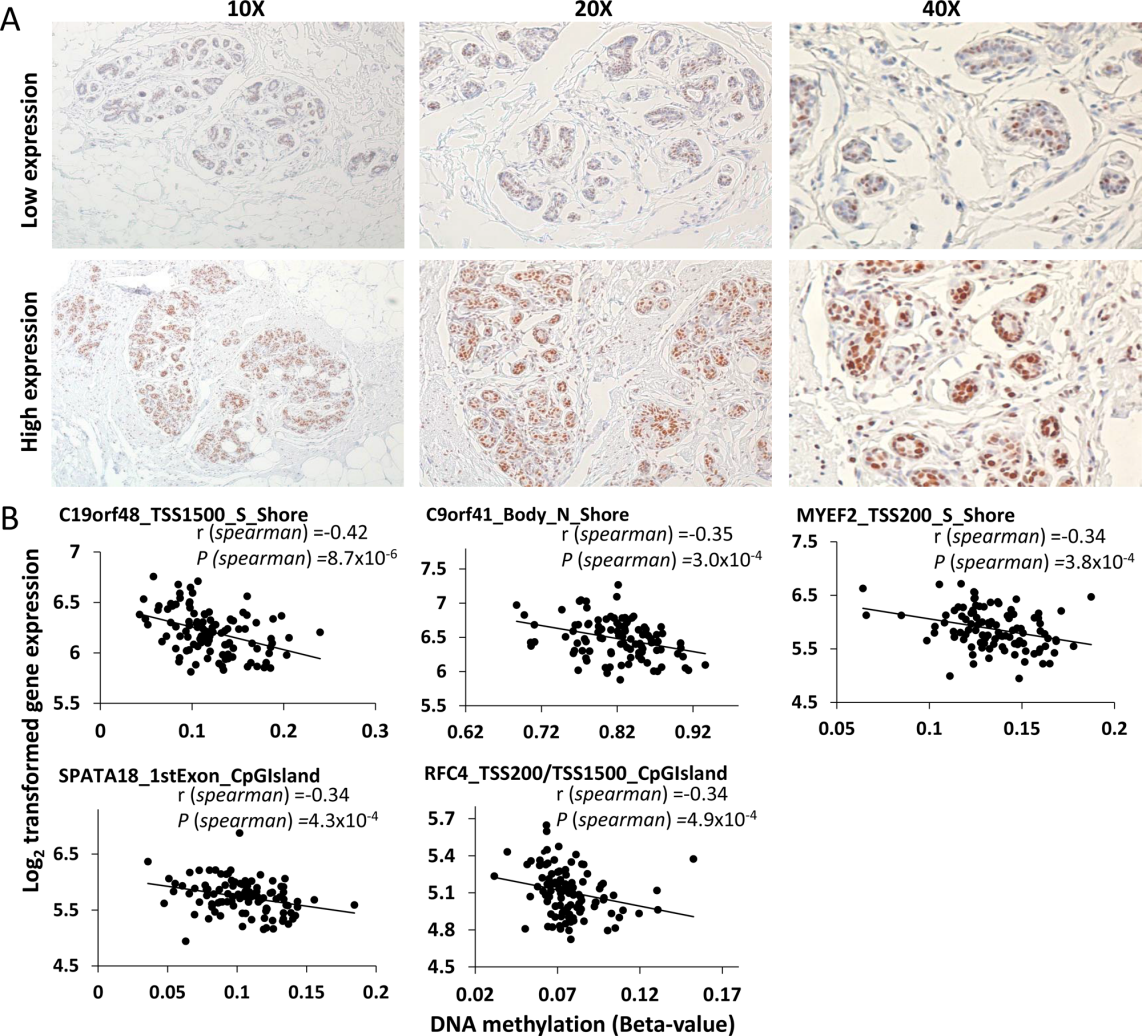
DNMT3A protein expression patterns by immunohistochemistry in histologically normal breast tissues (A) and correlations between age-related DNA methylation and gene expression (B) (A) Low expression (upper) and high expression (lower) are shown in 10X, 20X, and 40X magnification. (B) Spearman coefficient (r) and *P*-values are presented. The x- and y- axis indicate the beta-value and intensity of corresponding transcript, respectively. Gene names and functional location of aDNAm’s are shown.

Expression of DNMT3A protein varied (score = 2–6) among normal breast tissues; 43 subjects were classified as having low expression (score = 2–5) and 66 had high expression (score = 6). Examples of low and high IHC results are shown in Figure 4A. High expression of DNMT3A was significantly associated with age above the median (OR 2.43, 95% CI: 1.02–5.78; *P* = 0.04). Two aDNAm’s showed borderline significant associations with DNMT3A expression; *KRR1* (cg18557556 located in TSS1500 and S_Shore) (OR 6.57, 95% CI: 2.51–17.23; FDR *q* = 0.051)) and *DHRS12* (cg04925385 located in TSS1500 and CpG Island) (OR 6.08, 95% CI: 2.33–15.86; FDR *q* = 0.051) (Supplementary Table 3). We confirmed that age was not confounder in the association of DNMT3A expression with these two aDNAm’s.

### Correlation between aDNAm’s and their gene expression

We found a significant inverse correlation between gene expression of *C19orf48* and its methylation level (cg01534416 located in TSS1500) (r = −0.42, FDR *q* = 0.011) (Figure 4B). Another four genes showed borderline significant correlations at FDR *q* < 0.1. Three of these were located in functional promoter regions (corresponding to genes *MYEF2*, *SPATA18*, and *RFC4*) and the other was located in the gene body (*C9orf41*). All aDNAm’s were located in CpG Islands or shores and inversely correlated with gene expression (Figure 4B).

### TCGA-BRCA dataset: aDNAm’s in breast adjacent normal and tumor tissues

**Figure 5:**
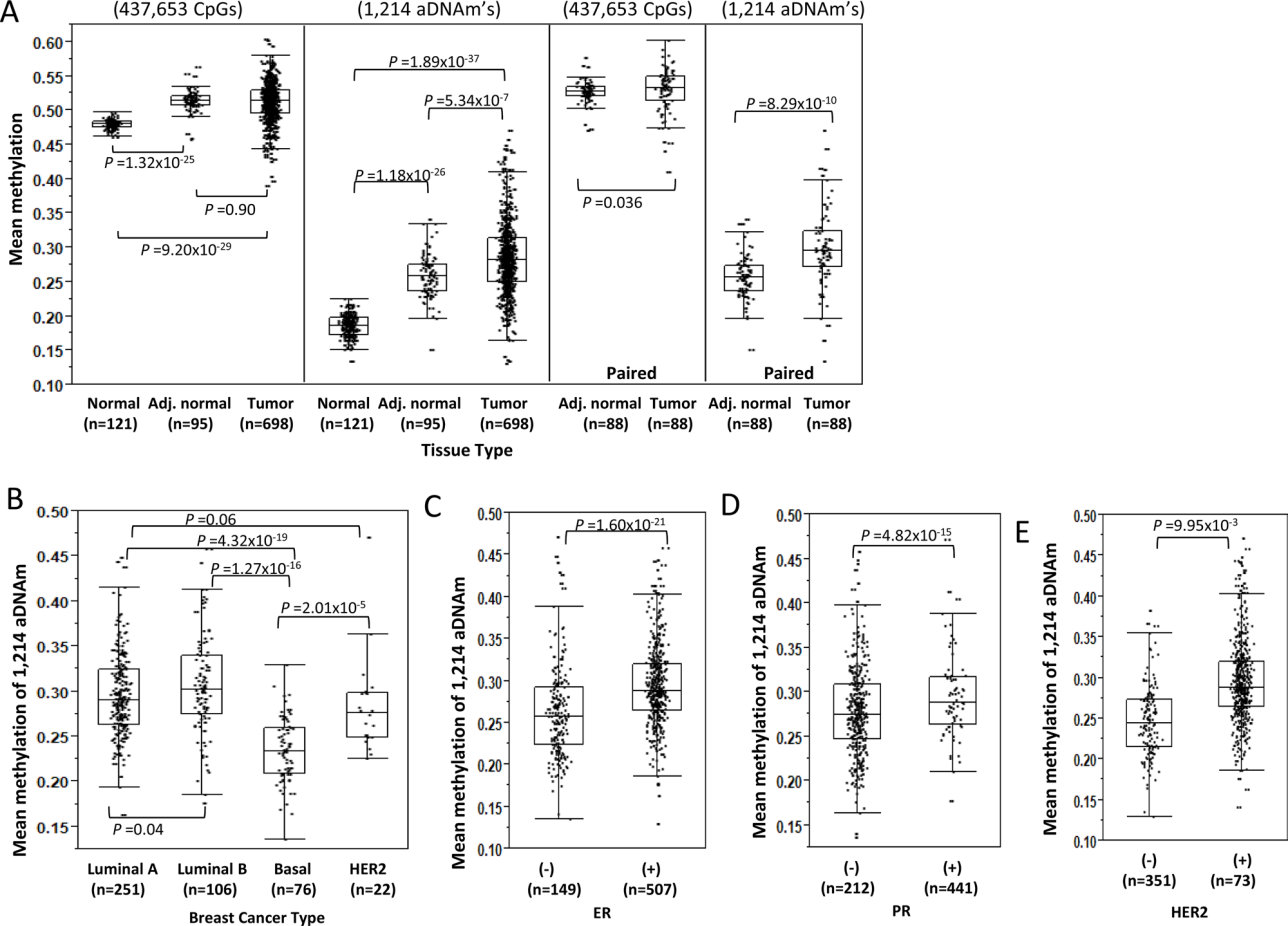
Differences of aDNAm’s by tissue types and breast cancer types (**A**) Mean methylation level for all 437,653 CpGs analyzed and mean 1,214 aDNAm’s by different tissue types. Comparison was performed between breast normal tissues from healthy women and TCGA adjacent normal and tumor. Separately, comparison for only paired adjacent normal and tumor tissues from TCGA is also shown. Comparison of mean 1,214 aDNAm’s by breast cancer types (**B**) or hormone receptor status (**C**–**E**) among TCGA tumor tissues. The differences between tissue types or cancer types were tested using Mann-Whitney rank tests.

The aDNAm’s identified for the healthy women were queried in the TCGA-BRCA data. The characteristics of TCGA samples were provided in Supplementary Table 4. The mean methylation levels across all CpG loci differed little between breast tissue from healthy women (the present study) and adjacent “normal” and tumor tissue in the TCGA dataset in both pooled and paired samples (Figure 5A). The mean aDNAm level in the breast tissues from the women without a history of cancer was 39% lower than for the TCGA adjacent normal tissues (*n* = 95) (*P* = 1.18 × 10^−26^) and 53% lower than for the TCGA tumor tissues (*n* = 698) (*P* < 1.89 × 10^−37^) (Figure 5A). An unsupervised clustering for the 1214 aDNAm’s consistently showed higher methylation of aDNAm’s among TCGA tumor compared to adjacent normal tissues, and to the breast tissues of women without a history of cancer (Supplementary Figure 1A).

The mean methylation of the 1,214 aDNAm’s was similar by tumor subtype for Luminal A (mean beta 0.29), Luminal B (mean beta 0.31), and HER2 breast cancers (mean beta 0.28), but was statistically different for Basal (mean beta 0.23) (Figure 5B, Supplementary Figure 1B). The mean methylation of the 1,214 aDNAm’s was significantly higher for tumors that were ER+, PR+, or HER2+ compared to ER-, PR-, or HER2- at *P* = 1.60 × 10^−21^, *P* = 4.82 × 10^−15^, or *P* = 9.95 × 10^−3^, respectively (Figure 5C–5E, Supplementary Figure 1C–1E).

**Figure 6:**
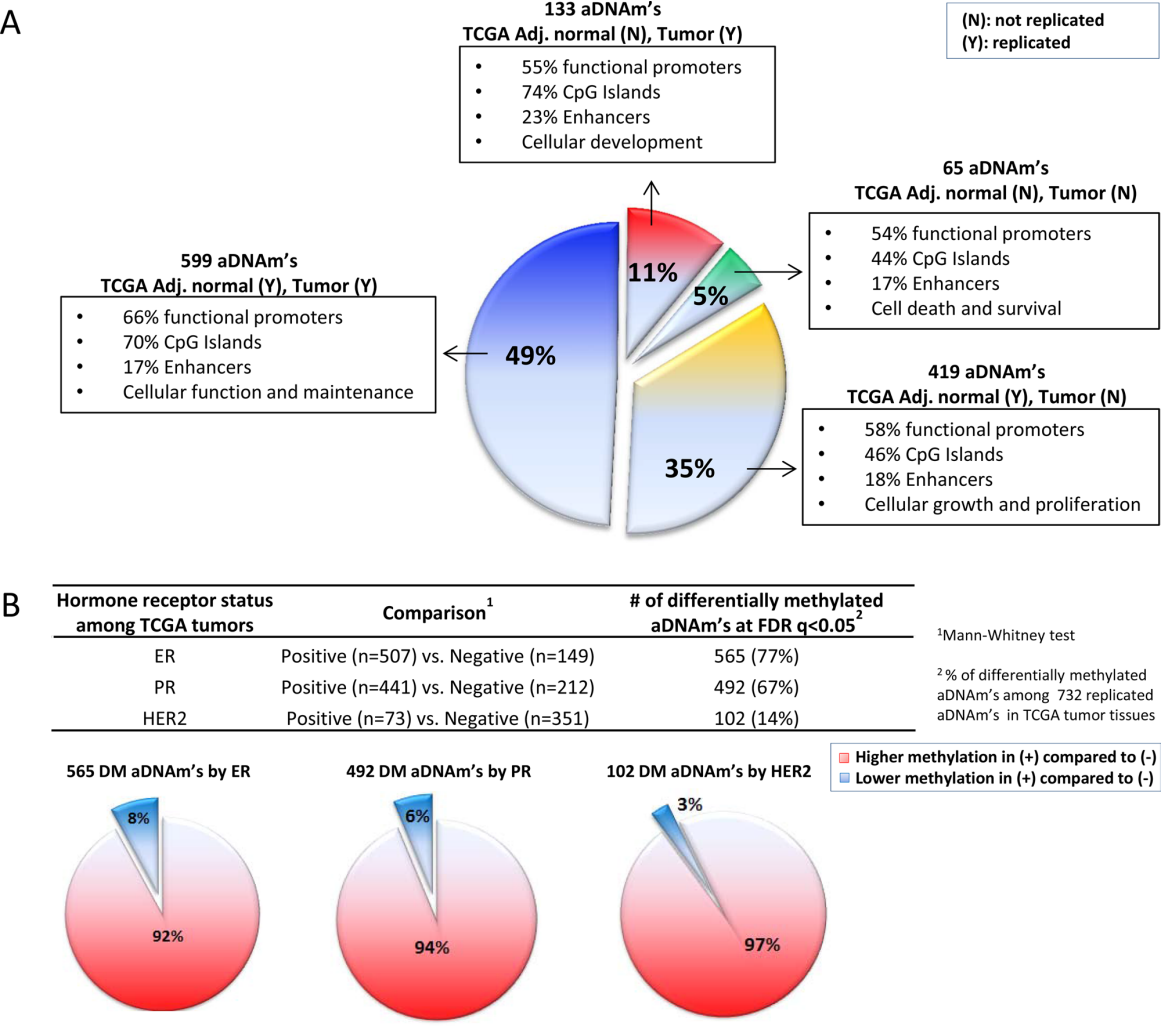
aDNAm’s in TCGA datasets (**A**) A pie chart by exploding sections for replicated (Y) or non-replicated (N) aDNAm’s in TCGA adjacent normal and/or tumor tissues. A proportion of genomic features (functional promoters, CpGIslands, enhancers) and top molecular and cellular functions by IPA for each section are shown. (**B**) Differences for methylation levels of 732 aDNAm’s replicated in TCGA tumor tissues by hormone receptor status (positive and negative). The differences were tested using Mann-Whitney rank tests and Benjamini and Hochberg False Discovery Rate (FDR) *q* < 0.05 was considered significant. In pie charts, higher and lower methylation of differentially methylated aDNAm’s in positive compared to negative hormone receptor status are shown as red and blue section, respectively.

At the locus level in the TCGA dataset, among the 1,214 aDNAm’s, 49.3% of the 1,214 aDNAm’s (*n* = 599) were associated with age in both adjacent normal and tumor tissues at FDR *q* < 0.05 (Figure 6A). These replicated aDNAm’s in both tissues were significantly enriched for CpG Islands compared to the ones that were not replicated (only observed in the breast tissues from the women without cancer) (70% vs. 44%; *P* < 0.05) (Figure 6A). The genes corresponding to these replicated aDNAm’s were predicted to be involved in cellular function and maintenance as the primary cellular functional role (Figure 6A). Also, 35% (*n* = 419) or 11% (*n* = 133) were significantly associated with age in only adjacent normal or only tumor tissues, respectively (Spearman correlations with FDR *q* < 0.05). The direction of change with age was the same for all loci.

We further examined if the 732 aDNAm’s in TCGA tumor tissues were differentially methylated by hormone receptor status. Of these aDNAm’s, 565 (77%), 492 (67%), and 102 (14%) were significantly differentially methylated by ER, PR, and HER2 status, respectively, at FDR *q* < 0.05 (Figure 6B). The majority of them (92–97%) were significantly more methylated (7–23% higher) in positive compared to negative hormone receptor tumors (Figure 6B).

The replicated aDNAm’s in the TCGA dataset and differentially methylated aDNAm’s by hormone receptor status are provided in Supplementary Table 5.

## DISCUSSION

Age is a significant and established risk factor for breast cancer [1, 2]. Gene methylation in breast cancer also changes with aging [5–7], but the extent of these changes in normal breast tissues and the direct relevance to breast cancer development is unknown. Studying aDNAm’s in normal breast tissues from women without a history of cancer or evidence of benign breast disease should provide insight for what occurs naturally in the breast over time, and potentially contribute to breast cancer development. In this study, 1,214 aDNAm’s were identified, all autosomal, most showing increased methylation with age, and usually found in CpG Islands and non-enhancers. Two aDNAm’s co-localized within vulnerable regions for somatic mutations in breast cancer. In addition to a significant inverse correlation between gene expression of *C19orf48* and its methylation level, we found another four genes to be borderline statistical significant correlations (*C9orf41*, *MYEF2*, *SPATA18*, and *RFC4*). High expression of DNMT3A protein was significantly associated with increasing age and two aDNAm’s (corresponding to *KRR1* and *DHRS12*). A majority of the genes corresponding to aDNAm’s were associated with cancer including breast cancer; the genes were highly enriched for *ESR1* or beta-estradiol responsive genes. Using the independent TCGA-BRCA dataset, we found that aDNAm’s identified in breast tissues of women without a history of breast cancer were highly methylated in breast tumors compared to both adjacent normal tissues and to the normal breast tissues. Methylation of the aDNAm’s was lower for basal tumors compared to other subtypes.

Global gene methylation on repetitive elements has been shown to be inversely correlated with aging [8, 10]. In contrast, it also is known that DNA methylation at specific loci is positively correlated with age [19]. We found a positive correlation for methylation with age for 97% of the CpGs identified; 60% of these were in CpG Islands as compared to only 32% expected by chance. In line with these findings, other studies have shown regional hypermethylation of CpG Islands in a variety of other organs [7, 11, 13, 20–22]. There also is some consistency in these findings for aDNAm’s reported in blood for a population-based longitudinal study of healthy individuals (*n* = 400) for 31% of the detected 162 aDNAm in blood [20]. However, in the only prior study of normal breast tissues that we are aware of, there was low agreement of results, (8% [16/199 aDNAm’s]), perhaps due to their using a different assay platform or small study size (*n* = 23 and *n* = 15 in two separate analyses) [7].

We found alterations in DNA methylation related to age for 803 unique genes, most corresponding to coding genes known to be involved in cancer (e.g., *TP73*, *CDKN1A* and *ESR1*). aDNAm’s involved *ESR1* and beta-estradiol responsive genes. *ESR1* is one of the well-established master transcriptional regulators in the breast [23] and is epigenetically silenced in breast cancer [24]. Some aDNAm’s were identified in genes for non-coding RNAs (ncRNAs) that may affect regulation of gene expression [25, 26] and involved in breast cancer [27, 28]. It has previously been reported that ncRNAs may be associated with aging [29]., 8 aDNAm’s were located in the promoters of precursor-miRNAs (pre-miRNAs), indicating a possible regulatory role of the aDNAm’s in miRNA expression. These include mir-15b/16–2 that targets the *BCL2* oncogene [30–32]; mir-7-3, a let-7 family member that regulates the *RAS* oncogene [33]; mir-148a, known to induce apoptosis by targeting *IGF-1R* and *IRS1* in breast cancer cells [34]; mir-426 known to promote cell proliferation in breast cancer [35]; and, mir-935 known to be differentially expressed in hormone-responsive breast cancer cells [36]. A role for two of the miRNAs (mir-596 and mir-1253) is unclear for breast cancer, but hypermethylation of these have been found in endometrial cancer cell lines [37].

None of the 1,214 aDNAm’s identified in the present study mapped to the X-chromosome, and this was also found, except for one in the 1,685 aDNAm’s identified in TCGA tumor tissues (Bonferroni corrected *P* < 0.05; data not shown). This paucity of aDNAm’s on the X chromosome is surprising considering that DNA methylation plays a key role in X chromosome inactivation [38]. However, a role in cancer seems less likely and is consistent with the small number of mutations on the X chromosome compared to autosomes [39]. Similarly, a recent study showed a significantly higher stability of X chromosome transcripts than for autosomal transcripts in various human cell lines, both male and female, and in mice [40]. Taken together, the X chromosome may be both genetically and epigenetically more stable than autosomes, even over aging, in both normal and tumor breast.

The mechanisms for changes in DNA methylation with aging are poorly understood. The *de novo* DNA methyltransferases play a key role in early development, are down-regulated in adult somatic tissues, and conversely are over-expressed in breast cancer and other tumors [41, 42]. In this study, expression of DNMT3A protein in the breast tissue was correlated with age and its expression was significantly correlated with two aDNAm’s including *KRR1* and *DHRS12*, indicating a possible mediation of DNMT3A in DNA methylation of these two CpGs during aging particularly for those aDNAm’s. A recent review suggested that *de novo* methylation related to age is involves DNA methyltransferases, consistent with the findings at least for these two CpGs herein [10].

In our study, we found only a few aDNAm’s associated with gene expression in normal breast tissue. Although we could not validate the direct relationship of DNA methylation and gene expression for most of the aDNAm’s genes, we found correlates to some of the aDNAm’s herein; gene expression was decreased for 5 aDNAm genes (*C9orf41, C19orf48, MYEF2, SPATA18, and RFC4*). Understanding how differences in aDNAm’ related to their gene expression has been challenging. This is because gene expression is regulated in many ways in addition to DNA methylation [43], and so associations are plausibly weak. For example, there are important roles for CpG Islands, promoters, and enhancers [44], and ncRNAs [45]. A recent study of normal breast tissues showed aDNAm’s enriched for regions of chromatin remodeling and transcriptional control [14], suggesting its possible contribution to gene expression. In our study, the most statistically significant correlation was found for *C19orf48*, which encodes a minor histocompatibility antigen. Although its functional role is unknown, the promoter methylation site of *C19orf48* (cg01410314) correlated with its gene expression in the current study is marked by the enhancer-related histone mark H3K27Ac based on the UCSC Genome Browser (data not shown), indicating a possible contribution of DNA methylation on its gene expression. *SPATA18* is suggested to be a novel transcriptional target of *P53* and is down-regulated in breast cancer compared to normal breast tissues, indicating a novel tumor suppressor gene [46]. *RFC4* is involved in DNA replication and chromosomal stability, and its upregulation was found in the poor prognostic group of breast cancer [47]. Given that the aDNAm’s in *SPATA18* and *RFC4* are located in *C*pG Islands, some of the aDNAm’s identified in this study may contribute to gene regulation. Although a limited number of aDNAm’s was found, another functional significance of the a subset of aDNAm’s identified that overlapped with mutation-prone sites in human cancer, mostly located in CpG Islands, shores, or shelves, indicating a possible involvement of these aDNAm’s in somatic mutations in cancers.

This study has several strengths. It provides a comprehensive analysis of aDNAm’s in breast tissues from healthy women with no previous history of breast cancer. Prior studies mostly focus on blood and other tissues, and there has been limited study in the breast [20–22]. It is unknown how aDNAm in blood and other tissues reflect aDNAm’s in the breast. Also, the study of breast tissues from women without cancer identifies aDNAm’s that may be playing a role in breast carcinogenesis, when they are found to also occur in breast tumors, as demonstrated herein. Another strength is the assessment of aDNAm’s biological effects on gene expression. Further the understanding of the association of altered DNA methylation with the expression of the DNMT3A protein adds to our understanding of these processes.

This study also has limitations. We studied breast normal tissues from women who had undergone reduction mammoplasty; these women necessarily have larger breasts than other women and have greater BMIs, possibly limiting the generalizability of these findings. However, this limitation was tempered by multivariable adjustment analysis for BMI. Further, the concordance between the findings in the healthy women and those in the TCGA data set provides some indication that the findings are more generalizable. Another limitation is that the cross-sectional study design provides evidence of association but not causality, and it is not know which of these women, if any, would develop breast cancer later in life. While a longitudinal examination of changes in methylation with aging would be ideal, it would be difficult to collect human breast tissues on multiple occasions for such a study. Also, although adipose tissue was dissected from epithelia tissue at the time of specimen collection, blunt dissection does not perfectly segregate the epithelia from adipose tissue components, so potential confounding due to cellular proportions may have entered into our determinations of aDNAm. Given the rising this concern about DNA methylation differences by cellular proportions, we assessed breast tissue heterogeneity on a subset of samples (93/121) as described in our previous study [48]. Although this assessment may not directly address the heterogeneity issue due to different biological materials used (slides for heterogeneity and tissues for DNA methylation), we found that 88% of aDNAm’s (999/1214) were not associated with the heterogeneity index (unadjusted *P* > 0.05) (Data not shown). This provides evidence that heterogeneity had little influence on the majority of aDNAm’s associated with age. Moreover, we were limited to prove biological mechanisms of aDNAm’s although we found an evidence of estrogen influences on aDNAm’s. Although cell-to-cell signaling and interactions, cell death and survival, cell morphology, cellular growth and proliferation, and gene expression functions were enriched in IPA analyses, it is possible that these analyses are biased toward genes with higher numbers of probes (average aDNAm’s = 29, average non-aDNAm’s = 18); however, we are uncertain if this biases the IPA analyses toward any particular function or regulator. Separately, we utilized an array-based method to identify aDNAm’s. Although HumanMethylation450 BeadChips provide quantitative methylation levels at a single-base resolution, the coverage of total CpGs is low (approximately 2%). Also this array does not allow the detection of allele-specific changes in DNA methylation. Thus, new methods such as next-generation sequencing will further provide large-scale methylation data without loss of information in the entire genome. Although we found the lower mean methylation of aDNAm’s identified in breast tissues of women without a history of breast cancer than those for TCGA tissues, it is possible that this reflects differences in collection and technical protocols, rather than a true biological difference.

This study is the first comprehensive report of changes in DNA methylation with age in normal breast tissues of women without a history of cancer. We found altered methylation at 1,214 aDNAm’s, almost all of which were increased methylation. The alterations were present only on autosomes. The affected genes included those known to be important in breast cancer, such as *ESR1* or beta-estradiol responsive genes. The results are consistent with the hypothesis that the relationship of aging to breast cancer may be explained at least in part by age-related changes in DNA methylation and gene expression in normal tissues before clinical cancer develops. Given that age is one of the strongest risk factors for breast cancer, understanding the mechanism of that association provides critical insights. Further understanding of the underlying mechanisms for age-related effects on DNA methylation warrants further study.

## MATERIALS AND METHODS

### Study samples

A subset of samples was from our previous study and detailed methods of this study have been described elsewhere [48–51]. Briefly, women (*n* = 121) who underwent reduction mammoplasty at Georgetown University Medical Center (Washington, DC), the University of Maryland (College Park, MD), the Washington Hospital Center (Washington, DC) and the Center for Plastic Surgery (Buffalo, NY) provided written informed consent, an epidemiologic questionnaire, blood and their residual breast tissues. Institutional Review Boards was received at each institution. Breast tissues were grossly blunt-dissected to separate epithelial tissues from adipose, snap frozen in liquid nitrogen, and stored at −80°C until use. A part of the sample was immersed in RNA later (Ambion, Inc., Austin, Texas). Women with evidence of premalignant benign breast disease were excluded. Demographic, lifestyle, reproductive, and family medical history data were assessed by an interviewer-administered questionnaire.

### Genome-wide DNA methylation analysis and quality checks

Genomic DNA was extracted from dissected frozen fresh breast tissue using a MasterPure DNA purification kit (Epicenter, Madison, WI). Following bisulfite treatment of DNA using the EZ DNA Methylation kit (Zymo Research, Irvine, CA), genome-wide DNA methylation was analyzed using HumanMethylation450 BeadChips (Illumina, San Diego, CA) (HM450), according to the manufacturer’s instructions. In order to minimize the impact of batch effects, samples were randomized by age and ancestry [52]. Illumina.idat file were imported into Partek Genomics Suite™ 6.6 (Partek Inc., St. Louis, MO) and normalized by Subset-quantile Within Array Normalization (SWAN) [53]. GRCh37/hg19 (Human Genome version 19) was used as a reference genome. Any probes with the following criteria were filtered out before further statistical analysis: detection *P* > 0.05 (*n* = 6,576), probes in Y chromosome (*n* = 416), and cross-reactive probes (*n* = 41,248) [54, 55]. An ANOVA model was used to remove the batch effect, with processing data adjusted to remove these effects. 19 out of 121 samples (about 16%) were duplicated as internal quality controls (QCs) while processing the samples. The correlation coefficient for duplicate signal intensities in the arrays was 99% (data not shown). Previously, we have shown high concordance between HM450 and gene methylation by pyrosequencing in a subset of samples used in this study [50, 51]. The HM450 data were deposited to under NCBI GEO GSE101961.

### Human transcriptome array

Total RNA was extracted from frozen breast tissue stored in RNAlater (Ambion) using 1.5 mm Triple-Pure Zirconium Beads (Benchmark Scientific, Edison, NJ) and RNeasy Plus Mini Kit (Qiagen, Valencia, CA). To profile gene expression, the GeneChip^®^ Human Transcriptome Array 2.0 (Affymetrix Inc, Santa Clara, CA) was used. The data available were limited to 104 out of 121 samples because of the RNA quality. The raw data (CEL files) were imported into the Affymetrix Expression Console^®^ Software (version 1.3.1) for log_2_ transformation and quantile normalization. Batch effect was removed as described above. Ten percent of samples were duplicated for quality control while processing the samples. The correlation coefficient for duplicate signal intensities in the arrays was 99*%* (data not shown). The Affymetrix gene expression data were deposited to under NCBI GEO GSE102088.

### Immunohistochemistry (IHC) for DNMT3A

IHC staining of DNA Methyltransferase 3 Alpha (DNMT3A) was done on formalin-fixed paraffin-embedded (FFPE) tissues (*n* = 91) using antibodies purchased from Novusbio (NBP-1–85961). Heat induced epitope retrieval was performed by immersing FFPE samples at 98°C (20 minutes) in citrate buffer (10 mM; pH 6.0) with Tween (0.05%). IHC was performed using the VectaStain Kit from Vector Labs according to manufacturer’s instructions. Slides were exposed to biotin-conjugated secondary antibodies, and counterstained with hematoxylin (Fisher, Harris Modified Hematoxylin), blued in 1% ammonium hydroxide, dehydrated, and mounted with AcryMount. Consecutive sections with the primary antibody omitted were used as negative controls. Nuclear DNMT3A staining within epithelial cells was scored by the pathologist (BVSK) using the modified Allred method (scaled 0–6). The score combined an estimated proportion score on a scale of 0 to 3 (0: negative, 1: less than 10%, 2: 10–50%, and 3: greater than 50%) with an average intensity score of 0 to 3 (0: negative, 1: weak, 2: moderate, and 3: intense). 17.5% of data (16/91) was duplicated and agreed on all intensity (16/16) and distribution scores except for a distribution score from one sample (15/16).

### The cancer genome atlas data (TCGA)

Level 1 data from TCGA-Breast Invasive Carcinoma (BRCA) database were downloaded as .idat files via https://tcga-data.nci.nih.gov/tcga/. The data were normalized using SWAN [53]. There were data for 698 women with breast cancer who were either European American or African American; 88 of those also had data from paired adjacent normal tissues. An ANOVA model was used to remove the batch effects.

### Statistical analyses

For initial identification of aDNAm’s, a Bonferroni threshold of 0.05 was used to identify the most promising signatures. For all downstream analyses, Benjamini and Hochberg False Discovery Rate (FDR) = 0.05 was used as the threshold. If not stated otherwise, a raw *P* < 0.05 was considered statistically significant.

### Locus-by-locus analysis to identify aDNAm’s

For modeling purposes, M-values were derived from Beta-values by logit-transformation. To identify aDNAm’s, analysis of covariance (ANCOVA) was used for age as a continuous variable with adjustment by race as a categorical variable (European American vs. African American), and body mass index (BMI; kg/m^2^) as a continuous variable, variables that were significantly correlated with methylation [50]. A Bonferroni-corrected *P* < 0.05 (corresponding to raw *P* < 1.14 × 10^−7^) was considered statistically significant.

### Genomic features of aDNAm’s

aDNAm*’s* were classified by genomic location based on the Illumina annotation file (HumanMethylation450_15017482_v1-2): CpG Islands, 2 kb regions upstream and downstream of the CpG Islands (shores), 2 kb regions upstream and downstream of the shores (shelves), functional promoters [within 1500 base pairs (bps) of a transcription start site (TSS) (TSS1500); within 200 bps of a TSS (TSS200); 5′ untranslated regions (5′UTR); first exon (1stExon)] and other regions [body, 3′UTR, or a stretch of DNA region located between genes (intergenic)]. To investigate potential sites prone to mutation due to DNA methylation of aDNAm’s, we used the Catalogue of Somatic Mutations in Cancer database (COSMIC) (http://cancer.sanger.ac.uk) and searched genomic locations where aDNAm’s are located in order to identify the overlap of aDNAm’s with sites prone to mutation in human cancer.

### Comparisons of distribution of aDNAm’s across the genomic location

The distribution of aDNAm’s across genomic location (CpG Island, functional location, and enhancer) was compared to the distribution of total CpGs analyzed (*n* = 437,653). The enhancers are derived from the Illumina annotation [56] based on enhancer elements determined by the Encyclopedia of DNA Elements (ENCODE). A chi-square raw *P* < 0.05 was considered statistically significant.

### Correlation between methylation of aDNAm’s and gene expression

Probes from HM450 and Affymetrix were matched for gene names (perfectly matching). The CpGs located up to 1500 bps upstream of the gene, gene body, and 3′UTR were included in the analysis. The aDNAm (M-value) was correlated with expression of a corresponding gene (mean of log_2_ transformed intensities if there was more than one probe) by Spearman correlation. FDR *q* < 0.05 (corresponding to raw *P* < 8.66 × 10^−6^) were considered significant.

### Correlation of DNMT3A expression with age or aDNAm’s

A logistic regression model was used because of the skewed distribution of DNMT3A expression (data not shown). Median values were used to dichotomize age (37 years) and aDNAm’s. DNMT3A by IHC was dichotomized using the median Allred score as the cut-point: ≤ 5 = lower expression (*n* = 43) and 6 = higher expression (*n* = 66). Logistic regression models were used to estimate odds ratios (OR) and 95% confidence intervals (CI) for associations of DNMT3A protein expression with age at a raw *P* < 0.05 or with methylation levels of aDNAm’s at FDR *q* < 0.05 (corresponding to raw *P* < 4.22 × 10^−5^).

### aDNAm’s in the TCGA dataset

Mann-Whitney rank tests were performed to compare mean methylation levels between groups (normal, pooled adjacent normal, pooled tumor, paired normal, and paired tumor tissues) and between different breast cancer types. A raw *P* < 0.05 was considered statistically significant. To assess aDNAm’s identified from normal breast tissues in TCGA samples, *M*-values of 1,214 CpGs were first filtered from TCGA datasets and correlated with age using the Spearman correlation. Spearman correlation FDR *q* < 0.05 were considered statistically significant.

### Ingenuity pathway analysis (IPA)

The unique gene list corresponding to aDNAm’s was created and used for IPA. The imported genes were classified by IPA (Ingenuity^®^ Systems, www.ingenuity.com) using the biological functions to be presented as being used for annotation, ranked by score. The score [score = −log_10_(*p*-value)] computed by IPA is a measure of the probability of finding identified genes in a set of a list of biological functions stored in the IPA knowledge base (IPKB).

## SUPPLEMENTARY MATERIALS FIGURE AND TABLES









## Abbreviations

aDNAm: age-related DNA methylation
1stExon: first exon
ANCOVA: analysis of covariance
bps: base pairs
BRCA: Breast Invasive Carcinoma
COSMIC: Catalogue of Somatic Mutations in Cancer database
DNMT3A: DNA Methyltransferase 3 Alpha
FDR: False Discovery Rate
FFPE: formalin-fixed paraffin-embedded
HM450: HumanMethylation450 BeadChips
IHC: Immunohistochemistry
lncRNAs: long-noncoding RNAs
OR: odds ratios
QCs: quality controls
shelves: 2 kb regions upstream and downstream of the shores
shores: 2 kb regions upstream and downstream of the CpG Islands
SWAN: Subset-quantile Within Array Normalization
TCGA: The Cancer Genome Atlas
TSS: transcription start site
TSS1500: within 1500 base pairs (bps) of a transcription start site
TSS200: within 200 bps of a TSS
UTR: untranslated regions.
