# Supplementary material for: Landscape of genome-wide age-related DNA methylation in breast tissue
Source: Oncotarget. 2017 Nov 29;8(70):114648–62. doi: 10.18632/oncotarget.22754 (PMC5777721; doi:10.18632/oncotarget.22754)
Supplement: Supplementary file 1 [file oncotarget-08-114648-s001.pdf]

Landscape of genome-wide age-related DNA methylation in breast tissue

SUPPLEMENTARY MATERIALS

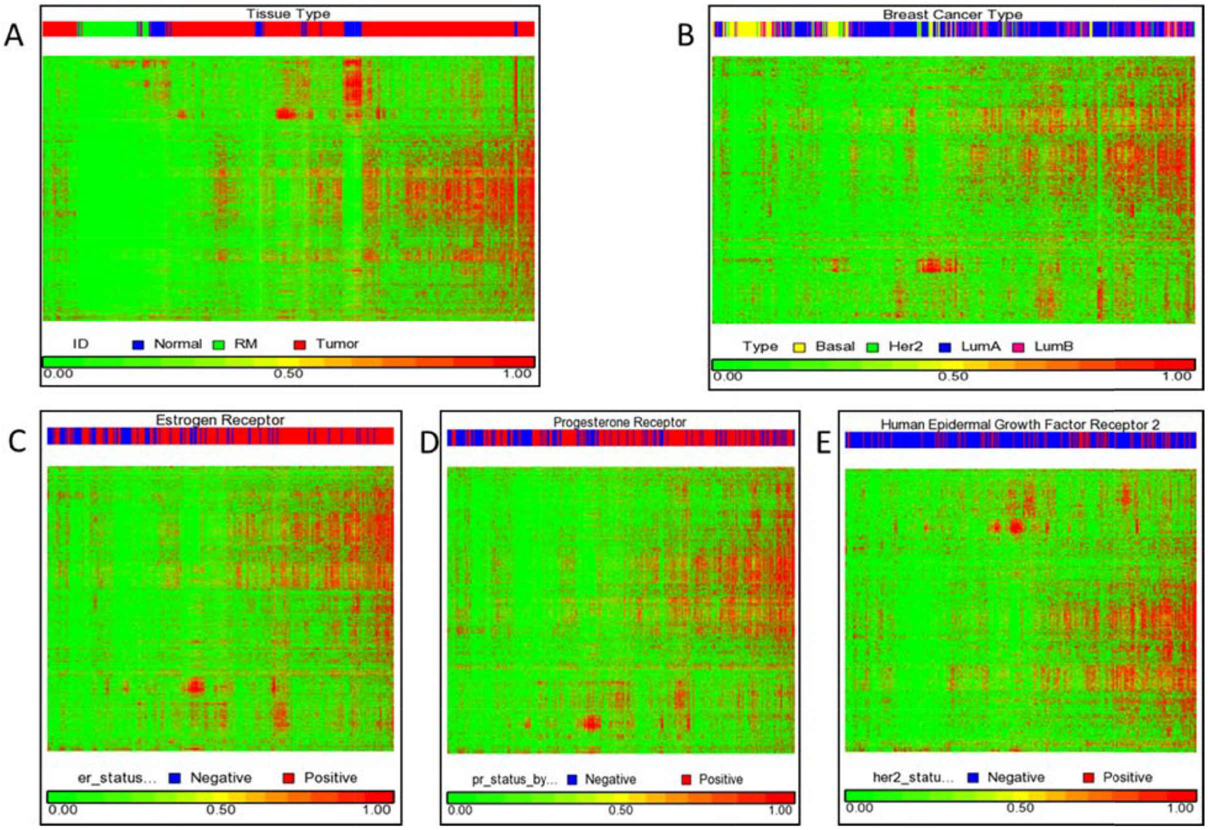

Supplementary Figure 1: Unsupervised hierarchical clustering of beta-values for 1214 age-related DNA methylation identified from normal breast tissues (RM) from healthy women. The clusters for 1214 aDNAm were performed by (**A**) tissue types; RM (green, *n* = 121) , 95 adjacent normal (Normal, blue, *n* = 95), and Tumor tissues (red, *n* = 698), (**B**) breast cancer types among tumors ;Lumina A (blue, *n* = 251), Luminal B (red, *n* = 106), Negative/basal-like (yellow, *n* = 76), and HER2 (green, *n* = 22), and (**C–E**) hormone receptor status for ER (red for *n* = 507 positive and blue for *n* = 149 negative), PR (red for *n* = 441 positive and blue for *n* = 212 negative), and HER 2 (red for n=73 positive and blue for n=351 negative).

Supplementary Table 1: A list of 1,214 age-related DNA methylation identified from normal breast tissues from healthy women (*n* = 121). See_Supplementary_Table 1

Supplementary Table 2: The overlapping genomic location of aDNAm's with sites prone to mutation in human cancer

Target ID	Overlapping gene	Chromosome	Location	CpG Island	Enhancer	Functional location	COSMIC ID	CDS Mutation	Tumor Site
**Loss methylation by age**									
cg19955284	SLC44A4	6	31833747	S_Shelf	NO	Body	COSM241655	c.1390G > A (Substitution)	Prostate and Liver
**Gain methylation by age**									
cg02355885	ALK	2	30142990	Island	NO	1stExon	COSM1182534	c.536G > A (Substitution)	Large intestine
cg23681664	CPNE9	3	9746637	Island	YES	Body	COSM4158685	c.220G > T (Substitution)	Thyroid
cg01844642	GPR62	3	51989764	Island	YES	1stExon	COSM1424329	c.96C > T (Substitution)	Large intestine
cg18267374	NEFM	8	24771273	Island	NO	TSS1500; 5'UTR;1stExon	COSM33641	c.1-98_12del110 (Deletion - intronic)	**Breast**
cg07502389	NEFM	8	24771259	Island	NO	TSS200; TSS1500	COSM33641	c.1-98_12del110 (Deletion - intronic)	**Breast**
cg14585700	PAX5*	9	37027605	Island	YES	Body	COSM87055	c.1-?_410+?del (Deletion)	Haematopoietic and Lymphoid
cg14484688	LRRC8A	9	131669841	N_Shore	NO	Body	COSM3327812	c.398C > T (Substitution)	Central nervous system
cg03799530	SH2B3	12	111843215	Island	NO	TSS1500	COSM1235306	c.1_733-7575del21033 (Deletion - intronic)	Haematopoietic and Lymphoid
cg12639558	KDM2B	12	121958832	Opensea	NO	Body	COSM144690	c.1002C > G (Substitution)	Haematopoietic and Lymphoid
cg24051481	SOX21*	13	95364062	Island	NO	1stExon	COSM948934	c.242G > A (Substitution)	Endometrium
cg00991848	RPS2	16	2014270	Island	NO	Body	COSM1609007	c.267+6C > A (Substitution - intronic)	Liver
cg08317252	STK11	19	1223163	N_Shelf	NO	Body	COSM21358	c.1100C > T (Substitution)	Large intestine
cg21183502	PPP1R3D	20	58514581	Island	NO	1stExon	COSM4135261	c.406G > A (Substitution)	Pancreas
cg19841423	ZGPAT*	20	62366755	S_Shore	NO	Body	COSM238471	c.1296C > T (Substitution)	Prostate

*Transcription regulator
Chr Chromosome

Supplementary Table 3: The association of DNMT3A protein expression with 1,214 aDNAm's.

See_Supplementary_Table 3

Supplementary Table 4: Characteristics of TCGA-breast invasive carcinoma (BRCA) datasets

Characteristic	Adjacent Normal (*n* = 95), Mean (SD) or *N* (%)		Tumors (*n* = 698), Mean (SD) or *N* (%)	
Age, years	57.7	(15.2)	58.3	(13.1)
Race				
European American	91	(96%)	552	(79%)
African American	4	(4%)	146	(21%)
Menopausal status				
Pre-menopausal	25	(31%)	155	(26%)
Post-menopausal	55	(69%)	450	(74%)
Missing	15		93	
AJCC Stage				
Early (I, IIA, IIB, IIIA)	-	-	621	(89%)
Late (IIIB, IV, V)	-	-	75	(11%)
Missing	-	-	2	
Tumor type				
Luminal A	-	-	251	(55%)
Luminal B	-	-	106	(23%)
Basal	-	-	76	(17%)
HER2	-	-	22	(5%)
Missing	-	-	338	
ER				
Positive	-	-	507	(77%)
Negative	-	-	149	(23%)
Missing	-	-	42	
PR				
Positive	-	-	441	(68%)
Negative	-	-	212	(32%)
Missing	-	-	45	
HER2	-	-		
Positive	-	-	73	(17%)
Negative	-	-	351	(89%)
Missing	-	-	274	

Supplementary Table 5: A list of replicated aDNAm's in the TCGA dataset and differentially methylated aDNAm's by hormone receptor status. See_Supplementary_Table 5
