# Supplementary material for: Landscape of genome-wide age-related DNA methylation in breast tissue
Source: Oncotarget. 2017 Nov 29;8(70):114648–62. doi: 10.18632/oncotarget.22754 (PMC5777721; doi:10.18632/oncotarget.22754)
Supplement: Supplementary file 2 [file oncotarget-08-114648-s002.docx]

| **Loss/gain methylation by age** | **Probeset ID** | **Chromosome** | **Genomic Location** | **ENHANCER** | **CpG Isand and neighbors** | **UCSC_REFGENE_ACCESSION** | **UCSC_REFGENE_GROUP** | **UCSC_REFGENE_NAME** | **p-value** | **bonferroni p-value** | **PartialCorr** |
| --- | --- | --- | --- | --- | --- | --- | --- | --- | --- | --- | --- |
| Gain | cg04684267 | 19 | 58694502 |  | Island | NM_016325;NM_016325;NM_133502;NM_133502;NM_016324;NM_016324 | 1stExon;5'UTR;1stExon;5'UTR;5'UTR;1stExon | ZNF274;ZNF274;ZNF274;ZNF274;ZNF274;ZNF274 | 2.69E-27 | 1.18E-21 | 0.78 |
| Gain | cg03545227 | 2 | 220173100 |  | Island | NM_002846 | Body | PTPRN | 2.95E-27 | 1.29E-21 | 0.78 |
| Gain | cg04880546 | 12 | 120868468 |  |  |  |  |  | 7.90E-27 | 3.46E-21 | 0.78 |
| Gain | cg26158959 | 1 | 210111162 |  | N_Shore | NM_001146261;NR_027458;NR_027459;NM_001146264;NM_153262;NM_001146262 | TSS1500;TSS1500;TSS1500;TSS1500;TSS1500;TSS1500 | SYT14;SYT14;SYT14;SYT14;SYT14;SYT14 | 8.53E-27 | 3.73E-21 | 0.77 |
| Gain | cg03036557 | 13 | 92050720 |  | N_Shore | NM_004466 | TSS1500 | GPC5 | 9.23E-27 | 4.04E-21 | 0.78 |
| Gain | cg23606718 | 2 | 131513927 |  | Island | NM_152698;NM_001105194;NM_001105195;NM_001105194;NM_001105193;NM_001105195 | 5'UTR;5'UTR;1stExon;1stExon;5'UTR;5'UTR | FAM123C;FAM123C;FAM123C;FAM123C;FAM123C;FAM123C | 2.45E-26 | 1.07E-20 | 0.76 |
| Gain | cg26830108 | 7 | 100813299 |  | N_Shelf |  |  |  | 8.09E-26 | 3.54E-20 | 0.75 |
| Gain | cg06458239 | 19 | 58038573 |  | Island | NM_153263 | TSS200 | ZNF549 | 1.11E-25 | 4.87E-20 | 0.77 |
| Gain | cg07303143 | 3 | 44803452 |  | Island | NM_020696;NM_020242 | TSS1500;Body | KIAA1143;KIF15 | 1.19E-25 | 5.19E-20 | 0.76 |
| Gain | cg06580318 | 2 | 169747119 |  | S_Shore | NM_020675 | TSS200 | SPC25 | 3.05E-25 | 1.34E-19 | 0.76 |
| Gain | cg15789607 | 19 | 4769690 |  |  | NR_029607;NR_027148 | TSS1500;Body | MIR7-3;C19orf30 | 2.90E-24 | 1.27E-18 | 0.75 |
| Gain | cg23854009 | 19 | 58111128 |  | N_Shore | NM_020880 | TSS200 | ZNF530 | 3.73E-24 | 1.63E-18 | 0.76 |
| Gain | cg10729426 | 19 | 58038585 |  | Island | NM_153263 | TSS200 | ZNF549 | 9.78E-24 | 4.28E-18 | 0.75 |
| Gain | cg26921969 | 5 | 92948217 |  |  |  |  |  | 1.46E-23 | 6.40E-18 | 0.73 |
| Gain | cg02331561 | 16 | 2391081 |  | Island | NR_003574;NM_001089 | Body;TSS1500 | ABCA17P;ABCA3 | 2.50E-23 | 1.09E-17 | 0.74 |
| Gain | cg23156348 | 11 | 124981869 |  | S_Shore |  |  |  | 2.57E-23 | 1.12E-17 | 0.74 |
| Gain | cg12678562 | 13 | 92050726 |  | N_Shore | NM_004466 | TSS1500 | GPC5 | 2.74E-23 | 1.20E-17 | 0.74 |
| Gain | cg23813012 | 1 | 14026482 |  | Island | NM_001135610 | TSS1500 | PRDM2 | 3.93E-23 | 1.72E-17 | 0.72 |
| Gain | cg25743481 | 3 | 44803289 |  | Island | NM_020242;NM_020242;NM_020696 | 1stExon;5'UTR;TSS200 | KIF15;KIF15;KIAA1143 | 6.45E-23 | 2.82E-17 | 0.73 |
| Gain | cg19401340 | 17 | 56833197 |  | Island | NM_014906 | TSS200 | PPM1E | 8.37E-23 | 3.66E-17 | 0.75 |
| Gain | cg22736354 | 6 | 18122719 | TRUE | Island | NM_198586 | 1stExon | NHLRC1 | 9.18E-23 | 4.02E-17 | 0.74 |
| Gain | cg08715791 | 11 | 66189297 |  | Island | NM_178864 | Body | NPAS4 | 1.36E-22 | 5.95E-17 | 0.72 |
| Gain | cg15995695 | 1 | 179334675 |  | N_Shore | NM_182766;NM_144696 | TSS1500;TSS200 | C1orf125;C1orf125 | 1.39E-22 | 6.08E-17 | 0.73 |
| Gain | cg05917988 | 6 | 44281197 |  | Island | NM_020745 | TSS200 | AARS2 | 1.40E-22 | 6.13E-17 | 0.73 |
| Gain | cg10943458 | 3 | 120627085 |  | Island | NM_014980;NM_014980 | 1stExon;5'UTR | STXBP5L;STXBP5L | 1.81E-22 | 7.91E-17 | 0.72 |
| Gain | cg23428445 | 7 | 124405467 |  | Island | NM_005302;NM_005302 | 1stExon;5'UTR | GPR37;GPR37 | 1.88E-22 | 8.22E-17 | 0.73 |
| Gain | cg00252781 | 1 | 179334658 |  | N_Shore | NM_182766;NM_144696 | TSS1500;TSS200 | C1orf125;C1orf125 | 1.96E-22 | 8.59E-17 | 0.74 |
| Gain | cg12141030 | 3 | 44803447 |  | Island | NM_020696;NM_020242 | TSS1500;Body | KIAA1143;KIF15 | 3.09E-22 | 1.35E-16 | 0.72 |
| Gain | cg08097417 | 7 | 130419133 |  | Island | NM_138693 | TSS1500 | KLF14 | 4.38E-22 | 1.92E-16 | 0.72 |
| Gain | cg00702638 | 3 | 44803293 |  | Island | NM_020242;NM_020242;NM_020696 | 1stExon;5'UTR;TSS200 | KIF15;KIF15;KIAA1143 | 6.03E-22 | 2.64E-16 | 0.73 |
| Gain | cg10778288 | 12 | 113917994 |  | Island |  |  |  | 1.12E-21 | 4.92E-16 | 0.71 |
| Gain | cg12373771 | 22 | 17601381 |  | Island | NM_031890;NM_001163079 | 1stExon;5'UTR | CECR6;CECR6 | 1.26E-21 | 5.51E-16 | 0.72 |
| Gain | cg21623865 | 3 | 9746317 | TRUE | Island | NM_153635 | Body | CPNE9 | 1.48E-21 | 6.49E-16 | 0.72 |
| Gain | cg10548038 | 13 | 92050731 |  | N_Shore | NM_004466 | TSS1500 | GPC5 | 1.89E-21 | 8.25E-16 | 0.73 |
| Gain | cg18468088 | 6 | 35490818 |  |  |  |  |  | 2.46E-21 | 1.08E-15 | 0.72 |
| Gain | cg00088042 | 10 | 103892309 |  | N_Shore | NM_015062 | TSS1500 | PPRC1 | 2.55E-21 | 1.12E-15 | 0.70 |
| Gain | cg14674720 | 2 | 219827930 | TRUE | Island |  |  |  | 3.02E-21 | 1.32E-15 | 0.71 |
| Gain | cg15936446 | 5 | 42952369 |  | Island |  |  |  | 3.48E-21 | 1.52E-15 | 0.73 |
| Gain | cg05708550 | 5 | 137688227 |  | Island | NM_016604 | TSS200 | KDM3B | 4.55E-21 | 1.99E-15 | 0.73 |
| Gain | cg01122755 | 6 | 44281249 |  | S_Shore | NM_020745 | TSS200 | AARS2 | 9.86E-21 | 4.32E-15 | 0.70 |
| Gain | cg15103195 | 7 | 100797603 |  | Island | NM_001283 | TSS200 | AP1S1 | 4.33E-20 | 1.89E-14 | 0.70 |
| Gain | cg10806820 | 3 | 48699090 |  | Island | NM_001407 | 1stExon | CELSR3 | 4.35E-20 | 1.90E-14 | 0.70 |
| Gain | cg19291355 | 6 | 44281188 |  | Island | NM_020745 | TSS200 | AARS2 | 4.74E-20 | 2.07E-14 | 0.71 |
| Gain | cg25090514 | 5 | 2038743 |  | Island |  |  |  | 8.04E-20 | 3.52E-14 | 0.71 |
| Gain | cg02650266 | 4 | 147558239 |  | Island |  |  |  | 9.38E-20 | 4.11E-14 | 0.70 |
| Gain | cg22331349 | 19 | 52391350 |  | Island | NR_024181;NM_032679;NM_001135590 | TSS200;TSS200;TSS200 | ZNF577;ZNF577;ZNF577 | 9.61E-20 | 4.21E-14 | 0.67 |
| Gain | cg18569335 | 17 | 40171970 |  | Island | NM_001144927;NM_001144929;NM_001144928;NM_017595;NM_001001349 | TSS200;TSS200;TSS200;TSS200;5'UTR | NKIRAS2;NKIRAS2;NKIRAS2;NKIRAS2;NKIRAS2 | 9.66E-20 | 4.23E-14 | 0.69 |
| Gain | cg27320127 | 2 | 47798396 |  | Island | NM_022055 | TSS1500 | KCNK12 | 9.74E-20 | 4.26E-14 | 0.71 |
| Gain | cg00460776 | 12 | 120731287 |  |  |  |  |  | 1.02E-19 | 4.47E-14 | 0.70 |
| Gain | cg14704921 | 4 | 53728654 |  | Island | NM_023940;NM_023940 | 1stExon;5'UTR | RASL11B;RASL11B | 1.10E-19 | 4.81E-14 | 0.70 |
| Gain | cg26792755 | 7 | 140714919 |  | Island | NM_053035;NM_016071 | TSS200;TSS1500 | MRPS33;MRPS33 | 1.38E-19 | 6.04E-14 | 0.70 |
| Gain | cg25427880 | 10 | 102322128 | TRUE | Island |  |  |  | 1.50E-19 | 6.55E-14 | 0.70 |
| Gain | cg14633456 | 4 | 113445269 |  | Island |  |  |  | 2.02E-19 | 8.83E-14 | 0.70 |
| Gain | cg16867657 | 6 | 11044877 |  | Island | NM_017770 | TSS1500 | ELOVL2 | 2.08E-19 | 9.10E-14 | 0.69 |
| Gain | cg06121469 | 15 | 44956098 |  | S_Shore | NM_025137;NM_001160227 | TSS1500;TSS1500 | SPG11;SPG11 | 4.15E-19 | 1.82E-13 | 0.70 |
| Gain | cg25410668 | 1 | 28241577 |  | S_Shore | NM_002946 | TSS1500 | RPA2 | 6.07E-19 | 2.66E-13 | 0.70 |
| Gain | cg02383785 | 7 | 127808848 |  | Island |  |  |  | 6.92E-19 | 3.03E-13 | 0.70 |
| Gain | cg27665659 | 7 | 100797595 |  | Island | NM_001283 | TSS200 | AP1S1 | 7.90E-19 | 3.46E-13 | 0.68 |
| Gain | cg08904363 | 7 | 102790119 |  | Island | NM_198990;NM_001122838 | TSS1500;TSS1500 | NAPEPLD;NAPEPLD | 8.13E-19 | 3.56E-13 | 0.69 |
| Gain | cg19996355 | 19 | 19729375 | TRUE | Island | NM_025245 | 1stExon | PBX4 | 8.53E-19 | 3.74E-13 | 0.69 |
| Gain | cg24888989 | 3 | 44803291 |  | Island | NM_020242;NM_020242;NM_020696 | 1stExon;5'UTR;TSS200 | KIF15;KIF15;KIAA1143 | 9.42E-19 | 4.12E-13 | 0.68 |
| Gain | cg11229185 | 10 | 22625274 |  | Island |  |  |  | 1.14E-18 | 5.00E-13 | 0.69 |
| Gain | cg22454769 | 2 | 106015767 |  | Island | NM_001039492;NM_001450;NM_201557;NM_201555 | TSS200;TSS200;5'UTR;TSS200 | FHL2;FHL2;FHL2;FHL2 | 1.35E-18 | 5.92E-13 | 0.68 |
| Gain | cg11075316 | 3 | 160167725 |  | Island | NM_173084 | TSS200 | TRIM59 | 1.44E-18 | 6.29E-13 | 0.69 |
| Gain | cg11566154 | 6 | 80579376 |  |  |  |  |  | 2.33E-18 | 1.02E-12 | 0.69 |
| Gain | cg14063191 | 9 | 131486742 |  | Island | NM_032799 | TSS1500 | ZDHHC12 | 2.55E-18 | 1.12E-12 | 0.68 |
| Gain | cg20893579 | 22 | 38215064 |  | Island |  |  |  | 2.71E-18 | 1.19E-12 | 0.69 |
| Gain | cg01908954 | 15 | 51973764 |  | Island | NM_001165257;NM_013243;NM_013243;NM_001165257 | 5'UTR;1stExon;5'UTR;1stExon | SCG3;SCG3;SCG3;SCG3 | 2.82E-18 | 1.23E-12 | 0.68 |
| Gain | cg17758721 | 20 | 2451208 |  | Island | NM_198216;NM_003091 | Body;Body | SNRPB;SNRPB | 4.38E-18 | 1.92E-12 | 0.68 |
| Gain | cg24079702 | 2 | 106015771 |  | Island | NM_001039492;NM_001450;NM_201557;NM_201555 | TSS200;TSS200;5'UTR;TSS200 | FHL2;FHL2;FHL2;FHL2 | 4.60E-18 | 2.01E-12 | 0.67 |
| Gain | cg06737494 | 3 | 172167644 | TRUE | Island | NM_198407;NM_004122 | TSS1500;TSS1500 | GHSR;GHSR | 7.00E-18 | 3.06E-12 | 0.66 |
| Gain | cg23193759 | 10 | 71389896 |  | Island | NM_145306 | TSS200 | C10orf35 | 1.04E-17 | 4.54E-12 | 0.68 |
| Gain | cg11614451 | 3 | 160167729 |  | Island | NM_173084 | TSS200 | TRIM59 | 1.08E-17 | 4.74E-12 | 0.67 |
| Gain | cg14911690 | 19 | 19729395 | TRUE | Island | NM_025245 | 1stExon | PBX4 | 1.15E-17 | 5.02E-12 | 0.68 |
| Gain | cg23027580 | 8 | 67089513 |  | Island | NM_000756 | Body | CRH | 1.21E-17 | 5.28E-12 | 0.67 |
| Gain | cg09941452 | 7 | 97557827 |  | Island |  |  |  | 1.53E-17 | 6.70E-12 | 0.67 |
| Gain | cg11667101 | 10 | 105127581 |  | Island | NM_006951 | TSS200 | TAF5 | 1.59E-17 | 6.94E-12 | 0.67 |
| Gain | cg01789150 | 1 | 28241495 |  | Island | NM_002946 | TSS1500 | RPA2 | 1.90E-17 | 8.31E-12 | 0.67 |
| Gain | cg06639320 | 2 | 106015739 |  | Island | NM_001039492;NM_001450;NM_201557;NM_201555 | TSS200;TSS200;5'UTR;TSS200 | FHL2;FHL2;FHL2;FHL2 | 2.79E-17 | 1.22E-11 | 0.65 |
| Gain | cg10625705 | 20 | 307320 |  | Island | NM_006943 | 1stExon | SOX12 | 2.80E-17 | 1.22E-11 | 0.66 |
| Gain | cg05602648 | 1 | 230778078 |  | Island | NM_001145036;NM_007357 | TSS200;TSS200 | COG2;COG2 | 3.00E-17 | 1.31E-11 | 0.67 |
| Gain | cg04084157 | 7 | 100809049 |  | Island | NM_003378 | TSS200 | VGF | 3.04E-17 | 1.33E-11 | 0.67 |
| Gain | cg21927946 | 19 | 4769688 |  |  | NR_029607;NR_027148 | TSS1500;Body | MIR7-3;C19orf30 | 3.25E-17 | 1.42E-11 | 0.67 |
| Gain | cg07066898 | 19 | 17717094 |  | Island | NM_001080421 | Body | UNC13A | 3.47E-17 | 1.52E-11 | 0.66 |
| Gain | cg04911280 | 6 | 44281184 |  | Island | NM_020745 | TSS200 | AARS2 | 4.18E-17 | 1.83E-11 | 0.67 |
| Gain | cg08614301 | 19 | 19729716 | TRUE | Island | NM_025245 | TSS1500 | PBX4 | 5.66E-17 | 2.48E-11 | 0.67 |
| Gain | cg12589298 | 19 | 50828905 |  | Island | NM_004977 | Body | KCNC3 | 6.10E-17 | 2.67E-11 | 0.66 |
| Gain | cg06993413 | 15 | 65810204 |  | S_Shore | NM_017743;NM_197961;NM_197960;NM_130434 | TSS200;TSS200;TSS1500;TSS200 | DPP8;DPP8;DPP8;DPP8 | 6.93E-17 | 3.03E-11 | 0.64 |
| Gain | cg07850604 | 14 | 36003443 |  | Island | NM_032594;NM_032594 | 5'UTR;1stExon | INSM2;INSM2 | 8.59E-17 | 3.76E-11 | 0.66 |
| Gain | cg16368750 | 2 | 71503548 |  | Island |  |  |  | 8.59E-17 | 3.76E-11 | 0.65 |
| Gain | cg13612447 | 17 | 2653188 |  | Island |  |  |  | 8.68E-17 | 3.80E-11 | 0.66 |
| Gain | cg14676592 | 16 | 49910862 | TRUE | Island |  |  |  | 1.16E-16 | 5.09E-11 | 0.66 |
| Gain | cg16477091 | 17 | 56833000 |  | Island | NM_014906 | TSS1500 | PPM1E | 1.19E-16 | 5.21E-11 | 0.66 |
| Gain | cg11908384 | 11 | 122852698 | TRUE | Island | NM_001098169 | TSS1500 | BSX | 1.31E-16 | 5.73E-11 | 0.66 |
| Gain | cg21801378 | 15 | 72612125 |  | Island | NM_052840 | 1stExon | BRUNOL6 | 1.57E-16 | 6.87E-11 | 0.65 |
| Gain | cg17110586 | 19 | 36454623 |  | S_Shelf |  |  |  | 1.57E-16 | 6.88E-11 | 0.64 |
| Gain | cg19343530 | 6 | 28831388 |  | N_Shore |  |  |  | 1.60E-16 | 7.01E-11 | 0.65 |
| Gain | cg14064148 | 19 | 10527576 |  | Island |  |  |  | 1.80E-16 | 7.88E-11 | 0.65 |
| Gain | cg26522319 | 1 | 111506511 |  | Island | NM_001006945;NM_018372;NM_001006945;NM_018372 | 1stExon;5'UTR;5'UTR;1stExon | C1orf103;C1orf103;C1orf103;C1orf103 | 2.03E-16 | 8.86E-11 | 0.64 |
| Gain | cg20413454 | 1 | 53163625 |  | N_Shore | NM_023077 | Body | C1orf163 | 2.30E-16 | 1.01E-10 | 0.64 |
| Gain | cg20638016 | 11 | 122852694 | TRUE | Island | NM_001098169 | TSS1500 | BSX | 2.64E-16 | 1.15E-10 | 0.64 |
| Gain | cg12265959 | 8 | 11324687 |  | Island | NM_053279 | TSS1500 | FAM167A | 3.10E-16 | 1.36E-10 | 0.64 |
| Gain | cg02721182 | 6 | 28805254 |  | N_Shore |  |  |  | 3.38E-16 | 1.48E-10 | 0.63 |
| Gain | cg17321954 | 3 | 120626881 |  | Island | NM_014980 | TSS200 | STXBP5L | 3.64E-16 | 1.59E-10 | 0.65 |
| Gain | cg19399220 | 19 | 10527588 |  | Island |  |  |  | 3.74E-16 | 1.64E-10 | 0.65 |
| Gain | cg19273773 | 7 | 102790112 |  | Island | NM_198990;NM_001122838 | TSS1500;TSS1500 | NAPEPLD;NAPEPLD | 4.12E-16 | 1.80E-10 | 0.65 |
| Gain | cg00439658 | 17 | 72848669 | TRUE | Island | NM_000835 | Body | GRIN2C | 4.57E-16 | 2.00E-10 | 0.63 |
| Gain | cg06335143 | 1 | 53308654 |  | Island | NM_001004339 | Body | ZYG11A | 4.73E-16 | 2.07E-10 | 0.64 |
| Gain | cg00065935 | 3 | 120627088 |  | Island | NM_014980;NM_014980 | 1stExon;5'UTR | STXBP5L;STXBP5L | 5.68E-16 | 2.49E-10 | 0.64 |
| Gain | cg21186299 | 7 | 100808810 |  | Island | NM_003378;NM_003378 | 1stExon;5'UTR | VGF;VGF | 6.73E-16 | 2.95E-10 | 0.65 |
| Gain | cg03020208 | 12 | 50354962 |  | Island | NM_001651 | TSS1500 | AQP5 | 7.23E-16 | 3.17E-10 | 0.62 |
| Gain | cg25287474 | 9 | 14693538 |  | Island | NM_178566 | TSS200 | ZDHHC21 | 7.55E-16 | 3.30E-10 | 0.65 |
| Gain | cg15355387 | 3 | 85007817 |  | N_Shore | NM_001167674;NM_001167675 | TSS1500;TSS1500 | CADM2;CADM2 | 8.79E-16 | 3.85E-10 | 0.64 |
| Gain | cg09977376 | 1 | 53951348 | TRUE |  |  |  |  | 9.67E-16 | 4.23E-10 | 0.63 |
| Gain | cg16489193 | 6 | 33240059 |  | Island | NM_022553;NM_022551 | TSS1500;Body | VPS52;RPS18 | 1.02E-15 | 4.48E-10 | 0.64 |
| Gain | cg12226046 | 10 | 91403655 |  | N_Shore | NM_148977;NM_148978;NM_138316 | Body;TSS200;TSS200 | PANK1;PANK1;PANK1 | 1.04E-15 | 4.56E-10 | 0.63 |
| Gain | cg20121753 | 7 | 37221742 | TRUE |  | NM_014800 | Body | ELMO1 | 1.10E-15 | 4.81E-10 | 0.62 |
| Gain | cg03555227 | 5 | 170289070 |  | Island | NM_022897 | Body | RANBP17 | 1.15E-15 | 5.04E-10 | 0.64 |
| Gain | cg01637011 | 1 | 200992964 |  | S_Shore | NM_017596 | TSS200 | KIF21B | 1.16E-15 | 5.09E-10 | 0.64 |
| Gain | cg04271792 | 6 | 28831393 |  | N_Shore |  |  |  | 1.29E-15 | 5.62E-10 | 0.63 |
| Gain | cg04768203 | 11 | 66189188 |  | Island | NM_178864 | Body | NPAS4 | 1.53E-15 | 6.69E-10 | 0.64 |
| Gain | cg01429039 | 4 | 52918065 |  | Island | NM_145263 | Body | SPATA18 | 1.54E-15 | 6.74E-10 | 0.64 |
| Gain | cg23654174 | 1 | 47779667 |  | N_Shore | NM_003035;NM_001048166 | 5'UTR;5'UTR | STIL;STIL | 1.60E-15 | 7.01E-10 | 0.63 |
| Gain | cg14241323 | 18 | 13824002 |  | Island |  |  |  | 1.64E-15 | 7.17E-10 | 0.62 |
| Gain | cg00590036 | 6 | 158957433 |  | Island | NM_020823 | TSS200 | TMEM181 | 1.80E-15 | 7.86E-10 | 0.63 |
| Gain | cg25148589 | 4 | 158141936 |  | N_Shore | NM_001083619;NM_000826;NM_001083620;NM_000826;NM_001083619 | 1stExon;5'UTR;5'UTR;1stExon;5'UTR | GRIA2;GRIA2;GRIA2;GRIA2;GRIA2 | 1.87E-15 | 8.17E-10 | 0.62 |
| Gain | cg04782470 | 19 | 18208422 |  | N_Shore | NM_015016 | TSS200 | MAST3 | 2.45E-15 | 1.07E-09 | 0.64 |
| Gain | cg19451698 | 2 | 227700467 |  | Island | NM_001167608;NM_032276 | TSS1500;TSS1500 | RHBDD1;RHBDD1 | 2.55E-15 | 1.12E-09 | 0.63 |
| Gain | cg03607117 | 3 | 53080440 |  | Island | NM_001005159;NM_016329;NM_001005158 | TSS1500;TSS1500;TSS1500 | SFMBT1;SFMBT1;SFMBT1 | 2.56E-15 | 1.12E-09 | 0.63 |
| Gain | cg04241863 | 8 | 18067331 | TRUE |  | NM_001160173;NM_001160172;NM_001160176;NM_001160175;NM_001160170;NM_001160171;NM_000662 | TSS1500;TSS1500;TSS1500;TSS1500;TSS1500;TSS1500;TSS1500;5'UTR | NAT1;NAT1;NAT1;NAT1;NAT1;NAT1;NAT1;NAT1 | 2.70E-15 | 1.18E-09 | 0.63 |
| Gain | cg04008429 | 3 | 160167646 |  | Island | NM_173084 | TSS200 | TRIM59 | 2.92E-15 | 1.28E-09 | 0.63 |
| Gain | cg24214260 | 19 | 58193221 |  | N_Shore | NM_138347 | TSS200 | ZNF551 | 2.93E-15 | 1.28E-09 | 0.63 |
| Gain | cg26856080 | 3 | 160167746 |  | Island | NM_173084 | TSS200 | TRIM59 | 3.17E-15 | 1.39E-09 | 0.64 |
| Gain | cg00489219 | 3 | 40566141 |  | Island | NM_198484;NM_001098414 | TSS1500;TSS1500 | ZNF621;ZNF621 | 3.36E-15 | 1.47E-09 | 0.63 |
| Gain | cg11082362 | 14 | 36003181 |  | Island | NM_032594 | TSS200 | INSM2 | 3.39E-15 | 1.48E-09 | 0.62 |
| Gain | cg20119148 | 19 | 18344195 |  | Island | NM_000923 | 5'UTR | PDE4C | 3.63E-15 | 1.59E-09 | 0.64 |
| Gain | cg02489908 | 17 | 6946086 |  | Island | NM_153357 | Body | SLC16A11 | 3.64E-15 | 1.59E-09 | 0.64 |
| Gain | cg15313320 | 11 | 17374127 |  | N_Shore | NR_026750 | Body | DKFZp686O24166 | 4.08E-15 | 1.78E-09 | 0.64 |
| Gain | cg19055803 | 19 | 12947164 |  | N_Shore | NM_031429 | TSS1500 | RTBDN | 4.47E-15 | 1.96E-09 | 0.63 |
| Gain | cg21182694 | 3 | 139062771 |  | N_Shore | NM_020191 | TSS200 | MRPS22 | 5.12E-15 | 2.24E-09 | 0.63 |
| Gain | cg05261559 | 13 | 20702922 |  | Island |  |  |  | 5.33E-15 | 2.33E-09 | 0.63 |
| Gain | cg26161329 | 17 | 56832991 |  | Island | NM_014906 | TSS1500 | PPM1E | 5.46E-15 | 2.39E-09 | 0.62 |
| Gain | cg19897172 | 7 | 135347071 |  | Island | NM_001130929 | TSS200 | PL-5283 | 7.06E-15 | 3.09E-09 | 0.63 |
| Gain | cg09012544 | 2 | 71503562 |  | Island |  |  |  | 7.31E-15 | 3.20E-09 | 0.58 |
| Gain | cg11896587 | 19 | 39440734 |  | Island | NM_024907;NM_148169 | Body;Body | FBXO17;FBXO17 | 7.49E-15 | 3.28E-09 | 0.62 |
| Gain | cg09997676 | 13 | 50070482 |  | Island | NM_001040444;NM_001040443 | TSS200;Body | PHF11;PHF11 | 7.52E-15 | 3.29E-09 | 0.61 |
| Gain | cg25246431 | 11 | 12398874 |  | N_Shore | NM_018222 | TSS1500 | PARVA | 9.09E-15 | 3.98E-09 | 0.63 |
| Gain | cg01644850 | 19 | 58193231 |  | N_Shore | NM_138347 | TSS200 | ZNF551 | 9.11E-15 | 3.98E-09 | 0.63 |
| Gain | cg00840310 | 9 | 74061514 |  | Island |  |  |  | 9.14E-15 | 4.00E-09 | 0.62 |
| Gain | cg22310062 | 18 | 19181025 |  | Island | NM_052911 | TSS1500 | ESCO1 | 9.20E-15 | 4.03E-09 | 0.61 |
| Gain | cg17243289 | 18 | 45458021 |  | Island | NM_005901;NM_001135937;NM_001003652 | TSS1500;TSS1500;TSS1500 | SMAD2;SMAD2;SMAD2 | 1.35E-14 | 5.91E-09 | 0.62 |
| Gain | cg11071401 | 17 | 48637194 | TRUE | Island | NM_198384;NM_198397;NM_198396;NM_198379;NM_198383;NM_198380;NM_198387;NM_198378;NM_1983 | TSS1500;TSS1500;TSS1500;TSS1500;TSS1500;TSS1500;TSS1500;TSS1500;TSS1500;TSS1500;TSS1500 | CACNA1G;CACNA1G;CACNA1G;CACNA1G;CACNA1G;CACNA1G;CACNA1G;CACNA1G;CACNA1G;CACNA1G;CACNA1G | 1.43E-14 | 6.24E-09 | 0.61 |
| Gain | cg14311320 | 7 | 124405732 |  | Island | NM_005302 | TSS200 | GPR37 | 1.81E-14 | 7.93E-09 | 0.61 |
| Gain | cg10699064 | 5 | 122110553 |  | Island | NM_003100 | TSS200 | SNX2 | 1.88E-14 | 8.22E-09 | 0.62 |
| Gain | cg07584066 | 17 | 57642749 |  | Island | NM_001166301;NM_024612 | TSS200;TSS200 | DHX40;DHX40 | 1.88E-14 | 8.25E-09 | 0.62 |
| Gain | cg24393316 | 9 | 100616469 |  | Island | NM_004473 | 1stExon | FOXE1 | 1.89E-14 | 8.28E-09 | 0.63 |
| Gain | cg21956434 | 19 | 17377697 |  |  | NM_001033549;NM_014173 | TSS1500;TSS1500 | C19orf62;C19orf62 | 1.91E-14 | 8.34E-09 | 0.62 |
| Gain | cg11752769 | 1 | 207818423 |  | Island | NM_175710 | TSS200 | CR1L | 2.25E-14 | 9.85E-09 | 0.62 |
| Gain | cg08377768 | 3 | 160474222 |  | Island | NM_139245 | 1stExon | PPM1L | 2.32E-14 | 1.01E-08 | 0.62 |
| Gain | cg14692377 | 17 | 28562685 |  | Island | NM_001045;NM_001045 | 1stExon;5'UTR | SLC6A4;SLC6A4 | 2.38E-14 | 1.04E-08 | 0.61 |
| Gain | cg08169949 | 1 | 28241532 |  | Island | NM_002946 | TSS1500 | RPA2 | 2.72E-14 | 1.19E-08 | 0.62 |
| Gain | cg22417733 | 6 | 153303409 |  | Island | NM_001142522;NM_012177 | 5'UTR;Body | FBXO5;FBXO5 | 2.78E-14 | 1.22E-08 | 0.60 |
| Gain | cg27541691 | 17 | 40811050 |  | N_Shore | NM_016437 | TSS1500 | TUBG2 | 2.94E-14 | 1.29E-08 | 0.61 |
| Gain | cg10341152 | 13 | 37393069 |  | N_Shore | NM_000538 | TSS1500 | RFXAP | 3.16E-14 | 1.38E-08 | 0.62 |
| Gain | cg01214340 | 13 | 50070499 |  | Island | NM_001040444;NM_001040443 | TSS200;Body | PHF11;PHF11 | 3.28E-14 | 1.44E-08 | 0.61 |
| Gain | cg04268670 | 5 | 76926341 | TRUE | Island | NM_032109 | Body | OTP | 3.43E-14 | 1.50E-08 | 0.61 |
| Gain | cg10548492 | 7 | 96746798 |  | Island | NM_020186;NM_020186 | 1stExon;5'UTR | ACN9;ACN9 | 3.50E-14 | 1.53E-08 | 0.62 |
| Gain | cg12204897 | 9 | 131486753 |  | Island | NM_032799 | TSS1500 | ZDHHC12 | 3.86E-14 | 1.69E-08 | 0.62 |
| Gain | cg23998119 | 14 | 77600217 |  |  | NM_174976 | Body | ZDHHC22 | 4.01E-14 | 1.76E-08 | 0.60 |
| Gain | cg08482682 | 11 | 107799197 | TRUE | Island | NM_017516 | TSS200 | RAB39 | 4.11E-14 | 1.80E-08 | 0.61 |
| Gain | cg25622481 | 2 | 73518365 |  | Island | NM_001965 | 3'UTR | EGR4 | 4.19E-14 | 1.83E-08 | 0.61 |
| Gain | cg26734668 | 19 | 58111094 |  | N_Shore | NM_020880 | TSS200 | ZNF530 | 4.55E-14 | 1.99E-08 | 0.61 |
| Gain | cg25478614 | 3 | 187387866 | TRUE | N_Shore | NM_001048 | Body | SST | 4.95E-14 | 2.17E-08 | 0.59 |
| Gain | cg10947146 | 8 | 11058710 |  | Island | NM_173683 | 1stExon | XKR6 | 5.35E-14 | 2.34E-08 | 0.61 |
| Gain | cg26956371 | 10 | 124713989 |  | Island | NM_024942 | TSS200 | C10orf88 | 5.90E-14 | 2.58E-08 | 0.61 |
| Gain | cg18240400 | 10 | 46168597 |  | Island | NM_001128324;NM_174890 | TSS1500;TSS1500 | ANUBL1;ANUBL1 | 5.96E-14 | 2.61E-08 | 0.61 |
| Gain | cg12934382 | 3 | 51741135 | TRUE | Island | NM_001130063;NM_000839;NM_000839;NM_001130063 | 1stExon;5'UTR;1stExon;5'UTR | GRM2;GRM2;GRM2;GRM2 | 6.90E-14 | 3.02E-08 | 0.60 |
| Gain | cg14112635 | 17 | 76250769 |  | Island |  |  |  | 7.14E-14 | 3.12E-08 | 0.61 |
| Gain | cg17009433 | 9 | 6645686 | TRUE | Island | NM_000170;NM_000170 | 5'UTR;1stExon | GLDC;GLDC | 7.18E-14 | 3.14E-08 | 0.61 |
| Gain | cg24038454 | 14 | 36003449 |  | Island | NM_032594;NM_032594 | 5'UTR;1stExon | INSM2;INSM2 | 8.13E-14 | 3.56E-08 | 0.61 |
| Gain | cg02026306 | 19 | 50184053 |  | Island | NM_198318;NM_198319;NM_001536 | Body;Body;Body | PRMT1;PRMT1;PRMT1 | 8.52E-14 | 3.73E-08 | 0.60 |
| Gain | cg08770761 | 16 | 2563461 |  | Island | NM_001694 | TSS1500 | ATP6V0C | 9.54E-14 | 4.18E-08 | 0.61 |
| Gain | cg10284662 | 16 | 89008451 |  | Island | NM_175931;NM_005187 | TSS1500;Body | CBFA2T3;CBFA2T3 | 9.79E-14 | 4.28E-08 | 0.60 |
| Gain | cg11299854 | 5 | 132083184 |  | Island | NM_001039780;NM_001039780 | 1stExon;5'UTR | CCNI2;CCNI2 | 1.03E-13 | 4.53E-08 | 0.61 |
| Gain | cg08318076 | 8 | 62051812 | TRUE | Island |  |  |  | 1.07E-13 | 4.68E-08 | 0.60 |
| Gain | cg18633600 | 12 | 1940452 | TRUE |  | NM_001163925;NM_001039029;NM_172364;NM_001163926 | Body;Body;Body;Body | LRTM2;LRTM2;CACNA2D4;LRTM2 | 1.10E-13 | 4.83E-08 | 0.60 |
| Gain | cg23517605 | 6 | 3228365 |  | Island | NM_178012 | TSS1500 | TUBB2B | 1.18E-13 | 5.15E-08 | 0.60 |
| Gain | cg16778903 | 15 | 72612567 | TRUE | Island | NM_052840 | TSS200 | BRUNOL6 | 1.20E-13 | 5.24E-08 | 0.59 |
| Gain | cg16026922 | 4 | 41259044 |  | Island | NM_004181 | Body | UCHL1 | 1.28E-13 | 5.62E-08 | 0.60 |
| Gain | cg24497836 | 15 | 48470678 |  | S_Shore | NM_016132 | TSS200 | MYEF2 | 1.30E-13 | 5.67E-08 | 0.61 |
| Gain | cg04212239 | 3 | 160119314 |  | S_Shore | NM_005496;NM_001002800 | Body;Body | SMC4;SMC4 | 1.36E-13 | 5.97E-08 | 0.61 |
| Gain | cg26129417 | 5 | 134527034 |  | Island |  |  |  | 1.37E-13 | 6.01E-08 | 0.60 |
| Gain | cg24466241 | 1 | 53308908 |  | Island | NM_001004339 | Body | ZYG11A | 1.50E-13 | 6.57E-08 | 0.59 |
| Gain | cg09593028 | 10 | 97890481 |  | S_Shore | NM_014803 | 5'UTR | ZNF518A | 1.64E-13 | 7.19E-08 | 0.58 |
| Gain | cg14848772 | 6 | 27099813 |  | N_Shore | NM_021064 | TSS1500 | HIST1H2AG | 1.86E-13 | 8.14E-08 | 0.60 |
| Gain | cg25569840 | 22 | 49701234 |  | S_Shelf |  |  |  | 1.86E-13 | 8.14E-08 | 0.59 |
| Gain | cg00068155 | 3 | 131754131 |  | Island | NM_130808 | TSS1500 | CPNE4 | 1.96E-13 | 8.60E-08 | 0.59 |
| Gain | cg14361627 | 7 | 130419116 |  | Island | NM_138693 | TSS1500 | KLF14 | 1.96E-13 | 8.60E-08 | 0.59 |
| Gain | cg25047092 | 2 | 61991905 |  | S_Shore |  |  |  | 1.97E-13 | 8.63E-08 | 0.60 |
| Gain | cg21845273 | 3 | 44037001 |  | Island |  |  |  | 2.00E-13 | 8.74E-08 | 0.61 |
| Gain | cg08114812 | 1 | 41445105 |  | Island | NM_001905;NM_001905 | 1stExon;5'UTR | CTPS;CTPS | 2.05E-13 | 8.97E-08 | 0.60 |
| Gain | cg02018902 | 15 | 79576149 |  | Island | NM_001146341 | 5'UTR | ANKRD34C | 2.05E-13 | 8.99E-08 | 0.60 |
| Gain | cg23158811 | 19 | 19729732 | TRUE | Island | NM_025245 | TSS1500 | PBX4 | 2.10E-13 | 9.17E-08 | 0.60 |
| Gain | cg00888561 | 13 | 52157910 |  | N_Shore | NM_052950 | TSS1500 | WDFY2 | 2.15E-13 | 9.42E-08 | 0.60 |
| Gain | cg06034933 | 1 | 228645634 |  | Island | NM_033445;NM_175055 | TSS200;TSS200 | HIST3H2A;HIST3H2BB | 2.15E-13 | 9.43E-08 | 0.60 |
| Gain | cg06648759 | 13 | 40892751 | TRUE |  |  |  |  | 2.19E-13 | 9.60E-08 | 0.60 |
| Gain | cg07920503 | 13 | 25745406 | TRUE | Island | NM_199138;NM_152704 | 1stExon;1stExon | FAM123A;FAM123A | 2.21E-13 | 9.66E-08 | 0.60 |
| Gain | cg22682373 | 12 | 22697774 |  | S_Shore | NM_014802 | TSS1500 | KIAA0528 | 2.24E-13 | 9.81E-08 | 0.59 |
| Gain | cg23091758 | 11 | 9025767 |  | Island | NM_020645 | TSS200 | NRIP3 | 2.26E-13 | 9.88E-08 | 0.61 |
| Gain | cg04368796 | 17 | 40171968 |  | Island | NM_001144927;NM_001144929;NM_001144928;NM_017595;NM_001001349 | TSS200;TSS200;TSS200;TSS200;5'UTR | NKIRAS2;NKIRAS2;NKIRAS2;NKIRAS2;NKIRAS2 | 2.41E-13 | 1.05E-07 | 0.60 |
| Gain | cg06493994 | 6 | 25652602 |  | Island | NM_006998;NM_006998 | 1stExon;5'UTR | SCGN;SCGN | 2.50E-13 | 1.09E-07 | 0.60 |
| Gain | cg21877855 | 1 | 63989125 |  |  | NM_014288;NM_032437;NM_032437 | TSS1500;1stExon;5'UTR | ITGB3BP;EFCAB7;EFCAB7 | 2.51E-13 | 1.10E-07 | 0.60 |
| Gain | cg07873590 | 19 | 17858298 |  | N_Shore | NM_001161358;NM_001161357 | TSS1500;TSS1500 | FCHO1;FCHO1 | 2.64E-13 | 1.16E-07 | 0.58 |
| Gain | cg24847541 | 13 | 92051154 |  | Island | NM_004466;NM_004466 | 5'UTR;1stExon | GPC5;GPC5 | 2.75E-13 | 1.20E-07 | 0.59 |
| Gain | cg09784307 | 15 | 75248768 |  | Island | NM_017793 | 1stExon | RPP25 | 2.96E-13 | 1.29E-07 | 0.58 |
| Gain | cg08748615 | 1 | 39957298 |  | Island | NM_181809 | TSS200 | BMP8A | 3.56E-13 | 1.56E-07 | 0.59 |
| Gain | cg12018403 | 19 | 55677414 | TRUE | N_Shore | NM_178837 | Body | C19orf51 | 3.80E-13 | 1.66E-07 | 0.59 |
| Gain | cg06144905 | 17 | 27369780 |  |  | NM_016518 | TSS200 | PIPOX | 3.82E-13 | 1.67E-07 | 0.57 |
| Gain | cg23197992 | 5 | 176514067 |  | Island | NM_213647;NM_002011;NM_213647 | 1stExon;5'UTR;5'UTR | FGFR4;FGFR4;FGFR4 | 4.16E-13 | 1.82E-07 | 0.59 |
| Gain | cg02355885 | 2 | 30142990 |  | Island | NM_004304 | 1stExon | ALK | 4.36E-13 | 1.91E-07 | 0.60 |
| Gain | cg24408436 | 11 | 64739374 |  | Island |  |  |  | 4.39E-13 | 1.92E-07 | 0.59 |
| Gain | cg00745389 | 7 | 32467435 | TRUE | Island |  |  |  | 4.69E-13 | 2.05E-07 | 0.58 |
| Gain | cg13575161 | 12 | 4381792 |  | Island | NM_001759 | TSS1500 | CCND2 | 5.00E-13 | 2.19E-07 | 0.60 |
| Gain | cg07112260 | 16 | 67034573 | TRUE | Island | NM_173815 | Body | CES8 | 5.05E-13 | 2.21E-07 | 0.58 |
| Gain | cg03314644 | 14 | 31890001 |  | Island |  |  |  | 5.06E-13 | 2.21E-07 | 0.59 |
| Gain | cg24290286 | 11 | 17374112 |  | N_Shore | NR_026750 | Body | DKFZp686O24166 | 5.52E-13 | 2.41E-07 | 0.59 |
| Gain | cg16738971 | 5 | 125931166 |  | S_Shore | NM_001182 | TSS200 | ALDH7A1 | 5.53E-13 | 2.42E-07 | 0.58 |
| Gain | cg13948585 | 1 | 181451906 |  | Island | NM_000721 | TSS1500 | CACNA1E | 5.55E-13 | 2.43E-07 | 0.60 |
| Gain | cg23995914 | 4 | 10459228 |  | Island | NM_053042 | TSS200 | ZNF518B | 5.95E-13 | 2.60E-07 | 0.60 |
| Gain | cg13649056 | 9 | 136474626 |  | Island |  |  |  | 6.09E-13 | 2.66E-07 | 0.58 |
| Gain | cg04834794 | 3 | 128400154 |  | Island |  |  |  | 6.24E-13 | 2.73E-07 | 0.59 |
| Gain | cg20315346 | 15 | 41523051 |  | Island | NM_007236;NM_152596 | TSS1500;TSS200 | CHP;EXD1 | 6.37E-13 | 2.79E-07 | 0.59 |
| Gain | cg10523019 | 2 | 227700458 |  | Island | NM_001167608;NM_032276 | TSS1500;TSS1500 | RHBDD1;RHBDD1 | 6.78E-13 | 2.97E-07 | 0.58 |
| Gain | cg02681442 | 14 | 29236008 |  | N_Shore | NM_005249 | TSS1500 | FOXG1 | 6.83E-13 | 2.99E-07 | 0.60 |
| Gain | cg21282131 | 6 | 136871907 |  | Island | NM_003980 | TSS200 | MAP7 | 6.98E-13 | 3.06E-07 | 0.59 |
| Gain | cg23718606 | 6 | 27219812 |  | Island | NM_005865 | Body | PRSS16 | 7.03E-13 | 3.08E-07 | 0.59 |
| Gain | cg26974111 | 18 | 12254120 |  | N_Shore | NM_198289;NM_001279 | TSS1500;TSS200 | CIDEA;CIDEA | 7.04E-13 | 3.08E-07 | 0.59 |
| Gain | cg07846081 | 3 | 131754018 |  | Island | NM_130808 | TSS200 | CPNE4 | 8.10E-13 | 3.55E-07 | 0.58 |
| Gain | cg03350900 | 6 | 107955689 |  | Island | NM_018013 | Body | SOBP | 8.38E-13 | 3.67E-07 | 0.60 |
| Gain | cg07477282 | 15 | 44956107 |  | S_Shore | NM_025137;NM_001160227 | TSS1500;TSS1500 | SPG11;SPG11 | 8.68E-13 | 3.80E-07 | 0.59 |
| Gain | cg18213931 | 6 | 35995246 |  | Island | NM_139013;NM_001315;NM_139014;NM_139012 | TSS1500;TSS1500;TSS1500;TSS1500 | MAPK14;MAPK14;MAPK14;MAPK14 | 9.00E-13 | 3.94E-07 | 0.58 |
| Gain | cg23193410 | 5 | 172385677 |  | Island | NR_026682;NR_026683;NM_016093 | Body;Body;TSS1500 | LOC100268168;LOC100268168;RPL26L1 | 9.49E-13 | 4.15E-07 | 0.54 |
| Gain | cg25105633 | 14 | 105480568 | TRUE |  | NM_017955;NM_145701 | 5'UTR;5'UTR | CDCA4;CDCA4 | 1.01E-12 | 4.40E-07 | 0.59 |
| Gain | cg07392449 | 8 | 11324666 |  | Island | NM_053279 | TSS1500 | FAM167A | 1.12E-12 | 4.92E-07 | 0.59 |
| Gain | cg14686745 | 6 | 47756010 |  |  | NM_001030051;NM_181744 | 5'UTR;Body | OPN5;OPN5 | 1.13E-12 | 4.96E-07 | 0.58 |
| Gain | cg18549036 | 3 | 148804541 |  | Island | NM_139048;NM_003071 | TSS1500;TSS1500 | HLTF;HLTF | 1.24E-12 | 5.44E-07 | 0.57 |
| Gain | cg10820926 | 14 | 30397408 |  | Island | NM_002742 | TSS1500 | PRKD1 | 1.32E-12 | 5.79E-07 | 0.59 |
| Gain | cg01947224 | 19 | 58089885 |  | Island | NM_017879 | Body | ZNF416 | 1.46E-12 | 6.38E-07 | 0.58 |
| Gain | cg07547549 | 20 | 44658225 |  | Island | NM_020708;NM_001134771 | Body;Body | SLC12A5;SLC12A5 | 1.54E-12 | 6.75E-07 | 0.58 |
| Gain | cg11197101 | 1 | 33219998 | TRUE | Island | NM_020888 | Body | KIAA1522 | 1.59E-12 | 6.95E-07 | 0.58 |
| Gain | cg08461586 | 10 | 97802977 |  | Island | NM_001134376;NM_019084;NM_001134375 | TSS200;TSS200;TSS200 | CCNJ;CCNJ;CCNJ | 1.65E-12 | 7.22E-07 | 0.58 |
| Gain | cg04878973 | 3 | 49057761 |  | N_Shore | NM_199074;NM_018114;NR_029948;NM_199070;NM_199069;NM_199073 | TSS200;5'UTR;TSS200;TSS1500;TSS1500;TSS1500 | NDUFAF3;DALRD3;MIR425;NDUFAF3;NDUFAF3;NDUFAF3 | 1.67E-12 | 7.30E-07 | 0.58 |
| Gain | cg20263901 | 6 | 41438593 |  | Island |  |  |  | 1.75E-12 | 7.67E-07 | 0.58 |
| Gain | cg26475688 | 13 | 20702970 |  | Island |  |  |  | 1.95E-12 | 8.51E-07 | 0.58 |
| Gain | cg08541521 | 1 | 111506029 |  | Island | NM_018372;NM_001006945 | Body;5'UTR | C1orf103;C1orf103 | 2.08E-12 | 9.11E-07 | 0.57 |
| Gain | cg16909962 | 1 | 229406711 |  | Island | NM_004578 | TSS200 | RAB4A | 2.10E-12 | 9.17E-07 | 0.57 |
| Gain | cg23684204 | 15 | 91497937 |  | N_Shore | NM_033544;NM_001017919 | TSS200;TSS200 | RCCD1;RCCD1 | 2.13E-12 | 9.33E-07 | 0.58 |
| Gain | cg02071825 | 17 | 56833096 |  | Island | NM_014906 | TSS200 | PPM1E | 2.14E-12 | 9.38E-07 | 0.57 |
| Gain | cg01844642 | 3 | 51989764 | TRUE | Island | NM_080865 | 1stExon | GPR62 | 2.26E-12 | 9.88E-07 | 0.57 |
| Gain | cg05555455 | 3 | 148804550 |  | Island | NM_139048;NM_003071 | TSS1500;TSS1500 | HLTF;HLTF | 2.31E-12 | 1.01E-06 | 0.57 |
| Gain | cg08147050 | 11 | 93394607 |  | N_Shore | NM_033395 | TSS1500 | KIAA1731 | 2.32E-12 | 1.02E-06 | 0.58 |
| Gain | cg08622677 | 12 | 3601306 |  | Island | NM_019854 | Body | PRMT8 | 2.46E-12 | 1.08E-06 | 0.58 |
| Gain | cg07189157 | 7 | 149119407 |  | Island |  |  |  | 2.59E-12 | 1.13E-06 | 0.57 |
| Gain | cg01592801 | 8 | 99438942 | TRUE | Island | NM_020697 | TSS1500 | KCNS2 | 2.69E-12 | 1.18E-06 | 0.56 |
| Gain | cg14611683 | 1 | 45452580 |  | S_Shore | NM_020365;NM_001166588 | TSS1500;TSS1500 | EIF2B3;EIF2B3 | 2.77E-12 | 1.21E-06 | 0.56 |
| Gain | cg10254690 | 10 | 126107861 |  | Island | NM_000274 | TSS1500 | OAT | 2.87E-12 | 1.26E-06 | 0.56 |
| Gain | cg13830081 | 6 | 10404363 |  | Island | NM_001042425;NM_001032280;NM_003220 | Body;Body;Body | TFAP2A;TFAP2A;TFAP2A | 2.99E-12 | 1.31E-06 | 0.57 |
| Gain | cg21477033 | 5 | 111496357 |  | N_Shore | NR_015370;NR_002922 | Body;TSS1500 | NCRNA00219;SNORA13 | 3.07E-12 | 1.35E-06 | 0.57 |
| Gain | cg01667837 | 7 | 124405605 |  | Island | NM_005302;NM_005302 | 1stExon;5'UTR | GPR37;GPR37 | 3.49E-12 | 1.53E-06 | 0.58 |
| Gain | cg14261272 | 2 | 127976400 |  | Island |  |  |  | 3.68E-12 | 1.61E-06 | 0.57 |
| Gain | cg23756251 | 3 | 179754521 | TRUE | Island | NM_016559 | TSS200 | PEX5L | 4.06E-12 | 1.78E-06 | 0.55 |
| Gain | cg06073351 | 10 | 22625665 |  | Island |  |  |  | 4.17E-12 | 1.83E-06 | 0.58 |
| Gain | cg00593900 | 19 | 10206746 | TRUE | Island | NM_031917 | Body | ANGPTL6 | 4.26E-12 | 1.86E-06 | 0.57 |
| Gain | cg13682722 | 14 | 90798568 | TRUE | S_Shore | NM_199043;NM_017970 | TSS1500;TSS1500 | C14orf102;C14orf102 | 4.57E-12 | 2.00E-06 | 0.57 |
| Gain | cg21449170 | 7 | 130419062 |  | Island | NM_138693 | TSS200 | KLF14 | 4.71E-12 | 2.06E-06 | 0.57 |
| Gain | cg18787012 | 11 | 15230314 |  |  | NM_001031853;NM_001042536 | Body;Body | INSC;INSC | 5.03E-12 | 2.20E-06 | 0.56 |
| Gain | cg13816999 | 11 | 12398883 |  | N_Shore | NM_018222 | TSS1500 | PARVA | 5.14E-12 | 2.25E-06 | 0.58 |
| Gain | cg21424782 | 20 | 48599531 |  | Island | NM_005985;NM_005985 | 5'UTR;1stExon | SNAI1;SNAI1 | 5.26E-12 | 2.30E-06 | 0.57 |
| Gain | cg09058748 | 2 | 87035698 | TRUE | N_Shore | NM_001145873 | TSS200 | CD8A | 5.32E-12 | 2.33E-06 | 0.57 |
| Gain | cg00481951 | 3 | 187387650 | TRUE | N_Shore | NM_001048 | Body | SST | 5.53E-12 | 2.42E-06 | 0.57 |
| Gain | cg24338780 | 1 | 174968123 |  | N_Shore | NM_014412;NM_001007214 | TSS1500;TSS1500 | CACYBP;CACYBP | 5.55E-12 | 2.43E-06 | 0.58 |
| Gain | cg07172885 | 12 | 133757907 |  | N_Shore | NM_001165886;NM_001165885;NM_001165887;NM_001165882;NM_001165883;NM_003415;NM_001165884 | TSS200;TSS200;TSS200;TSS200;TSS200;TSS200;TSS200;TSS200;TSS200 | ZNF268;ZNF268;ZNF268;ZNF268;ZNF268;ZNF268;ZNF268;ZNF268;ZNF268 | 5.57E-12 | 2.44E-06 | 0.56 |
| Gain | cg26417361 | 6 | 99292838 |  | Island |  |  |  | 5.68E-12 | 2.49E-06 | 0.57 |
| Gain | cg19688118 | 5 | 114632615 |  | S_Shore | NM_152549;NM_001040440 | TSS1500;TSS200 | CCDC112;CCDC112 | 5.81E-12 | 2.54E-06 | 0.57 |
| Gain | cg24724428 | 6 | 11044888 |  | Island | NM_017770 | TSS1500 | ELOVL2 | 5.94E-12 | 2.60E-06 | 0.57 |
| Gain | cg08785215 | 3 | 57993979 |  | Island | NM_001164318;NM_001164317;NM_001457;NM_001164319 | TSS200;TSS200;TSS200;TSS200 | FLNB;FLNB;FLNB;FLNB | 6.02E-12 | 2.63E-06 | 0.56 |
| Gain | cg05668372 | 20 | 62734251 |  | Island |  |  |  | 6.10E-12 | 2.67E-06 | 0.57 |
| Gain | cg00338080 | 7 | 75889659 |  | Island | NM_001110199 | Body | SRRM3 | 6.33E-12 | 2.77E-06 | 0.57 |
| Gain | cg26005082 | 19 | 4769660 |  |  | NR_029607;NR_027148 | TSS1500;Body | MIR7-3;C19orf30 | 6.80E-12 | 2.97E-06 | 0.57 |
| Gain | cg16969368 | 17 | 57642752 |  | Island | NM_001166301;NM_024612 | TSS200;TSS200 | DHX40;DHX40 | 6.92E-12 | 3.03E-06 | 0.58 |
| Gain | cg13202816 | 11 | 32605193 |  | Island | NM_006360 | TSS200 | EIF3M | 7.74E-12 | 3.39E-06 | 0.56 |
| Gain | cg05906092 | 7 | 140098427 |  | Island | NM_207113;NM_032295 | TSS200;TSS200 | SLC37A3;SLC37A3 | 8.13E-12 | 3.56E-06 | 0.56 |
| Gain | cg09190280 | 4 | 2043414 |  | Island | NM_001168243;NM_001141936 | TSS1500;TSS1500 | C4orf48;C4orf48 | 8.55E-12 | 3.74E-06 | 0.56 |
| Gain | cg10469100 | 3 | 44690127 |  | Island | NM_003420 | TSS200 | ZNF35 | 8.76E-12 | 3.84E-06 | 0.56 |
| Gain | cg16113692 | 11 | 22851502 |  | S_Shore | NM_148893 | TSS200 | SVIP | 9.07E-12 | 3.97E-06 | 0.55 |
| Gain | cg06879152 | 10 | 60937041 |  | Island | NM_032439;NM_001143774 | Body;TSS200 | PHYHIPL;PHYHIPL | 9.14E-12 | 4.00E-06 | 0.56 |
| Gain | cg21159778 | 9 | 117266918 |  | Island | NM_001083885;NM_015404 | TSS1500;1stExon | DFNB31;DFNB31 | 9.73E-12 | 4.26E-06 | 0.56 |
| Gain | cg09136245 | 11 | 20631761 | TRUE |  | NM_004211 | Body | SLC6A5 | 1.13E-11 | 4.95E-06 | 0.56 |
| Gain | cg15906794 | 17 | 7197963 |  | Island | NM_015982 | TSS200 | YBX2 | 1.23E-11 | 5.40E-06 | 0.56 |
| Gain | cg12845952 | 3 | 167098119 |  | Island | NM_024687 | TSS200 | ZBBX | 1.24E-11 | 5.43E-06 | 0.56 |
| Gain | cg23045594 | 2 | 71503883 |  | Island |  |  |  | 1.29E-11 | 5.65E-06 | 0.55 |
| Gain | cg09597312 | 17 | 21360346 |  | S_Shelf |  |  |  | 1.40E-11 | 6.14E-06 | 0.55 |
| Gain | cg23582919 | 16 | 23568380 |  | N_Shore | NM_019116;NR_003501;NM_001083614 | TSS1500;Body;Body | UBFD1;EARS2;EARS2 | 1.45E-11 | 6.34E-06 | 0.55 |
| Gain | cg15623892 | 5 | 122110559 |  | Island | NM_003100 | TSS200 | SNX2 | 1.47E-11 | 6.41E-06 | 0.56 |
| Gain | cg03943826 | 15 | 72612556 | TRUE | Island | NM_052840 | TSS200 | BRUNOL6 | 1.50E-11 | 6.56E-06 | 0.55 |
| Gain | cg21883293 | 11 | 32334657 | TRUE |  |  |  |  | 1.53E-11 | 6.68E-06 | 0.55 |
| Gain | cg05247391 | 8 | 71581649 |  | Island | NM_001011720;NM_016027;NM_001011720 | 5'UTR;TSS1500;1stExon | XKR9;LACTB2;XKR9 | 1.57E-11 | 6.88E-06 | 0.56 |
| Gain | cg22851880 | 1 | 36038701 |  | N_Shore | NM_178548 | TSS1500 | TFAP2E | 1.59E-11 | 6.95E-06 | 0.56 |
| Gain | cg19891728 | 8 | 41754871 | TRUE | Island | NM_001142446 | TSS1500 | ANK1 | 1.59E-11 | 6.95E-06 | 0.56 |
| Gain | cg25316339 | 5 | 79866379 |  | Island | NM_001004441 | TSS200 | ANKRD34B | 1.65E-11 | 7.21E-06 | 0.56 |
| Gain | cg08571883 | 8 | 53854425 |  | Island |  |  |  | 1.75E-11 | 7.65E-06 | 0.56 |
| Gain | cg04169021 | 17 | 35060451 |  | Island |  |  |  | 1.76E-11 | 7.69E-06 | 0.55 |
| Gain | cg08243728 | 19 | 45905890 |  | N_Shelf | NM_001142502;NM_006663 | 5'UTR;5'UTR | PPP1R13L;PPP1R13L | 1.88E-11 | 8.21E-06 | 0.57 |
| Gain | cg12016437 | 4 | 10459163 |  | Island | NM_053042 | TSS200 | ZNF518B | 1.93E-11 | 8.44E-06 | 0.56 |
| Gain | cg05757654 | 1 | 11919554 |  | Island | NM_002521 | TSS1500 | NPPB | 1.94E-11 | 8.48E-06 | 0.56 |
| Gain | cg09278980 | 11 | 64739343 |  | Island |  |  |  | 1.96E-11 | 8.57E-06 | 0.53 |
| Gain | cg20879085 | 7 | 115850441 |  | Island | NM_015641 | TSS200 | TES | 2.01E-11 | 8.80E-06 | 0.54 |
| Gain | cg18148512 | 7 | 97557830 |  | Island |  |  |  | 2.03E-11 | 8.90E-06 | 0.56 |
| Gain | cg06231995 | 9 | 140024593 |  | Island |  |  |  | 2.07E-11 | 9.05E-06 | 0.54 |
| Gain | cg16367511 | 2 | 74425523 |  | Island | NR_027405;NM_006636 | TSS200;TSS200 | MTHFD2;MTHFD2 | 2.24E-11 | 9.81E-06 | 0.55 |
| Gain | cg15832311 | 2 | 242675047 |  | Island | NM_152783 | Body | D2HGDH | 2.27E-11 | 9.95E-06 | 0.55 |
| Gain | cg21860429 | 6 | 105389544 |  | Island |  |  |  | 2.34E-11 | 1.02E-05 | 0.56 |
| Gain | cg21422770 | 21 | 38362632 |  | Island | NM_000411 | TSS200 | HLCS | 2.35E-11 | 1.03E-05 | 0.56 |
| Gain | cg22224597 | 1 | 41444871 |  | Island | NM_001905 | TSS200 | CTPS | 2.36E-11 | 1.03E-05 | 0.56 |
| Gain | cg07502389 | 8 | 24771259 |  | Island | NM_005382;NM_001105541 | TSS200;TSS1500 | NEFM;NEFM | 2.37E-11 | 1.04E-05 | 0.56 |
| Gain | cg00032205 | 8 | 98290372 |  | Island | NM_033512 | TSS200 | TSPYL5 | 2.40E-11 | 1.05E-05 | 0.54 |
| Gain | cg01541867 | 9 | 86755499 | TRUE | Island |  |  |  | 2.60E-11 | 1.14E-05 | 0.56 |
| Gain | cg04749646 | 2 | 217236630 |  | Island | NM_020814;NM_020814 | 5'UTR;1stExon | MARCH4;MARCH4 | 2.62E-11 | 1.14E-05 | 0.55 |
| Gain | cg02847037 | 13 | 22033729 |  | Island | NM_153251 | TSS1500 | ZDHHC20 | 2.67E-11 | 1.17E-05 | 0.56 |
| Gain | cg17437852 | 20 | 2490109 |  | S_Shore | NM_024325 | TSS1500 | ZNF343 | 2.84E-11 | 1.24E-05 | 0.55 |
| Gain | cg26112929 | 7 | 5821500 |  | S_Shore | NM_207116;NM_207111 | TSS1500;TSS1500 | RNF216;RNF216 | 2.88E-11 | 1.26E-05 | 0.55 |
| Gain | cg02748419 | 3 | 141868612 |  | Island |  |  |  | 2.97E-11 | 1.30E-05 | 0.56 |
| Gain | cg11915218 | 17 | 40335849 | TRUE | N_Shore |  |  |  | 2.99E-11 | 1.31E-05 | 0.55 |
| Gain | cg06061878 | 11 | 122526279 |  | Island | NM_032873 | TSS200 | UBASH3B | 3.08E-11 | 1.35E-05 | 0.56 |
| Gain | cg26367730 | 8 | 41386454 |  | Island | NM_032336 | TSS1500 | GINS4 | 3.08E-11 | 1.35E-05 | 0.55 |
| Gain | cg15993083 | 12 | 4381788 |  | Island | NM_001759 | TSS1500 | CCND2 | 3.14E-11 | 1.38E-05 | 0.56 |
| Gain | cg24637426 | 3 | 192233231 |  | S_Shore | NM_004113 | Body | FGF12 | 3.19E-11 | 1.40E-05 | 0.55 |
| Gain | cg27509306 | 12 | 510604 |  | Island | NM_001130146;NM_001130147;NM_032358;NM_001130148 | TSS200;5'UTR;TSS200;5'UTR | CCDC77;CCDC77;CCDC77;CCDC77 | 3.20E-11 | 1.40E-05 | 0.55 |
| Gain | cg01912040 | 17 | 1106553 |  | N_Shore |  |  |  | 3.28E-11 | 1.43E-05 | 0.51 |
| Gain | cg06603828 | 5 | 149829668 |  | S_Shore | NM_001025070;NM_005617;NM_001025071 | TSS1500;TSS1500;TSS1500 | RPS14;RPS14;RPS14 | 3.31E-11 | 1.45E-05 | 0.54 |
| Gain | cg20797766 | 16 | 2770866 |  | N_Shore | NM_031948 | TSS1500 | PRSS27 | 3.72E-11 | 1.63E-05 | 0.54 |
| Gain | cg23500537 | 5 | 140419819 |  |  |  |  |  | 3.88E-11 | 1.70E-05 | 0.53 |
| Gain | cg08095852 | 4 | 176987020 |  | Island | NM_181265;NM_181265;NM_170710;NM_170710 | 1stExon;5'UTR;1stExon;5'UTR | WDR17;WDR17;WDR17;WDR17 | 3.88E-11 | 1.70E-05 | 0.55 |
| Gain | cg22395019 | 2 | 31361692 |  | Island | NM_024572 | TSS200 | GALNT14 | 3.90E-11 | 1.71E-05 | 0.55 |
| Gain | cg02734527 | 16 | 4303413 |  | N_Shore |  |  |  | 4.01E-11 | 1.75E-05 | 0.55 |
| Gain | cg05396044 | 1 | 173836727 |  | N_Shore | NR_002578;NR_003942;NR_003941;NR_003943;NM_032522;NM_001122770 | Body;TSS1500;TSS1500;TSS1500;TSS1500;TSS1500 | GAS5;SNORD76;SNORD75;SNORD77;ZBTB37;ZBTB37 | 4.06E-11 | 1.78E-05 | 0.53 |
| Gain | cg26490949 | 13 | 20532080 |  | Island | NM_003453;NM_197968 | TSS1500;TSS1500 | ZMYM2;ZMYM2 | 4.11E-11 | 1.80E-05 | 0.54 |
| Gain | cg07720856 | 2 | 232572668 |  | Island | NM_001099285;NM_002823 | TSS1500;TSS1500 | PTMA;PTMA | 4.28E-11 | 1.87E-05 | 0.55 |
| Gain | cg01471153 | 15 | 34876295 |  | Island | NR_027410 | TSS1500 | GOLGA8B | 4.38E-11 | 1.92E-05 | 0.54 |
| Gain | cg00059225 | 5 | 151304357 | TRUE | Island | NM_000171;NM_001146040;NM_000171;NM_001146040 | 1stExon;1stExon;5'UTR;5'UTR | GLRA1;GLRA1;GLRA1;GLRA1 | 4.45E-11 | 1.95E-05 | 0.54 |
| Gain | cg05308656 | 2 | 235406291 |  | Island | NM_005737 | TSS1500 | ARL4C | 4.52E-11 | 1.98E-05 | 0.54 |
| Gain | cg16541852 | 2 | 219763059 | TRUE | Island |  |  |  | 4.55E-11 | 1.99E-05 | 0.55 |
| Gain | cg06707236 | 19 | 911965 |  | Island | NM_138774 | Body | C19orf22 | 4.57E-11 | 2.00E-05 | 0.53 |
| Gain | cg09609212 | 15 | 74284801 |  | Island | NM_004809 | TSS200 | STOML1 | 4.72E-11 | 2.06E-05 | 0.54 |
| Gain | cg01352586 | 1 | 202995473 |  | Island |  |  |  | 4.87E-11 | 2.13E-05 | 0.54 |
| Gain | cg25505610 | 11 | 32605184 |  | Island | NM_006360 | TSS1500 | EIF3M | 5.06E-11 | 2.21E-05 | 0.54 |
| Gain | cg14480507 | 4 | 6021538 | TRUE |  |  |  |  | 5.09E-11 | 2.23E-05 | 0.55 |
| Gain | cg05492839 | 6 | 80657430 |  | Island | NM_022726 | TSS200 | ELOVL4 | 5.12E-11 | 2.24E-05 | 0.55 |
| Gain | cg12641275 | 8 | 11411487 |  |  | NM_001715 | Body | BLK | 5.30E-11 | 2.32E-05 | 0.55 |
| Gain | cg10825040 | 8 | 82633715 |  | S_Shore | NM_001170797;NM_001170796;NR_033194;NM_024699;NR_033195;NR_033196;NR_033193 | TSS200;TSS200;TSS200;TSS200;TSS200;TSS200;TSS200 | ZFAND1;ZFAND1;ZFAND1;ZFAND1;ZFAND1;ZFAND1;ZFAND1 | 5.34E-11 | 2.34E-05 | 0.54 |
| Gain | cg13201172 | 8 | 144891810 |  | Island | NM_015356;NM_182706 | Body;Body | SCRIB;SCRIB | 5.56E-11 | 2.43E-05 | 0.55 |
| Gain | cg12189835 | 11 | 61335071 |  | N_Shore | NM_004200 | Body | SYT7 | 5.58E-11 | 2.44E-05 | 0.55 |
| Gain | cg12451153 | 22 | 37099549 |  | Island | NM_006078 | TSS1500 | CACNG2 | 5.59E-11 | 2.45E-05 | 0.55 |
| Gain | cg22088594 | 13 | 37634133 |  | S_Shore | NM_017569;NM_001014286 | TSS1500;TSS1500 | FAM48A;FAM48A | 5.61E-11 | 2.46E-05 | 0.54 |
| Gain | cg25334393 | 8 | 145955671 |  | Island | NM_138367 | Body | ZNF251 | 5.61E-11 | 2.46E-05 | 0.53 |
| Gain | cg01693650 | 3 | 45883855 |  | Island | NM_020347 | TSS1500 | LZTFL1 | 5.64E-11 | 2.47E-05 | 0.54 |
| Gain | cg19410770 | 6 | 28806514 |  | N_Shore |  |  |  | 5.67E-11 | 2.48E-05 | 0.55 |
| Gain | cg02903907 | 1 | 232941055 | TRUE | Island | NM_019090 | 1stExon | KIAA1383 | 5.83E-11 | 2.55E-05 | 0.55 |
| Gain | cg00516222 | 17 | 27949795 | TRUE | Island | NM_032854 | TSS1500 | CORO6 | 5.84E-11 | 2.56E-05 | 0.54 |
| Gain | cg08120263 | 14 | 29236013 |  | N_Shore | NM_005249 | TSS1500 | FOXG1 | 5.85E-11 | 2.56E-05 | 0.55 |
| Gain | cg07059052 | 16 | 2828302 |  | S_Shore | NM_207013;NM_007108 | TSS1500;TSS1500 | TCEB2;TCEB2 | 5.90E-11 | 2.58E-05 | 0.55 |
| Gain | cg07589899 | 2 | 62020677 |  |  |  |  |  | 6.05E-11 | 2.65E-05 | 0.55 |
| Gain | cg19743881 | 7 | 115850438 |  | Island | NM_015641 | TSS200 | TES | 6.15E-11 | 2.69E-05 | 0.52 |
| Gain | cg04420878 | 8 | 56987290 |  | Island | NM_001146227;NR_002437;NM_001023 | TSS200;TSS1500;TSS200 | RPS20;SNORD54;RPS20 | 6.17E-11 | 2.70E-05 | 0.54 |
| Gain | cg26147554 | 18 | 712733 |  | Island | NM_017512;NM_001126123;NM_202758 | TSS200;TSS200;TSS1500 | ENOSF1;ENOSF1;ENOSF1 | 6.21E-11 | 2.72E-05 | 0.55 |
| Gain | cg23956238 | 19 | 55672036 | TRUE | Island | NM_178837 | Body | C19orf51 | 6.24E-11 | 2.73E-05 | 0.54 |
| Gain | cg07420163 | 16 | 89778301 | TRUE | Island | NM_004913 | Body | C16orf7 | 6.39E-11 | 2.80E-05 | 0.55 |
| Gain | cg02479575 | 19 | 4769653 |  |  | NR_029607;NR_027148 | TSS1500;Body | MIR7-3;C19orf30 | 6.57E-11 | 2.88E-05 | 0.54 |
| Gain | cg03015610 | 16 | 58163699 |  | S_Shore | NM_013242 | TSS1500 | C16orf80 | 6.63E-11 | 2.90E-05 | 0.53 |
| Gain | cg24422198 | 18 | 59992387 |  | Island | NM_003839 | TSS200 | TNFRSF11A | 6.85E-11 | 3.00E-05 | 0.55 |
| Gain | cg16510278 | 2 | 20063376 |  |  |  |  |  | 7.06E-11 | 3.09E-05 | 0.54 |
| Gain | cg13814485 | 10 | 8095500 | TRUE | Island | NR_024256;NM_002051;NM_001002295;NR_024255 | TSS200;TSS1500;TSS1500;TSS200 | FLJ45983;GATA3;GATA3;FLJ45983 | 7.52E-11 | 3.29E-05 | 0.55 |
| Gain | cg13347071 | 2 | 210636748 |  | Island | NM_182587;NM_182587;NM_032504;NM_032504 | 1stExon;5'UTR;1stExon;5'UTR | UNC80;UNC80;UNC80;UNC80 | 7.52E-11 | 3.29E-05 | 0.52 |
| Gain | cg07671586 | 3 | 131754122 |  | Island | NM_130808 | TSS1500 | CPNE4 | 7.62E-11 | 3.34E-05 | 0.55 |
| Gain | cg00664406 | 3 | 51740875 | TRUE | Island | NM_000839;NM_001130063 | TSS1500;TSS1500 | GRM2;GRM2 | 8.29E-11 | 3.63E-05 | 0.53 |
| Gain | cg24004532 | 6 | 53517075 | TRUE | Island | NM_001003760 | Body | KLHL31 | 8.72E-11 | 3.82E-05 | 0.55 |
| Gain | cg08160331 | 11 | 75140865 | TRUE | Island | NM_001039548 | 1stExon | KLHL35 | 8.75E-11 | 3.83E-05 | 0.53 |
| Gain | cg17988780 | 6 | 30698780 |  |  | NM_005803 | Body | FLOT1 | 9.33E-11 | 4.08E-05 | 0.54 |
| Gain | cg23652182 | 5 | 180631604 |  | Island | NM_033342;NM_203293;NM_203294;NM_203295;NM_203296 | 1stExon;1stExon;TSS1500;TSS1500;TSS1500 | TRIM7;TRIM7;TRIM7;TRIM7;TRIM7 | 9.63E-11 | 4.21E-05 | 0.52 |
| Gain | cg21683284 | 1 | 65774857 |  | N_Shore | NM_014787 | Body | DNAJC6 | 9.74E-11 | 4.26E-05 | 0.54 |
| Gain | cg14594876 | 20 | 32031951 |  | Island | NM_003098 | TSS1500 | SNTA1 | 1.02E-10 | 4.48E-05 | 0.54 |
| Gain | cg19639560 | 17 | 6946059 |  | Island | NM_153357 | Body | SLC16A11 | 1.02E-10 | 4.48E-05 | 0.55 |
| Gain | cg27559724 | 3 | 186524679 |  | Island | NM_181573;NM_002916 | TSS200;TSS1500 | RFC4;RFC4 | 1.03E-10 | 4.51E-05 | 0.54 |
| Gain | cg13564889 | 5 | 175298869 | TRUE | Island | NM_001008220;NM_006650 | 5'UTR;5'UTR | CPLX2;CPLX2 | 1.04E-10 | 4.56E-05 | 0.54 |
| Gain | cg08013270 | 2 | 73145744 |  | Island | NM_004097 | Body | EMX1 | 1.13E-10 | 4.93E-05 | 0.55 |
| Gain | cg19581424 | 2 | 42396302 |  | Island | NM_019063;NM_001145076 | TSS200;TSS200 | EML4;EML4 | 1.14E-10 | 4.99E-05 | 0.54 |
| Gain | cg07388347 | 12 | 133481466 |  | N_Shelf |  |  |  | 1.17E-10 | 5.10E-05 | 0.54 |
| Gain | cg26189067 | 2 | 32852828 |  | N_Shore | NM_017735 | TSS1500 | TTC27 | 1.26E-10 | 5.53E-05 | 0.54 |
| Gain | cg13460409 | 21 | 38379570 |  | S_Shore | NM_018962 | Body | DSCR6 | 1.29E-10 | 5.66E-05 | 0.53 |
| Gain | cg13220457 | 6 | 135504074 |  | Island | NM_005375;NM_001161657;NM_001161659;NM_001161658;NM_001130173;NM_001161660;NM_001161656 | Body;Body;Body;Body;Body;Body;Body;Body | MYB;MYB;MYB;MYB;MYB;MYB;MYB;MYB | 1.35E-10 | 5.91E-05 | 0.54 |
| Gain | cg01875838 | 19 | 10947446 |  | S_Shore | NM_006858 | TSS1500 | TMED1 | 1.36E-10 | 5.97E-05 | 0.54 |
| Gain | cg15337815 | 5 | 149935377 |  |  | NM_001543 | 3'UTR | NDST1 | 1.38E-10 | 6.02E-05 | 0.53 |
| Gain | cg02220965 | 16 | 31128310 |  | N_Shore | NM_032188;NM_182958 | TSS1500;TSS1500 | MYST1;MYST1 | 1.39E-10 | 6.07E-05 | 0.54 |
| Gain | cg05412990 | 2 | 20190169 |  | Island | NM_020779;NM_001006657 | TSS1500;TSS1500 | WDR35;WDR35 | 1.40E-10 | 6.13E-05 | 0.54 |
| Gain | cg22108374 | 15 | 74557527 |  |  | NM_025055 | Body | CCDC33 | 1.44E-10 | 6.31E-05 | 0.54 |
| Gain | cg09453076 | 2 | 235406275 |  | Island | NM_005737 | TSS1500 | ARL4C | 1.47E-10 | 6.41E-05 | 0.53 |
| Gain | cg27522573 | 16 | 87622478 |  |  |  |  |  | 1.48E-10 | 6.46E-05 | 0.52 |
| Gain | cg09298289 | 5 | 68485754 |  | S_Shore | NM_022909 | Body | CENPH | 1.53E-10 | 6.71E-05 | 0.54 |
| Gain | cg13783238 | 3 | 160121821 |  | S_Shelf | NM_005496;NR_029663;NM_001002800;NR_029525 | Body;TSS1500;Body;TSS1500 | SMC4;MIR15B;SMC4;MIR16-2 | 1.57E-10 | 6.88E-05 | 0.54 |
| Gain | cg23538901 | 15 | 46006849 | TRUE |  |  |  |  | 1.67E-10 | 7.30E-05 | 0.54 |
| Gain | cg21567504 | 15 | 28344477 |  | Island | NM_000275 | TSS200 | OCA2 | 1.68E-10 | 7.35E-05 | 0.54 |
| Gain | cg02078370 | 7 | 126988120 | TRUE | Island |  |  |  | 1.72E-10 | 7.54E-05 | 0.53 |
| Gain | cg09226692 | 6 | 43422490 |  | Island | NM_206539;NM_023932 | Body;Body | DLK2;DLK2 | 1.75E-10 | 7.65E-05 | 0.54 |
| Gain | cg04453050 | 3 | 51740896 | TRUE | Island | NM_000839;NM_001130063 | TSS200;TSS200 | GRM2;GRM2 | 1.78E-10 | 7.80E-05 | 0.51 |
| Gain | cg23704082 | 3 | 160167518 |  | Island | NM_173084;NM_173084 | 1stExon;5'UTR | TRIM59;TRIM59 | 1.80E-10 | 7.86E-05 | 0.54 |
| Gain | cg08286012 | 4 | 81257041 | TRUE | Island | NM_152770 | Body | C4orf22 | 1.85E-10 | 8.09E-05 | 0.53 |
| Gain | cg08224212 | 11 | 34196093 | TRUE | Island | NM_145804 | Body | ABTB2 | 1.97E-10 | 8.63E-05 | 0.53 |
| Gain | cg11706635 | 7 | 103087723 |  | S_Shore | NM_206883;NM_001167962;NM_206884;NM_198999;NM_206885 | TSS1500;TSS1500;TSS1500;TSS1500;TSS1500 | SLC26A5;SLC26A5;SLC26A5;SLC26A5;SLC26A5 | 2.00E-10 | 8.75E-05 | 0.54 |
| Gain | cg09105193 | 12 | 69327238 |  | Island | NM_198320;NM_001005502;NM_001874 | TSS1500;TSS1500;5'UTR | CPM;CPM;CPM | 2.02E-10 | 8.85E-05 | 0.52 |
| Gain | cg16002963 | 2 | 98703324 | TRUE | N_Shore | NM_144992 | TSS1500 | VWA3B | 2.04E-10 | 8.92E-05 | 0.54 |
| Gain | cg07806886 | 3 | 120626899 |  | Island | NM_014980 | TSS200 | STXBP5L | 2.05E-10 | 8.96E-05 | 0.54 |
| Gain | cg00221745 | 10 | 104179478 |  | Island | NM_024326;NM_002779 | TSS200;TSS1500 | FBXL15;PSD | 2.05E-10 | 8.97E-05 | 0.54 |
| Gain | cg00292135 | 7 | 156433068 |  | Island | NR_026865;NM_030936 | Body;TSS1500 | C7orf13;RNF32 | 2.10E-10 | 9.17E-05 | 0.54 |
| Gain | cg03525385 | 1 | 112438746 |  |  | NM_172198;NM_004980 | Body;Body | KCND3;KCND3 | 2.10E-10 | 9.18E-05 | 0.54 |
| Gain | cg02949991 | 22 | 29977262 |  | S_Shore | NM_003634 | TSS200 | NIPSNAP1 | 2.19E-10 | 9.59E-05 | 0.52 |
| Gain | cg13640100 | 1 | 33896928 |  | Island |  |  |  | 2.19E-10 | 9.60E-05 | 0.53 |
| Gain | cg23433607 | 20 | 5892338 | TRUE | Island | NM_001819 | Body | CHGB | 2.23E-10 | 9.75E-05 | 0.53 |
| Gain | cg15697646 | 3 | 49057813 |  | N_Shore | NM_199074;NM_018114;NR_029948;NM_199070;NM_199069;NM_199073 | TSS200;5'UTR;TSS200;TSS1500;TSS1500;TSS1500 | NDUFAF3;DALRD3;MIR425;NDUFAF3;NDUFAF3;NDUFAF3 | 2.27E-10 | 9.94E-05 | 0.53 |
| Gain | cg20426994 | 7 | 130418324 |  | Island | NM_138693 | 1stExon | KLF14 | 2.43E-10 | 1.06E-04 | 0.52 |
| Gain | cg03399905 | 15 | 79576060 |  | Island | NM_001146341 | 5'UTR | ANKRD34C | 2.53E-10 | 1.11E-04 | 0.52 |
| Gain | cg21483700 | 19 | 12943593 |  | Island | NM_031429 | Body | RTBDN | 2.57E-10 | 1.13E-04 | 0.52 |
| Gain | cg01799653 | 5 | 10563501 |  | Island | NM_001164440 | TSS1500 | ANKRD33B | 2.65E-10 | 1.16E-04 | 0.54 |
| Gain | cg10732215 | 10 | 22625465 |  | Island |  |  |  | 2.70E-10 | 1.18E-04 | 0.54 |
| Gain | cg21709871 | 8 | 144923606 |  | Island | NM_178564 | 5'UTR | NRBP2 | 2.78E-10 | 1.22E-04 | 0.51 |
| Gain | cg02788146 | 10 | 134717975 |  | N_Shore |  |  |  | 2.90E-10 | 1.27E-04 | 0.49 |
| Gain | cg00743094 | 13 | 100547968 |  | Island | NM_206808 | 3'UTR | CLYBL | 2.94E-10 | 1.29E-04 | 0.54 |
| Gain | cg15149655 | 2 | 98703698 | TRUE | Island | NM_144992;NM_144992 | 1stExon;5'UTR | VWA3B;VWA3B | 2.99E-10 | 1.31E-04 | 0.53 |
| Gain | cg19584530 | 2 | 241771687 | TRUE | Island |  |  |  | 3.09E-10 | 1.35E-04 | 0.53 |
| Gain | cg24903434 | 7 | 75831232 |  | Island | NM_001110199;NM_001110199 | 5'UTR;1stExon | SRRM3;SRRM3 | 3.14E-10 | 1.38E-04 | 0.53 |
| Gain | cg00092518 | 13 | 50070550 |  | Island | NM_001040443;NM_001040444;NM_001040444 | Body;1stExon;5'UTR | PHF11;PHF11;PHF11 | 3.17E-10 | 1.39E-04 | 0.53 |
| Gain | cg12401425 | 1 | 242011513 |  | Island | NM_006027;NM_130398;NM_130398;NM_003686 | TSS1500;5'UTR;1stExon;TSS1500 | EXO1;EXO1;EXO1;EXO1 | 3.19E-10 | 1.40E-04 | 0.53 |
| Gain | cg03251287 | 19 | 50836910 |  | Island |  |  |  | 3.22E-10 | 1.41E-04 | 0.51 |
| Gain | cg14521746 | 11 | 77185492 |  | Island | NM_001128620;NM_002576 | TSS1500;TSS1500 | PAK1;PAK1 | 3.33E-10 | 1.46E-04 | 0.52 |
| Gain | cg08431693 | 7 | 99680287 |  | S_Shore | NM_017715;NM_032924 | TSS1500;TSS1500 | ZNF3;ZNF3 | 3.34E-10 | 1.46E-04 | 0.52 |
| Gain | cg25390506 | 7 | 135347085 |  | Island | NM_001130929 | TSS200 | PL-5283 | 3.36E-10 | 1.47E-04 | 0.52 |
| Gain | cg07640648 | 19 | 39993697 |  | Island | NM_203486;NM_016941 | Body;Body | DLL3;DLL3 | 3.48E-10 | 1.52E-04 | 0.53 |
| Gain | cg03968755 | 4 | 85418816 | TRUE | Island | NM_006168 | 1stExon | NKX6-1 | 3.56E-10 | 1.56E-04 | 0.53 |
| Gain | cg22598841 | 1 | 113615267 |  | N_Shore | NM_014813 | TSS1500 | LRIG2 | 3.57E-10 | 1.56E-04 | 0.53 |
| Gain | cg23040782 | 1 | 6762215 |  | S_Shore | NM_018198 | TSS1500 | DNAJC11 | 3.60E-10 | 1.58E-04 | 0.53 |
| Gain | cg02933139 | 11 | 133817324 | TRUE |  | NM_014987 | Body | IGSF9B | 3.65E-10 | 1.60E-04 | 0.53 |
| Gain | cg02945056 | 7 | 127225891 |  | Island | NM_024523 | TSS1500 | GCC1 | 3.75E-10 | 1.64E-04 | 0.52 |
| Loss | cg05460965 | 6 | 36645100 | TRUE | N_Shore | NM_078467;NM_000389 | TSS1500;TSS1500 | CDKN1A;CDKN1A | 3.76E-10 | 1.64E-04 | -0.53 |
| Gain | cg08767938 | 7 | 25989524 |  | N_Shore |  |  |  | 3.76E-10 | 1.64E-04 | 0.52 |
| Gain | cg16986298 | 1 | 27324471 |  | S_Shelf | NM_001013642 | 3'UTR | TRNP1 | 3.85E-10 | 1.69E-04 | 0.52 |
| Gain | cg08655662 | 6 | 41438590 |  | Island |  |  |  | 3.89E-10 | 1.70E-04 | 0.52 |
| Gain | cg20345589 | 10 | 104179471 |  | Island | NM_024326;NM_002779 | TSS200;TSS1500 | FBXL15;PSD | 3.91E-10 | 1.71E-04 | 0.53 |
| Gain | cg03578662 | 1 | 40157289 | TRUE | Island | NM_016257 | TSS1500 | HPCAL4 | 4.03E-10 | 1.76E-04 | 0.51 |
| Gain | cg13894021 | 22 | 23487383 |  | Island | NM_004914 | TSS200 | RAB36 | 4.06E-10 | 1.78E-04 | 0.53 |
| Gain | cg18982073 | 17 | 42275458 |  | N_Shore | NM_001098833;NM_020218;NM_020218;NM_001098833 | 1stExon;1stExon;5'UTR;5'UTR | ATXN7L3;ATXN7L3;ATXN7L3;ATXN7L3 | 4.16E-10 | 1.82E-04 | 0.51 |
| Gain | cg25397922 | 4 | 113431869 | TRUE | Island |  |  |  | 4.18E-10 | 1.83E-04 | 0.53 |
| Gain | cg23861715 | 9 | 99382235 |  | Island | NM_003671;NM_033331 | TSS200;TSS200 | CDC14B;CDC14B | 4.19E-10 | 1.84E-04 | 0.51 |
| Gain | cg18928900 | 6 | 170605493 | TRUE | Island |  |  |  | 4.43E-10 | 1.94E-04 | 0.52 |
| Gain | cg23553442 | 9 | 74062096 |  | Island |  |  |  | 4.45E-10 | 1.95E-04 | 0.50 |
| Gain | cg24159214 | 3 | 50402751 | TRUE | Island | NM_001005505;NM_006030 | Body;Body | CACNA2D2;CACNA2D2 | 4.45E-10 | 1.95E-04 | 0.53 |
| Gain | cg04845466 | 2 | 27665079 |  | Island | NM_001168364;NM_013392;NM_173853 | TSS200;3'UTR;TSS200 | KRTCAP3;NRBP1;KRTCAP3 | 4.51E-10 | 1.97E-04 | 0.53 |
| Gain | cg16480692 | 16 | 3355295 |  | Island | NM_153028 | TSS200 | ZNF75A | 4.57E-10 | 2.00E-04 | 0.52 |
| Gain | cg20760116 | 3 | 13324924 |  | Island |  |  |  | 4.68E-10 | 2.05E-04 | 0.52 |
| Gain | cg26816688 | 7 | 156433669 |  | Island | NR_026865;NM_030936 | TSS1500;5'UTR | C7orf13;RNF32 | 4.69E-10 | 2.05E-04 | 0.53 |
| Gain | cg04986675 | 8 | 97657294 | TRUE | Island | NM_016134 | TSS1500 | PGCP | 4.72E-10 | 2.07E-04 | 0.52 |
| Gain | cg15822346 | 6 | 111408761 | TRUE | Island | NM_018593 | TSS200 | SLC16A10 | 4.80E-10 | 2.10E-04 | 0.51 |
| Gain | cg19106932 | 17 | 61926700 |  | Island |  |  |  | 4.85E-10 | 2.12E-04 | 0.52 |
| Gain | cg21072025 | 19 | 47137863 |  | Island | NM_033258 | 1stExon | GNG8 | 4.90E-10 | 2.15E-04 | 0.53 |
| Gain | cg14392031 | 9 | 107526202 |  | Island | NM_018376 | TSS1500 | NIPSNAP3B | 5.18E-10 | 2.26E-04 | 0.51 |
| Gain | cg07243161 | 6 | 136871709 |  | Island | NM_003980;NM_003980 | 5'UTR;1stExon | MAP7;MAP7 | 5.20E-10 | 2.27E-04 | 0.53 |
| Gain | cg25670583 | 4 | 715865 |  | N_Shore | NM_006315 | 5'UTR | PCGF3 | 5.22E-10 | 2.28E-04 | 0.53 |
| Gain | cg26355004 | 4 | 37455756 |  | Island | NM_001104629 | 5'UTR | C4orf19 | 5.23E-10 | 2.29E-04 | 0.53 |
| Gain | cg25590826 | 15 | 74557537 |  |  | NM_025055 | Body | CCDC33 | 5.39E-10 | 2.36E-04 | 0.52 |
| Gain | cg08541297 | 7 | 45962236 | TRUE | S_Shore | NM_000598;NM_001013398 | TSS1500;TSS1500 | IGFBP3;IGFBP3 | 5.49E-10 | 2.40E-04 | 0.53 |
| Gain | cg09547119 | 19 | 52391367 |  | Island | NR_024181;NM_032679;NM_001135590 | TSS200;TSS200;TSS200 | ZNF577;ZNF577;ZNF577 | 5.52E-10 | 2.42E-04 | 0.50 |
| Gain | cg19421368 | 20 | 45280288 |  | S_Shore | NM_022829;NM_001011554 | TSS200;5'UTR | SLC13A3;SLC13A3 | 5.57E-10 | 2.44E-04 | 0.53 |
| Gain | cg14107807 | 6 | 84221849 |  |  | NM_153362;NM_001170423 | TSS1500;TSS1500 | PRSS35;PRSS35 | 5.60E-10 | 2.45E-04 | 0.53 |
| Loss | cg24315421 | 1 | 208040253 | TRUE | N_Shore |  |  |  | 5.71E-10 | 2.50E-04 | -0.52 |
| Gain | cg01329687 | 16 | 11761803 |  | N_Shore | NM_003498 | TSS1500 | SNN | 5.77E-10 | 2.53E-04 | 0.53 |
| Gain | cg14844236 | 12 | 123753212 | TRUE | N_Shore | NM_004642 | Body | CDK2AP1 | 5.87E-10 | 2.57E-04 | 0.52 |
| Gain | cg22149516 | 5 | 101632314 |  | Island | NM_180991 | TSS200 | SLCO4C1 | 5.96E-10 | 2.61E-04 | 0.52 |
| Gain | cg15000966 | 17 | 73128123 |  | S_Shore | NM_014595 | TSS1500 | NT5C | 6.09E-10 | 2.67E-04 | 0.52 |
| Gain | cg09579953 | 2 | 101925467 |  | S_Shore | NM_173647 | TSS1500 | RNF149 | 6.12E-10 | 2.68E-04 | 0.51 |
| Gain | cg01929377 | 13 | 37634127 |  | S_Shore | NM_017569;NM_001014286 | TSS1500;TSS1500 | FAM48A;FAM48A | 6.21E-10 | 2.72E-04 | 0.50 |
| Gain | cg04239375 | 9 | 107526778 |  | Island | NM_018376 | Body | NIPSNAP3B | 6.23E-10 | 2.73E-04 | 0.51 |
| Gain | cg01627823 | 14 | 37125531 |  | Island | NM_006194 | TSS1500 | PAX9 | 6.25E-10 | 2.74E-04 | 0.53 |
| Gain | cg13442820 | 19 | 1672531 |  | Island |  |  |  | 6.61E-10 | 2.89E-04 | 0.52 |
| Gain | cg13844474 | 20 | 50159653 |  | S_Shore | NM_012340;NM_001136021;NM_173091 | TSS1500;Body;TSS1500 | NFATC2;NFATC2;NFATC2 | 6.62E-10 | 2.90E-04 | 0.53 |
| Gain | cg05936895 | 1 | 32816928 |  | N_Shore |  |  |  | 6.73E-10 | 2.95E-04 | 0.51 |
| Gain | cg08694014 | 16 | 68482591 |  | Island | NM_018667 | TSS200 | SMPD3 | 7.11E-10 | 3.11E-04 | 0.52 |
| Gain | cg16792632 | 14 | 64761208 | TRUE | Island | NM_001040275;NM_001437;NM_001040276 | TSS200;TSS200;5'UTR | ESR2;ESR2;ESR2 | 7.19E-10 | 3.15E-04 | 0.52 |
| Gain | cg02560808 | 16 | 70835289 |  | Island | NM_018052 | TSS1500 | VAC14 | 7.22E-10 | 3.16E-04 | 0.52 |
| Gain | cg02805871 | 17 | 6939695 |  | Island | NM_201566;NM_201566 | 5'UTR;1stExon | SLC16A13;SLC16A13 | 7.26E-10 | 3.18E-04 | 0.50 |
| Gain | cg14424579 | 2 | 27274309 |  | Island | NM_021831;NM_001035507 | TSS200;TSS200 | AGBL5;AGBL5 | 7.32E-10 | 3.20E-04 | 0.52 |
| Gain | cg09871669 | 10 | 7860012 |  | N_Shore | NM_031923 | TSS1500 | TAF3 | 7.36E-10 | 3.22E-04 | 0.53 |
| Gain | cg06475223 | 18 | 21498693 |  |  | NM_001127718;NM_000227;NM_198129;NM_001127717 | Body;Body;Body;Body | LAMA3;LAMA3;LAMA3;LAMA3 | 7.47E-10 | 3.27E-04 | 0.49 |
| Gain | cg20382695 | 10 | 116853814 |  | Island | NM_207303 | Body | ATRNL1 | 7.56E-10 | 3.31E-04 | 0.52 |
| Gain | cg16032102 | 1 | 206680459 |  | Island | NM_182664;NM_182663 | TSS1500;TSS1500 | RASSF5;RASSF5 | 7.68E-10 | 3.36E-04 | 0.52 |
| Gain | cg12946225 | 19 | 3573751 |  | Island | NM_006339 | Body | HMG20B | 8.10E-10 | 3.54E-04 | 0.50 |
| Gain | cg22367678 | 1 | 33077705 | TRUE |  |  |  |  | 8.16E-10 | 3.57E-04 | 0.52 |
| Gain | cg18557556 | 12 | 75905681 |  | S_Shore | NM_007043 | TSS1500 | KRR1 | 8.20E-10 | 3.59E-04 | 0.52 |
| Gain | cg14023774 | 6 | 33246157 |  | S_Shore | NM_003782 | 1stExon | B3GALT4 | 8.26E-10 | 3.62E-04 | 0.53 |
| Gain | cg09409930 | 1 | 16268878 |  | N_Shore | NM_003443 | Body | ZBTB17 | 8.36E-10 | 3.66E-04 | 0.50 |
| Gain | cg03710354 | 17 | 72919704 | TRUE | Island | NM_178160;NM_173477 | TSS1500;TSS1500 | OTOP2;USH1G | 8.49E-10 | 3.72E-04 | 0.52 |
| Gain | cg18267374 | 8 | 24771273 |  | Island | NM_001105541;NM_005382;NM_005382 | TSS1500;5'UTR;1stExon | NEFM;NEFM;NEFM | 8.51E-10 | 3.72E-04 | 0.52 |
| Gain | cg03110382 | 10 | 98956268 | TRUE | Island |  |  |  | 8.54E-10 | 3.74E-04 | 0.52 |
| Gain | cg07520506 | 12 | 7593007 |  | Island | NM_174941 | Body | CD163L1 | 8.82E-10 | 3.86E-04 | 0.52 |
| Gain | cg06858294 | 17 | 7983203 | TRUE | Island | NM_001139 | Body | ALOX12B | 8.84E-10 | 3.87E-04 | 0.51 |
| Gain | cg18145080 | 8 | 144640397 |  | Island | NM_024736;NM_001166237 | TSS200;5'UTR | GSDMD;GSDMD | 8.89E-10 | 3.89E-04 | 0.50 |
| Gain | cg01717446 | 2 | 71503566 |  | Island |  |  |  | 9.00E-10 | 3.94E-04 | 0.51 |
| Gain | cg16379337 | 7 | 115850450 |  | Island | NM_015641 | TSS200 | TES | 9.02E-10 | 3.95E-04 | 0.51 |
| Gain | cg22168941 | 6 | 126071319 |  | Island | NM_012259 | Body | HEY2 | 9.05E-10 | 3.96E-04 | 0.51 |
| Gain | cg16386080 | 9 | 90589146 | TRUE | N_Shore | NM_012119;NM_001170639;NM_178432;NM_001039803;NM_001170640 | Body;Body;Body;Body;Body | CDK20;CDK20;CDK20;CDK20;CDK20 | 9.22E-10 | 4.04E-04 | 0.51 |
| Gain | cg22016859 | 22 | 50750549 |  |  | NM_001001794 | 3'UTR | FAM116B | 9.34E-10 | 4.09E-04 | 0.49 |
| Gain | cg08378505 | 17 | 73127297 |  | Island | NM_014595 | Body | NT5C | 9.39E-10 | 4.11E-04 | 0.52 |
| Gain | cg08541518 | 6 | 69942892 | TRUE |  | NM_001704 | Body | BAI3 | 9.96E-10 | 4.36E-04 | 0.51 |
| Gain | cg02232208 | 3 | 147137147 |  | Island |  |  |  | 1.00E-09 | 4.39E-04 | 0.51 |
| Gain | cg07169873 | 17 | 2652559 |  | Island | NR_031654 | TSS1500 | MIR1253 | 1.01E-09 | 4.41E-04 | 0.51 |
| Gain | cg11359984 | 3 | 123603539 |  | Island | NM_053027;NM_053028;NM_053026;NM_053025 | TSS1500;TSS1500;TSS1500;TSS1500 | MYLK;MYLK;MYLK;MYLK | 1.02E-09 | 4.44E-04 | 0.51 |
| Gain | cg19768599 | 3 | 124931727 |  | Island | NM_024628 | TSS200 | SLC12A8 | 1.05E-09 | 4.61E-04 | 0.52 |
| Gain | cg06704773 | 1 | 173836824 |  | N_Shore | NR_002578;NR_003942;NR_003941;NR_003943;NM_032522;NM_001122770;NR_002579 | Body;TSS1500;TSS1500;TSS1500;TSS1500;TSS1500;Body | GAS5;SNORD76;SNORD75;SNORD77;ZBTB37;ZBTB37;SNORD74 | 1.06E-09 | 4.64E-04 | 0.51 |
| Gain | cg12685560 | 17 | 2302144 |  | N_Shore | NM_020310 | Body | MNT | 1.06E-09 | 4.65E-04 | 0.51 |
| Gain | cg02774015 | 1 | 15735880 |  | N_Shore | NM_024329 | TSS1500 | EFHD2 | 1.07E-09 | 4.67E-04 | 0.51 |
| Gain | cg03041738 | 1 | 228652338 | TRUE | Island |  |  |  | 1.07E-09 | 4.69E-04 | 0.52 |
| Gain | cg14415616 | 3 | 18486958 |  | Island |  |  |  | 1.10E-09 | 4.80E-04 | 0.51 |
| Gain | cg26758551 | 1 | 209921096 | TRUE | Island |  |  |  | 1.11E-09 | 4.86E-04 | 0.52 |
| Gain | cg21475150 | 2 | 101618248 |  | Island | NM_001099693;NM_000993;NM_001098577 | TSS1500;TSS1500;TSS1500 | RPL31;RPL31;RPL31 | 1.12E-09 | 4.90E-04 | 0.50 |
| Gain | cg02455820 | 16 | 51147769 |  | Island |  |  |  | 1.13E-09 | 4.96E-04 | 0.51 |
| Gain | cg22775000 | 9 | 103235181 |  | Island | NM_003692 | TSS1500 | TMEFF1 | 1.16E-09 | 5.07E-04 | 0.51 |
| Gain | cg23490822 | 19 | 10434247 |  | Island | NM_133452 | Body | RAVER1 | 1.17E-09 | 5.12E-04 | 0.51 |
| Gain | cg06385324 | 16 | 2014621 |  | Island | NR_003142;NR_003020;NM_002952 | TSS1500;TSS1500;Body | SNHG9;SNORA78;RPS2 | 1.21E-09 | 5.28E-04 | 0.50 |
| Gain | cg26060489 | 6 | 1604990 |  | Island |  |  |  | 1.22E-09 | 5.36E-04 | 0.51 |
| Gain | cg02759846 | 19 | 51227943 |  | Island | NM_002975 | Body | CLEC11A | 1.24E-09 | 5.44E-04 | 0.51 |
| Gain | cg26518431 | 1 | 47901555 |  | N_Shore | NR_026878;NM_004474 | TSS1500;TSS200 | MGC12982;FOXD2 | 1.25E-09 | 5.45E-04 | 0.51 |
| Gain | cg25942450 | 5 | 170736251 |  | Island | NM_021025 | TSS200 | TLX3 | 1.25E-09 | 5.47E-04 | 0.52 |
| Gain | cg16370875 | 6 | 80657542 |  | Island | NM_022726 | TSS1500 | ELOVL4 | 1.35E-09 | 5.90E-04 | 0.52 |
| Gain | cg07908508 | 19 | 35417702 |  | Island | NM_194325;NM_001099437;NM_001099438;NR_024018 | TSS200;TSS200;TSS200;TSS200 | ZNF30;ZNF30;ZNF30;ZNF30 | 1.36E-09 | 5.95E-04 | 0.51 |
| Gain | cg00160619 | 3 | 160823318 |  | S_Shore | NM_033167;NM_001038628;NM_003781;NM_033168;NM_033169 | TSS200;TSS200;TSS200;TSS200;TSS1500 | B3GALNT1;B3GALNT1;B3GALNT1;B3GALNT1;B3GALNT1 | 1.36E-09 | 5.95E-04 | 0.51 |
| Gain | cg08888178 | 19 | 344981 |  | N_Shore | NM_017550 | TSS200 | MIER2 | 1.37E-09 | 5.99E-04 | 0.52 |
| Gain | cg02189888 | 19 | 46582065 |  | Island |  |  |  | 1.40E-09 | 6.12E-04 | 0.50 |
| Gain | cg24074033 | 11 | 20180437 |  | N_Shore | NM_001029865 | Body | DBX1 | 1.40E-09 | 6.13E-04 | 0.51 |
| Gain | cg04269188 | 14 | 105767882 |  | Island | NM_001519 | TSS1500 | BRF1 | 1.42E-09 | 6.19E-04 | 0.52 |
| Gain | cg07131451 | 4 | 113437276 |  | Island | NM_024019;NM_024019 | 1stExon;5'UTR | NEUROG2;NEUROG2 | 1.44E-09 | 6.31E-04 | 0.51 |
| Gain | cg10002569 | 6 | 159525348 |  |  |  |  |  | 1.46E-09 | 6.41E-04 | 0.48 |
| Gain | cg25645310 | 10 | 116581305 |  | N_Shore | NM_020940;NM_001135051 | TSS200;TSS200 | FAM160B1;FAM160B1 | 1.47E-09 | 6.45E-04 | 0.52 |
| Gain | cg00193021 | 4 | 90758120 |  | Island | NM_001146055;NM_007308;NM_000345;NM_000345;NM_001146054;NM_001146054;NM_007308 | 5'UTR;1stExon;5'UTR;1stExon;1stExon;5'UTR;5'UTR | SNCA;SNCA;SNCA;SNCA;SNCA;SNCA;SNCA | 1.51E-09 | 6.59E-04 | 0.52 |
| Gain | cg06544316 | 11 | 93394605 |  | N_Shore | NM_033395 | TSS1500 | KIAA1731 | 1.51E-09 | 6.62E-04 | 0.52 |
| Gain | cg18417423 | 6 | 80657436 |  | Island | NM_022726 | TSS200 | ELOVL4 | 1.51E-09 | 6.63E-04 | 0.51 |
| Gain | cg22866825 | 5 | 125936419 |  | N_Shore | NM_032177 | TSS1500 | PHAX | 1.54E-09 | 6.73E-04 | 0.50 |
| Gain | cg03678609 | 3 | 148804556 |  | Island | NM_139048;NM_003071 | TSS1500;TSS1500 | HLTF;HLTF | 1.54E-09 | 6.73E-04 | 0.50 |
| Gain | cg12663656 | 12 | 120427667 |  | Island | NM_207311;NM_207311 | 1stExon;5'UTR | CCDC64;CCDC64 | 1.56E-09 | 6.82E-04 | 0.51 |
| Gain | cg02048412 | 16 | 4421654 |  | Island | NM_138440;NM_024535 | TSS200;Body | VASN;CORO7 | 1.57E-09 | 6.87E-04 | 0.49 |
| Gain | cg12938003 | 4 | 109092724 |  | Island | NR_029373;NR_029374 | TSS1500;Body | LOC641518;LOC641518 | 1.61E-09 | 7.05E-04 | 0.51 |
| Gain | cg22719623 | 6 | 154360732 |  | Island | NM_001145286;NM_001145282;NM_001145281;NM_001145283;NM_001008505;NM_001145285;NM_001008 | 1stExon;1stExon;Body;1stExon;1stExon;1stExon;1stExon;1stExon;5'UTR;1stExon;Body;1stExon | OPRM1;OPRM1;OPRM1;OPRM1;OPRM1;OPRM1;OPRM1;OPRM1;OPRM1;OPRM1;OPRM1;OPRM1 | 1.62E-09 | 7.07E-04 | 0.49 |
| Gain | cg01585703 | 15 | 40074646 |  | Island | NM_152597 | 5'UTR | FSIP1 | 1.62E-09 | 7.10E-04 | 0.50 |
| Gain | cg11051055 | 8 | 11058145 |  | Island | NM_173683 | 1stExon | XKR6 | 1.64E-09 | 7.20E-04 | 0.50 |
| Gain | cg25655593 | 20 | 48599521 |  | Island | NM_005985 | TSS200 | SNAI1 | 1.65E-09 | 7.21E-04 | 0.49 |
| Gain | cg19031565 | 17 | 72978794 |  | S_Shore |  |  |  | 1.66E-09 | 7.28E-04 | 0.51 |
| Gain | cg27024922 | 9 | 134378058 |  | N_Shore | NM_001077365;NM_001136114;NM_001077366;NM_001136113;NM_007171 | TSS1500;TSS1500;TSS1500;TSS1500;TSS1500 | POMT1;POMT1;POMT1;POMT1;POMT1 | 1.67E-09 | 7.30E-04 | 0.51 |
| Gain | cg19139210 | 20 | 13619699 |  | Island | NM_017714 | TSS200 | TASP1 | 1.67E-09 | 7.30E-04 | 0.50 |
| Gain | cg19586576 | 17 | 42906845 |  | Island | NM_001080383;NM_005497 | 5'UTR;5'UTR | GJC1;GJC1 | 1.68E-09 | 7.35E-04 | 0.51 |
| Loss | cg04256466 | 4 | 169752398 |  | N_Shore | NM_016081;NM_001166109;NM_001166108;NM_001166110 | Body;Body;Body;TSS1500 | PALLD;PALLD;PALLD;PALLD | 1.70E-09 | 7.45E-04 | -0.51 |
| Gain | cg09630437 | 19 | 2740301 |  | Island | NM_213568;NM_144564 | TSS1500;TSS1500 | SLC39A3;SLC39A3 | 1.82E-09 | 7.94E-04 | 0.50 |
| Gain | cg01568784 | 11 | 87908783 |  | S_Shore | NM_022337 | TSS200 | RAB38 | 1.84E-09 | 8.05E-04 | 0.51 |
| Gain | cg07184013 | 7 | 25989618 |  | N_Shore | NR_029597 | TSS200 | MIR148A | 1.89E-09 | 8.26E-04 | 0.50 |
| Gain | cg26804595 | 16 | 749581 |  | N_Shore | NM_153350 | 5'UTR | FBXL16 | 1.91E-09 | 8.35E-04 | 0.51 |
| Gain | cg13156863 | 5 | 140700516 |  | S_Shore | NM_005642 | TSS200 | TAF7 | 1.93E-09 | 8.44E-04 | 0.51 |
| Gain | cg23239612 | 5 | 137549391 |  |  | NM_004661 | TSS1500 | CDC23 | 1.96E-09 | 8.56E-04 | 0.50 |
| Gain | cg09644065 | 6 | 127835750 |  | N_Shore | NM_001012279 | Body | C6orf174 | 2.00E-09 | 8.76E-04 | 0.50 |
| Gain | cg17462962 | 5 | 154347281 |  |  | NM_001014990;NM_014180 | 3'UTR;3'UTR | MRPL22;MRPL22 | 2.02E-09 | 8.84E-04 | 0.51 |
| Gain | cg14319409 | 5 | 151304409 | TRUE | Island | NM_000171;NM_001146040 | TSS200;TSS200 | GLRA1;GLRA1 | 2.04E-09 | 8.94E-04 | 0.51 |
| Gain | cg14068796 | 6 | 28832184 |  | S_Shore |  |  |  | 2.07E-09 | 9.04E-04 | 0.51 |
| Gain | cg09849846 | 10 | 124913566 |  | N_Shore | NM_004725;NM_001007793 | TSS200;TSS200 | BUB3;BUB3 | 2.07E-09 | 9.05E-04 | 0.50 |
| Gain | cg27518993 | 3 | 52088253 | TRUE | N_Shore | NM_001947 | Body | DUSP7 | 2.10E-09 | 9.18E-04 | 0.50 |
| Gain | cg24853724 | 7 | 28997403 |  | Island | NM_014817 | 1stExon | TRIL | 2.11E-09 | 9.23E-04 | 0.50 |
| Gain | cg07687610 | 12 | 32832045 |  | N_Shore | NM_005690;NM_012063;NM_012062 | TSS200;TSS200;TSS200 | DNM1L;DNM1L;DNM1L | 2.16E-09 | 9.44E-04 | 0.50 |
| Gain | cg06074958 | 12 | 57118908 |  | N_Shore | NM_005594;NM_001113203;NM_001113202;NM_001113201;NM_005594;NM_001113201 | 1stExon;5'UTR;5'UTR;1stExon;5'UTR;5'UTR | NACA;NACA;NACA;NACA;NACA;NACA | 2.18E-09 | 9.53E-04 | 0.50 |
| Gain | cg15158376 | 18 | 712731 |  | Island | NM_017512;NM_001126123;NM_202758 | TSS200;TSS200;TSS1500 | ENOSF1;ENOSF1;ENOSF1 | 2.18E-09 | 9.54E-04 | 0.51 |
| Gain | cg25585523 | 3 | 186524693 |  | Island | NM_181573;NM_002916 | TSS1500;TSS1500 | RFC4;RFC4 | 2.19E-09 | 9.59E-04 | 0.50 |
| Gain | cg16019898 | 5 | 140700602 |  | S_Shore | NM_005642 | TSS1500 | TAF7 | 2.25E-09 | 9.87E-04 | 0.49 |
| Gain | cg13029847 | 17 | 27333273 |  | S_Shore | NM_178860;NM_001098635 | TSS200;TSS200 | SEZ6;SEZ6 | 2.28E-09 | 9.99E-04 | 0.50 |
| Gain | cg05674150 | 9 | 16872192 |  | S_Shore | NM_017637 | TSS1500 | BNC2 | 2.29E-09 | 1.00E-03 | 0.51 |
| Gain | cg02381192 | 7 | 1452425 |  | Island |  |  |  | 2.37E-09 | 1.04E-03 | 0.50 |
| Gain | cg25100532 | 11 | 10562910 |  | Island | NM_016422 | TSS200 | RNF141 | 2.41E-09 | 1.05E-03 | 0.50 |
| Gain | cg00830285 | 7 | 149119905 | TRUE | Island |  |  |  | 2.41E-09 | 1.05E-03 | 0.51 |
| Gain | cg15123562 | 6 | 44044507 | TRUE | S_Shelf |  |  |  | 2.43E-09 | 1.06E-03 | 0.50 |
| Gain | cg01019875 | 17 | 6945620 |  | Island | NM_153357 | Body | SLC16A11 | 2.46E-09 | 1.08E-03 | 0.51 |
| Gain | cg07751287 | 1 | 167599830 | TRUE | Island | NM_052862 | Body | RCSD1 | 2.47E-09 | 1.08E-03 | 0.50 |
| Gain | cg25712567 | 4 | 715872 |  | N_Shore | NM_006315 | 5'UTR | PCGF3 | 2.49E-09 | 1.09E-03 | 0.51 |
| Gain | cg09369954 | 1 | 32663763 | TRUE | N_Shelf | NM_175852 | 3'UTR | TXLNA | 2.49E-09 | 1.09E-03 | 0.49 |
| Gain | cg05218976 | 19 | 37958522 |  | N_Shore | NM_144694;NM_152484 | TSS1500;TSS200 | ZNF570;ZNF569 | 2.57E-09 | 1.12E-03 | 0.50 |
| Gain | cg09513380 | 13 | 100622253 |  | Island | NM_033132 | Body | ZIC5 | 2.57E-09 | 1.13E-03 | 0.51 |
| Gain | cg01356044 | 16 | 70720817 |  | S_Shore | NM_138383 | TSS1500 | MTSS1L | 2.58E-09 | 1.13E-03 | 0.51 |
| Gain | cg04293888 | 17 | 7343790 |  | Island | NM_004112 | Body | FGF11 | 2.59E-09 | 1.13E-03 | 0.51 |
| Gain | cg20375220 | 12 | 79257667 |  |  | NM_001135805;NM_005639 | TSS200;TSS1500 | SYT1;SYT1 | 2.59E-09 | 1.13E-03 | 0.48 |
| Gain | cg13546858 | 1 | 869346 |  | Island | NM_152486 | Body | SAMD11 | 2.66E-09 | 1.16E-03 | 0.51 |
| Gain | cg08526825 | 16 | 2802229 |  | Island | NM_016333;NR_027275 | TSS200;Body | SRRM2;LOC100128788 | 2.72E-09 | 1.19E-03 | 0.50 |
| Gain | cg06889481 | 2 | 219827660 | TRUE | Island |  |  |  | 2.74E-09 | 1.20E-03 | 0.50 |
| Gain | cg26883434 | 5 | 111091560 |  | N_Shore | NM_001142478;NM_001142481;NM_001142482;NM_004772;NM_001142474;NM_001142483;NM_001142475 | 5'UTR;5'UTR;5'UTR;5'UTR;Body;1stExon;Body;5'UTR;5'UTR;5'UTR;5'UTR;5'UTR | C5orf13;C5orf13;C5orf13;C5orf13;C5orf13;C5orf13;C5orf13;C5orf13;C5orf13;C5orf13;C5orf13 | 2.83E-09 | 1.24E-03 | 0.51 |
| Gain | cg15571730 | 7 | 99680278 |  | S_Shore | NM_017715;NM_032924 | TSS1500;TSS1500 | ZNF3;ZNF3 | 2.87E-09 | 1.26E-03 | 0.50 |
| Gain | cg11018337 | 10 | 8095495 | TRUE | Island | NR_024256;NM_002051;NM_001002295;NR_024255 | TSS200;TSS1500;TSS1500;TSS200 | FLJ45983;GATA3;GATA3;FLJ45983 | 2.87E-09 | 1.26E-03 | 0.51 |
| Gain | cg18546419 | 5 | 176433891 |  | Island | NM_016290 | TSS1500 | UIMC1 | 2.88E-09 | 1.26E-03 | 0.49 |
| Gain | cg06022942 | 10 | 8095484 | TRUE | Island | NR_024256;NM_002051;NM_001002295;NR_024255 | TSS200;TSS1500;TSS1500;TSS200 | FLJ45983;GATA3;GATA3;FLJ45983 | 2.88E-09 | 1.26E-03 | 0.51 |
| Gain | cg25015277 | 16 | 2918180 | TRUE | Island |  |  |  | 2.93E-09 | 1.28E-03 | 0.50 |
| Gain | cg27106513 | 8 | 54790372 |  | Island | NM_170587 | Body | RGS20 | 2.95E-09 | 1.29E-03 | 0.50 |
| Gain | cg00312553 | 5 | 157098553 |  | Island | NM_001145132 | TSS200 | C5orf52 | 2.95E-09 | 1.29E-03 | 0.49 |
| Gain | cg15061025 | 3 | 45883859 |  | Island | NM_020347 | TSS1500 | LZTFL1 | 2.96E-09 | 1.30E-03 | 0.50 |
| Gain | cg14901671 | 17 | 70589079 |  | Island |  |  |  | 2.98E-09 | 1.30E-03 | 0.51 |
| Gain | cg13016408 | 2 | 133426298 |  | N_Shore | NM_001077427;NM_144586 | 5'UTR;Body | LYPD1;LYPD1 | 2.98E-09 | 1.30E-03 | 0.50 |
| Gain | cg18337963 | 11 | 46383209 |  |  | NM_001105540;NM_201533;NM_001105540;NM_003646;NM_201532 | 1stExon;Body;5'UTR;Body;Body | DGKZ;DGKZ;DGKZ;DGKZ;DGKZ | 2.99E-09 | 1.31E-03 | 0.50 |
| Gain | cg08734918 | 6 | 94129481 |  | Island | NM_004440 | TSS200 | EPHA7 | 3.07E-09 | 1.34E-03 | 0.51 |
| Gain | cg14279035 | 9 | 139617442 |  | N_Shore | NM_152421 | Body | FAM69B | 3.09E-09 | 1.35E-03 | 0.50 |
| Gain | cg06365567 | 3 | 49057806 |  | N_Shore | NM_199074;NM_018114;NR_029948;NM_199070;NM_199069;NM_199073 | TSS200;5'UTR;TSS200;TSS1500;TSS1500;TSS1500 | NDUFAF3;DALRD3;MIR425;NDUFAF3;NDUFAF3;NDUFAF3 | 3.16E-09 | 1.38E-03 | 0.50 |
| Gain | cg09177567 | 8 | 1765312 | TRUE | S_Shore | NR_030326 | TSS200 | MIR596 | 3.16E-09 | 1.38E-03 | 0.50 |
| Gain | cg19677989 | 15 | 74284791 |  | Island | NM_004809 | TSS200 | STOML1 | 3.16E-09 | 1.38E-03 | 0.49 |
| Gain | cg19841423 | 20 | 62366755 |  | S_Shore | NM_181485;NM_032527;NM_001083113;NM_017806 | Body;Body;Body;TSS1500 | ZGPAT;ZGPAT;ZGPAT;LIME1 | 3.24E-09 | 1.42E-03 | 0.51 |
| Gain | cg17303833 | 10 | 97889448 |  | N_Shore | NM_014803 | TSS200 | ZNF518A | 3.25E-09 | 1.42E-03 | 0.50 |
| Gain | cg05590196 | 1 | 33220274 | TRUE | S_Shore | NM_020888 | Body | KIAA1522 | 3.28E-09 | 1.44E-03 | 0.50 |
| Gain | cg15091323 | 2 | 60780474 |  | N_Shore | NM_138559;NM_022893;NM_018014;NM_138559;NM_022893;NM_018014 | 1stExon;1stExon;1stExon;5'UTR;5'UTR;5'UTR | BCL11A;BCL11A;BCL11A;BCL11A;BCL11A;BCL11A | 3.34E-09 | 1.46E-03 | 0.51 |
| Gain | cg00601711 | 5 | 1089507 |  | S_Shore | NM_006598 | Body | SLC12A7 | 3.34E-09 | 1.46E-03 | 0.50 |
| Gain | cg12563644 | 5 | 87956996 | TRUE | Island | NR_015436 | Body | LOC645323 | 3.36E-09 | 1.47E-03 | 0.51 |
| Gain | cg22309696 | 2 | 172960993 | TRUE | Island |  |  |  | 3.37E-09 | 1.47E-03 | 0.51 |
| Gain | cg25826226 | 15 | 41953061 |  | Island | NM_001080541;NM_001164273 | 5'UTR;5'UTR | MGA;MGA | 3.39E-09 | 1.48E-03 | 0.51 |
| Gain | cg12559031 | 17 | 48046365 |  | Island | NM_138281 | TSS200 | DLX4 | 3.45E-09 | 1.51E-03 | 0.50 |
| Gain | cg03719693 | 4 | 109093430 | TRUE | Island | NR_029373;NR_029374 | Body;Body | LOC641518;LOC641518 | 3.50E-09 | 1.53E-03 | 0.51 |
| Gain | cg17652435 | 17 | 36103066 |  | Island | NM_000458;NM_001165923 | Body;Body | HNF1B;HNF1B | 3.57E-09 | 1.56E-03 | 0.51 |
| Gain | cg24150153 | 1 | 94703463 |  | S_Shore | NM_004815 | TSS200 | ARHGAP29 | 3.60E-09 | 1.57E-03 | 0.50 |
| Gain | cg16549027 | 6 | 43149629 |  | N_Shore | NM_015089 | TSS1500 | CUL9 | 3.72E-09 | 1.63E-03 | 0.49 |
| Gain | cg05176991 | 18 | 24128116 |  | Island | NM_001136205;NM_198991;NM_001142730 | 5'UTR;5'UTR;1stExon | KCTD1;KCTD1;KCTD1 | 3.75E-09 | 1.64E-03 | 0.50 |
| Gain | cg13805608 | 18 | 12948653 |  | Island | NM_031216;NM_001013437 | Body;Body | SEH1L;SEH1L | 3.75E-09 | 1.64E-03 | 0.49 |
| Gain | cg09499629 | 7 | 130419136 |  | Island | NM_138693 | TSS1500 | KLF14 | 3.75E-09 | 1.64E-03 | 0.48 |
| Gain | cg10795738 | 5 | 33936171 | TRUE | Island | NM_016568 | TSS1500 | RXFP3 | 3.76E-09 | 1.65E-03 | 0.50 |
| Gain | cg13503413 | 10 | 60937257 |  | S_Shore | NM_001143774;NM_032439;NM_001143774 | 5'UTR;Body;1stExon | PHYHIPL;PHYHIPL;PHYHIPL | 3.77E-09 | 1.65E-03 | 0.49 |
| Gain | cg10432620 | 16 | 74808963 | TRUE | Island | NM_024306 | TSS1500 | FA2H | 3.78E-09 | 1.65E-03 | 0.50 |
| Gain | cg26272477 | 14 | 100437554 |  | Island |  |  |  | 3.86E-09 | 1.69E-03 | 0.50 |
| Loss | cg07122805 | 17 | 67754138 | TRUE |  |  |  |  | 4.06E-09 | 1.78E-03 | -0.50 |
| Gain | cg04079139 | 1 | 26735245 |  | N_Shelf |  |  |  | 4.11E-09 | 1.80E-03 | 0.50 |
| Gain | cg10102418 | 12 | 510594 |  | Island | NM_001130146;NM_001130147;NM_032358;NM_001130148 | TSS200;5'UTR;TSS200;5'UTR | CCDC77;CCDC77;CCDC77;CCDC77 | 4.13E-09 | 1.81E-03 | 0.50 |
| Gain | cg07121644 | 11 | 46383031 |  |  | NM_201533;NM_001105540;NM_003646;NM_201532 | Body;TSS200;Body;Body | DGKZ;DGKZ;DGKZ;DGKZ | 4.16E-09 | 1.82E-03 | 0.49 |
| Gain | cg21453443 | 1 | 228645627 |  | Island | NM_033445;NM_175055 | TSS200;TSS200 | HIST3H2A;HIST3H2BB | 4.18E-09 | 1.83E-03 | 0.50 |
| Gain | cg20977794 | 14 | 51560748 |  | Island | NM_015163;NM_052978 | Body;Body | TRIM9;TRIM9 | 4.18E-09 | 1.83E-03 | 0.49 |
| Gain | cg00991848 | 16 | 2014270 |  | Island | NR_003142;NR_002326;NR_003020;NM_002952 | TSS1500;TSS1500;TSS1500;Body | SNHG9;SNORA64;SNORA78;RPS2 | 4.18E-09 | 1.83E-03 | 0.49 |
| Gain | cg16181396 | 3 | 147126206 |  | N_Shore | NM_003412 | TSS1500 | ZIC1 | 4.20E-09 | 1.84E-03 | 0.51 |
| Gain | cg08592707 | 17 | 56833076 |  | Island | NM_014906 | TSS200 | PPM1E | 4.21E-09 | 1.84E-03 | 0.50 |
| Gain | cg10894072 | 1 | 51985215 |  | Island | NM_001981 | TSS1500 | EPS15 | 4.21E-09 | 1.84E-03 | 0.48 |
| Gain | cg07848706 | 17 | 8906601 |  | Island |  |  |  | 4.23E-09 | 1.85E-03 | 0.50 |
| Gain | cg16896647 | 9 | 93563776 |  | Island | NM_001135052;NR_024156;NM_003177 | TSS1500;TSS1500;TSS1500 | SYK;SYK;SYK | 4.30E-09 | 1.88E-03 | 0.49 |
| Gain | cg12899421 | 1 | 151512592 |  | N_Shore | NM_020127;NM_001126337 | TSS200;TSS200 | TUFT1;TUFT1 | 4.30E-09 | 1.88E-03 | 0.50 |
| Gain | cg14305262 | 11 | 598487 |  | S_Shore | NM_020901 | Body | PHRF1 | 4.34E-09 | 1.90E-03 | 0.50 |
| Gain | cg10665379 | 5 | 65891878 |  | Island | NM_198828;NM_001164664 | TSS1500;TSS1500 | MAST4;MAST4 | 4.35E-09 | 1.90E-03 | 0.49 |
| Gain | cg12603531 | 9 | 139258402 |  | Island | NM_001080849 | TSS200 | DNLZ | 4.42E-09 | 1.93E-03 | 0.50 |
| Gain | cg16219246 | 4 | 57396890 | TRUE | Island |  |  |  | 4.42E-09 | 1.93E-03 | 0.50 |
| Gain | cg25162948 | 13 | 50070718 |  | Island | NM_001040443;NM_001040444 | Body;5'UTR | PHF11;PHF11 | 4.44E-09 | 1.94E-03 | 0.50 |
| Gain | cg14371731 | 10 | 81003175 |  | Island | NM_020338 | Body | ZMIZ1 | 4.54E-09 | 1.99E-03 | 0.50 |
| Gain | cg00180930 | 6 | 31746832 |  |  | NM_006295 | Body | VARS | 4.56E-09 | 2.00E-03 | 0.49 |
| Gain | cg03602730 | 3 | 120626911 |  | Island | NM_014980 | TSS200 | STXBP5L | 4.59E-09 | 2.01E-03 | 0.50 |
| Gain | cg20777920 | 6 | 10404848 |  | Island | NM_001042425;NM_001032280;NM_003220 | Body;Body;Body | TFAP2A;TFAP2A;TFAP2A | 4.62E-09 | 2.02E-03 | 0.50 |
| Gain | cg21899500 | 3 | 51740850 | TRUE | Island | NM_000839;NM_001130063 | TSS1500;TSS1500 | GRM2;GRM2 | 4.64E-09 | 2.03E-03 | 0.47 |
| Gain | cg09379755 | 3 | 62861925 |  | S_Shore | NM_183393;NM_003716;NM_183394 | TSS1500;TSS1500;TSS1500 | CADPS;CADPS;CADPS | 4.66E-09 | 2.04E-03 | 0.50 |
| Gain | cg22579950 | 13 | 50654649 |  | N_Shore | NR_002612 | Body | DLEU2 | 4.69E-09 | 2.05E-03 | 0.50 |
| Gain | cg01837410 | 5 | 101631990 |  | N_Shore | NM_180991;NM_180991 | 1stExon;5'UTR | SLCO4C1;SLCO4C1 | 4.82E-09 | 2.11E-03 | 0.50 |
| Gain | cg19921279 | 1 | 32827389 | TRUE | Island | NM_001167676;NM_052841 | Body;TSS1500 | LOC100128071;TSSK3 | 4.83E-09 | 2.11E-03 | 0.49 |
| Gain | cg13854874 | 21 | 37757525 |  | Island | NM_005441 | TSS200 | CHAF1B | 4.85E-09 | 2.12E-03 | 0.49 |
| Gain | cg12983839 | 19 | 2334615 |  | Island | NM_001077238;NM_152988 | Body;Body | SPPL2B;SPPL2B | 4.91E-09 | 2.15E-03 | 0.49 |
| Gain | cg12639558 | 12 | 121958832 |  |  | NM_032590;NM_001005366 | Body;Body | KDM2B;KDM2B | 4.94E-09 | 2.16E-03 | 0.50 |
| Gain | cg16541931 | 10 | 25463757 |  | Island | NR_027333;NM_020752 | Body;TSS1500 | LOC100128811;GPR158 | 4.97E-09 | 2.18E-03 | 0.49 |
| Gain | cg04624363 | 5 | 176433907 |  | Island | NM_016290 | TSS1500 | UIMC1 | 4.99E-09 | 2.19E-03 | 0.48 |
| Gain | cg04870212 | 1 | 6464845 |  |  |  |  |  | 5.05E-09 | 2.21E-03 | 0.50 |
| Gain | cg06638966 | 6 | 70576321 |  | N_Shore | NM_001858 | TSS200 | COL19A1 | 5.07E-09 | 2.22E-03 | 0.50 |
| Gain | cg09202227 | 16 | 68482715 |  | Island | NM_018667 | TSS1500 | SMPD3 | 5.08E-09 | 2.22E-03 | 0.50 |
| Gain | cg09773586 | 12 | 46778276 |  | S_Shore |  |  |  | 5.15E-09 | 2.25E-03 | 0.49 |
| Gain | cg00878605 | 19 | 17716645 | TRUE | Island | NM_001080421 | 3'UTR | UNC13A | 5.16E-09 | 2.26E-03 | 0.50 |
| Gain | cg02997982 | 19 | 41082291 |  | N_Shore | NM_138392;NM_020971 | TSS1500;3'UTR | SHKBP1;SPTBN4 | 5.18E-09 | 2.27E-03 | 0.50 |
| Gain | cg05651778 | 17 | 60729731 |  | Island | NM_006039 | Body | MRC2 | 5.26E-09 | 2.30E-03 | 0.50 |
| Gain | cg03554283 | 8 | 76319451 |  | Island |  |  |  | 5.29E-09 | 2.31E-03 | 0.50 |
| Gain | cg25987194 | 7 | 107301566 |  | Island | NM_000441;NR_028137 | 5'UTR;Body | SLC26A4;LOC286002 | 5.34E-09 | 2.34E-03 | 0.50 |
| Gain | cg19904265 | 1 | 3166712 |  | S_Shelf | NM_022114;NM_199454 | Body;Body | PRDM16;PRDM16 | 5.39E-09 | 2.36E-03 | 0.50 |
| Gain | cg08342886 | 6 | 33240066 |  | Island | NM_022553;NM_022551 | TSS1500;Body | VPS52;RPS18 | 5.40E-09 | 2.36E-03 | 0.47 |
| Gain | cg06142537 | 2 | 71114785 | TRUE | Island |  |  |  | 5.40E-09 | 2.36E-03 | 0.50 |
| Gain | cg16959606 | 1 | 169074934 |  | Island | NM_001677;NM_001001787 | TSS1500;TSS1500 | ATP1B1;ATP1B1 | 5.42E-09 | 2.37E-03 | 0.50 |
| Gain | cg15425280 | 4 | 158141492 |  | Island | NM_001083620;NM_001083619;NM_000826 | TSS1500;TSS1500;TSS1500 | GRIA2;GRIA2;GRIA2 | 5.48E-09 | 2.40E-03 | 0.49 |
| Gain | cg11138362 | 1 | 3239992 |  | Island | NM_022114;NM_199454 | Body;Body | PRDM16;PRDM16 | 5.49E-09 | 2.40E-03 | 0.49 |
| Loss | cg12737588 | 15 | 70546706 |  |  |  |  |  | 5.51E-09 | 2.41E-03 | -0.46 |
| Loss | cg14956327 | 6 | 110737053 |  |  | NM_004032;NM_003649 | TSS1500;TSS1500 | DDO;DDO | 5.53E-09 | 2.42E-03 | -0.50 |
| Gain | cg15778457 | 7 | 155165295 | TRUE | Island |  |  |  | 5.53E-09 | 2.42E-03 | 0.48 |
| Gain | cg26118408 | 7 | 126987840 | TRUE | Island |  |  |  | 5.74E-09 | 2.51E-03 | 0.49 |
| Gain | cg04925385 | 13 | 52378555 |  | Island | NM_001031719;NM_024705 | TSS1500;TSS1500 | DHRS12;DHRS12 | 5.76E-09 | 2.52E-03 | 0.48 |
| Gain | cg09984392 | 8 | 126011784 |  | S_Shore | NM_003129 | 1stExon | SQLE | 5.82E-09 | 2.55E-03 | 0.49 |
| Gain | cg07224469 | 4 | 716282 |  | Island | NM_006315 | 5'UTR | PCGF3 | 5.84E-09 | 2.56E-03 | 0.49 |
| Gain | cg18622870 | 11 | 61335113 |  | Island | NM_004200 | Body | SYT7 | 5.93E-09 | 2.59E-03 | 0.49 |
| Gain | cg00471645 | 2 | 11270192 |  |  |  |  |  | 5.96E-09 | 2.61E-03 | 0.48 |
| Loss | cg13306815 | 6 | 140364611 | TRUE |  |  |  |  | 5.98E-09 | 2.62E-03 | -0.50 |
| Gain | cg02865822 | 11 | 46383141 |  |  | NM_201533;NM_001105540;NM_003646;NM_201532 | Body;TSS200;Body;Body | DGKZ;DGKZ;DGKZ;DGKZ | 6.05E-09 | 2.65E-03 | 0.49 |
| Gain | cg17878972 | 3 | 192959356 |  | Island | NM_020386;NR_026877 | 5'UTR;TSS1500 | HRASLS;MGC2889 | 6.13E-09 | 2.68E-03 | 0.49 |
| Gain | cg23483765 | 5 | 156886996 |  | Island | NM_001099287 | TSS200 | NIPAL4 | 6.15E-09 | 2.69E-03 | 0.49 |
| Gain | cg04309212 | 2 | 223183932 |  | Island |  |  |  | 6.23E-09 | 2.73E-03 | 0.49 |
| Gain | cg02118776 | 6 | 44281222 |  | S_Shore | NM_020745 | TSS200 | AARS2 | 6.27E-09 | 2.74E-03 | 0.49 |
| Gain | cg07927379 | 7 | 156433108 |  | Island | NR_026865;NM_030936 | Body;TSS1500 | C7orf13;RNF32 | 6.32E-09 | 2.77E-03 | 0.49 |
| Gain | cg05540369 | 16 | 3355553 |  | Island | NM_153028;NM_153028 | 1stExon;5'UTR | ZNF75A;ZNF75A | 6.41E-09 | 2.81E-03 | 0.49 |
| Gain | cg21183502 | 20 | 58514581 |  | Island | NM_022106;NM_006242 | TSS1500;1stExon | C20orf177;PPP1R3D | 6.43E-09 | 2.81E-03 | 0.50 |
| Gain | cg26796095 | 19 | 5625046 |  | S_Shore | NM_002967 | Body | SAFB | 6.43E-09 | 2.81E-03 | 0.49 |
| Gain | cg26290632 | 8 | 91094847 |  |  | NM_004929 | 1stExon | CALB1 | 6.49E-09 | 2.84E-03 | 0.48 |
| Gain | cg27281285 | 12 | 120315368 |  | S_Shore | NM_007174 | TSS1500 | CIT | 6.52E-09 | 2.86E-03 | 0.48 |
| Gain | cg02851087 | 10 | 31422710 | TRUE | Island |  |  |  | 6.64E-09 | 2.91E-03 | 0.49 |
| Gain | cg05965414 | 2 | 217236799 |  | Island | NM_020814 | TSS200 | 4-Mar | 6.66E-09 | 2.91E-03 | 0.49 |
| Gain | cg03065467 | 4 | 109093243 | TRUE | Island | NR_029373;NR_029374 | TSS200;Body | LOC641518;LOC641518 | 6.71E-09 | 2.94E-03 | 0.50 |
| Gain | cg22235877 | 17 | 79791587 |  | S_Shore | NM_001093767;NM_207368;NM_001007533 | TSS1500;TSS1500;3'UTR | FAM195B;FAM195B;DYSFIP1 | 6.80E-09 | 2.98E-03 | 0.48 |
| Gain | cg04738827 | 2 | 85839650 |  | S_Shore | NM_001013649 | TSS1500 | C2orf68 | 6.85E-09 | 3.00E-03 | 0.50 |
| Gain | cg16717122 | 15 | 51973920 |  | S_Shore | NM_001165257;NM_013243;NM_013243;NM_001165257 | 5'UTR;1stExon;5'UTR;1stExon | SCG3;SCG3;SCG3;SCG3 | 6.86E-09 | 3.00E-03 | 0.50 |
| Gain | cg10696191 | 21 | 18985668 |  | Island | NM_001130914;NM_006806 | TSS1500;TSS1500 | BTG3;BTG3 | 6.88E-09 | 3.01E-03 | 0.50 |
| Gain | cg01561864 | 7 | 2679067 | TRUE |  | NM_025250 | Body | TTYH3 | 6.91E-09 | 3.03E-03 | 0.48 |
| Gain | cg03688818 | 3 | 49711147 |  | Island | NM_001640 | TSS1500 | APEH | 6.96E-09 | 3.05E-03 | 0.49 |
| Gain | cg09022993 | 4 | 52917875 |  | Island | NM_145263 | 1stExon | SPATA18 | 7.02E-09 | 3.07E-03 | 0.50 |
| Gain | cg21725716 | 19 | 1725535 |  | Island |  |  |  | 7.04E-09 | 3.08E-03 | 0.49 |
| Gain | cg04436083 | 4 | 24567220 | TRUE |  | NM_001358 | Body | DHX15 | 7.14E-09 | 3.12E-03 | 0.50 |
| Gain | cg24016624 | 19 | 45504513 |  | N_Shore | NM_006509 | TSS200 | RELB | 7.19E-09 | 3.15E-03 | 0.50 |
| Gain | cg09853371 | 4 | 57522145 |  | Island | NM_139212;NM_139211;NM_032495;NM_001145460;NM_001145459 | Body;Body;Body;Body;Body | HOPX;HOPX;HOPX;HOPX;HOPX | 7.20E-09 | 3.15E-03 | 0.50 |
| Gain | cg16465768 | 2 | 136287945 |  | N_Shore | NM_015361;NM_032143 | TSS1500;5'UTR | R3HDM1;ZRANB3 | 7.28E-09 | 3.19E-03 | 0.49 |
| Gain | cg10129041 | 2 | 129494526 |  | Island |  |  |  | 7.32E-09 | 3.20E-03 | 0.49 |
| Gain | cg08687825 | 6 | 84221752 |  |  | NM_153362;NM_001170423 | TSS1500;TSS1500 | PRSS35;PRSS35 | 7.42E-09 | 3.25E-03 | 0.50 |
| Gain | cg23244398 | 17 | 46756001 |  | Island |  |  |  | 7.54E-09 | 3.30E-03 | 0.49 |
| Gain | cg26546105 | 7 | 100168143 | TRUE | Island |  |  |  | 7.63E-09 | 3.34E-03 | 0.49 |
| Gain | cg24607283 | 3 | 11302249 |  |  | NM_001098213;NM_001098211;NM_001098212;NM_000861 | 3'UTR;3'UTR;3'UTR;3'UTR | HRH1;HRH1;HRH1;HRH1 | 7.77E-09 | 3.40E-03 | 0.48 |
| Gain | cg16603784 | 1 | 109756353 |  | N_Shore | NM_006513 | TSS200 | SARS | 7.85E-09 | 3.44E-03 | 0.49 |
| Loss | cg22450406 | 2 | 235422165 | TRUE |  |  |  |  | 7.85E-09 | 3.44E-03 | -0.49 |
| Gain | cg05886574 | 10 | 27530091 |  | Island | NM_145698;NM_001042473;NR_024150 | TSS1500;5'UTR;Body | ACBD5;ACBD5;ACBD5 | 7.89E-09 | 3.45E-03 | 0.50 |
| Gain | cg05725804 | 5 | 114516219 |  | Island | NM_001017397;NM_001017398;NM_018700;NM_018700;NM_001017397;NM_001017398 | 1stExon;1stExon;1stExon;5'UTR;5'UTR;5'UTR | TRIM36;TRIM36;TRIM36;TRIM36;TRIM36;TRIM36 | 7.91E-09 | 3.46E-03 | 0.49 |
| Gain | cg25112291 | 18 | 56936699 |  | Island | NM_013435 | Body | RAX | 8.04E-09 | 3.52E-03 | 0.49 |
| Gain | cg27645517 | 15 | 98971383 | TRUE | N_Shore |  |  |  | 8.07E-09 | 3.53E-03 | 0.49 |
| Gain | cg19281363 | 13 | 42031291 |  | Island | NM_014059 | TSS1500 | C13orf15 | 8.39E-09 | 3.67E-03 | 0.49 |
| Gain | cg02442436 | 9 | 112402882 | TRUE | Island | NM_001037293;NM_053016 | TSS200;TSS200 | PALM2;PALM2 | 8.41E-09 | 3.68E-03 | 0.49 |
| Gain | cg10193817 | 11 | 115375226 |  | Island | NM_001098517;NM_014333;NM_014333;NM_001098517 | 5'UTR;5'UTR;1stExon;1stExon | CADM1;CADM1;CADM1;CADM1 | 8.56E-09 | 3.75E-03 | 0.49 |
| Loss | cg00573165 | 1 | 20250849 |  |  | NM_014589 | TSS1500 | PLA2G2E | 8.59E-09 | 3.76E-03 | -0.49 |
| Gain | cg08881796 | 17 | 618545 |  | Island | NM_001128159;NM_018289 | TSS1500;TSS1500 | VPS53;VPS53 | 8.68E-09 | 3.80E-03 | 0.49 |
| Gain | cg14927724 | 8 | 41559608 | TRUE | Island | NM_020476;NM_020475;NM_001142446;NM_020477;NM_000037 | Body;Body;Body;Body;Body | ANK1;ANK1;ANK1;ANK1;ANK1 | 8.77E-09 | 3.84E-03 | 0.50 |
| Gain | cg01616356 | 22 | 50895351 |  | N_Shelf | NM_002972 | Body | SBF1 | 8.84E-09 | 3.87E-03 | 0.45 |
| Gain | cg16192371 | 6 | 31105337 |  |  | NM_014068;NM_014069 | Body;3'UTR | PSORS1C1;PSORS1C2 | 8.87E-09 | 3.88E-03 | 0.48 |
| Gain | cg18395636 | 11 | 87908785 |  | S_Shore | NM_022337 | TSS200 | RAB38 | 8.90E-09 | 3.90E-03 | 0.49 |
| Gain | cg07180355 | 1 | 16264490 |  | S_Shelf | NM_015001 | Body | SPEN | 8.95E-09 | 3.92E-03 | 0.48 |
| Gain | cg04769392 | 6 | 80579458 |  |  |  |  |  | 8.96E-09 | 3.92E-03 | 0.49 |
| Gain | cg14484688 | 9 | 131669841 |  | N_Shore | NM_001127245;NM_001127244;NM_019594 | Body;Body;Body | LRRC8A;LRRC8A;LRRC8A | 9.00E-09 | 3.94E-03 | 0.49 |
| Gain | cg19150852 | 3 | 43732102 |  | Island | NM_016006 | TSS1500 | ABHD5 | 9.03E-09 | 3.95E-03 | 0.48 |
| Gain | cg21105175 | 19 | 5917314 |  | S_Shelf | NM_003624;NM_007322;NM_007320 | 3'UTR;3'UTR;3'UTR | RANBP3;RANBP3;RANBP3 | 9.12E-09 | 3.99E-03 | 0.49 |
| Gain | cg05630556 | 5 | 94619419 |  | N_Shore | NM_024717 | Body | MCTP1 | 9.16E-09 | 4.01E-03 | 0.49 |
| Gain | cg17576288 | 13 | 79177877 |  | Island | NM_006237 | TSS200 | POU4F1 | 9.19E-09 | 4.02E-03 | 0.50 |
| Gain | cg02980971 | 16 | 83841413 |  | Island | NM_001537 | TSS200 | HSBP1 | 9.21E-09 | 4.03E-03 | 0.48 |
| Gain | cg07447260 | 10 | 103600544 | TRUE | N_Shelf | NM_173195;NM_173191;NM_173194;NM_173193;NM_173192;NM_014591;NM_173197 | Body;Body;TSS1500;Body;Body;Body;Body | KCNIP2;KCNIP2;KCNIP2;KCNIP2;KCNIP2;KCNIP2;KCNIP2 | 9.26E-09 | 4.05E-03 | 0.47 |
| Gain | cg12584718 | 7 | 100028402 |  | S_Shore | NM_019606 | 1stExon | MEPCE | 9.27E-09 | 4.06E-03 | 0.47 |
| Gain | cg01144053 | 2 | 7571361 |  | Island |  |  |  | 9.36E-09 | 4.10E-03 | 0.48 |
| Gain | cg25108022 | 5 | 172199313 |  | Island | NM_004417 | TSS1500 | DUSP1 | 9.39E-09 | 4.11E-03 | 0.48 |
| Gain | cg11204099 | 12 | 22777820 |  | N_Shore | NM_018638;NM_001039481 | TSS1500;TSS1500 | ETNK1;ETNK1 | 9.43E-09 | 4.13E-03 | 0.48 |
| Loss | cg20389635 | 12 | 28120054 |  | N_Shelf | NM_198964;NM_198965;NM_198966;NM_002820 | Body;Body;Body;Body | PTHLH;PTHLH;PTHLH;PTHLH | 9.56E-09 | 4.18E-03 | -0.50 |
| Gain | cg16956501 | 13 | 50070520 |  | Island | NM_001040444;NM_001040443 | TSS200;Body | PHF11;PHF11 | 9.73E-09 | 4.26E-03 | 0.48 |
| Gain | cg21724796 | 17 | 8113904 |  | Island | NM_004217 | TSS200 | AURKB | 1.00E-08 | 4.39E-03 | 0.48 |
| Gain | cg21555177 | 6 | 33173024 |  | S_Shore | NM_014234 | Body | HSD17B8 | 1.00E-08 | 4.39E-03 | 0.49 |
| Gain | cg25104186 | 1 | 34098967 |  | Island | NM_052896 | Body | CSMD2 | 1.02E-08 | 4.47E-03 | 0.47 |
| Gain | cg01867395 | 11 | 31839628 |  | Island | NM_001127612 | TSS200 | PAX6 | 1.02E-08 | 4.48E-03 | 0.50 |
| Loss | cg17724147 | 4 | 58290751 |  |  |  |  |  | 1.03E-08 | 4.50E-03 | -0.49 |
| Gain | cg16321523 | 8 | 82633764 |  | S_Shore | NR_033194;NR_033195;NM_001170796;NR_033196;NM_024699;NR_033193;NM_001170797 | TSS1500;TSS1500;TSS1500;TSS1500;TSS1500;TSS1500;TSS1500 | ZFAND1;ZFAND1;ZFAND1;ZFAND1;ZFAND1;ZFAND1;ZFAND1 | 1.04E-08 | 4.53E-03 | 0.49 |
| Gain | cg21431125 | 1 | 41131487 |  | S_Shore | NM_014747 | TSS200 | RIMS3 | 1.04E-08 | 4.54E-03 | 0.48 |
| Gain | cg25090510 | 5 | 156886990 |  | Island | NM_001099287 | TSS200 | NIPAL4 | 1.05E-08 | 4.60E-03 | 0.47 |
| Gain | cg07193234 | 15 | 28341783 |  | Island | NM_000275 | 5'UTR | OCA2 | 1.06E-08 | 4.65E-03 | 0.49 |
| Gain | cg24229963 | 8 | 82633648 |  | Island | NM_001170797;NM_001170796;NR_033194;NM_024699;NR_033195;NR_033196;NR_033193 | TSS200;TSS200;TSS200;TSS200;TSS200;TSS200;TSS200 | ZFAND1;ZFAND1;ZFAND1;ZFAND1;ZFAND1;ZFAND1;ZFAND1 | 1.08E-08 | 4.72E-03 | 0.48 |
| Gain | cg00870662 | 18 | 78005665 |  | S_Shore | NM_032510 | TSS1500 | PARD6G | 1.08E-08 | 4.73E-03 | 0.49 |
| Gain | cg22186515 | 6 | 106533997 |  | N_Shore | NM_001198 | TSS200 | PRDM1 | 1.08E-08 | 4.74E-03 | 0.49 |
| Gain | cg26545313 | 4 | 3417677 |  | S_Shelf | NM_198229;NM_198227;NM_002926 | Body;Body;Body | RGS12;RGS12;RGS12 | 1.08E-08 | 4.74E-03 | 0.48 |
| Gain | cg16430166 | 1 | 232941468 | TRUE | Island | NM_019090 | 1stExon | KIAA1383 | 1.09E-08 | 4.75E-03 | 0.48 |
| Gain | cg02722188 | 4 | 55099058 |  | Island | NM_006206 | 5'UTR | PDGFRA | 1.10E-08 | 4.82E-03 | 0.49 |
| Gain | cg06283270 | 15 | 93353066 |  | Island |  |  |  | 1.11E-08 | 4.84E-03 | 0.47 |
| Gain | cg02456288 | 14 | 38053308 | TRUE | Island |  |  |  | 1.11E-08 | 4.86E-03 | 0.49 |
| Gain | cg13744663 | 13 | 92050675 |  | N_Shore | NM_004466 | TSS1500 | GPC5 | 1.12E-08 | 4.90E-03 | 0.49 |
| Gain | cg07594674 | 2 | 47499812 |  | Island |  |  |  | 1.13E-08 | 4.96E-03 | 0.48 |
| Gain | cg10164300 | 11 | 123986034 | TRUE |  | NM_198315;NM_001130142;NM_014622 | TSS200;TSS200;TSS200 | VWA5A;VWA5A;VWA5A | 1.14E-08 | 4.98E-03 | 0.49 |
| Gain | cg25380622 | 5 | 150695684 |  |  | NM_181776 | 3'UTR | SLC36A2 | 1.14E-08 | 4.99E-03 | 0.48 |
| Gain | cg08484671 | 19 | 41055297 |  | Island | NM_025213;NM_020971 | Body;Body | SPTBN4;SPTBN4 | 1.14E-08 | 4.99E-03 | 0.47 |
| Gain | cg09364688 | 1 | 36038877 | TRUE | N_Shore | NM_178548 | TSS200 | TFAP2E | 1.14E-08 | 5.01E-03 | 0.47 |
| Gain | cg22624255 | 19 | 19779476 |  | Island | NM_033204 | TSS200 | ZNF101 | 1.15E-08 | 5.02E-03 | 0.49 |
| Gain | cg03777575 | 11 | 62413296 |  | N_Shore | NM_198335;NM_198334 | Body;Body | GANAB;GANAB | 1.15E-08 | 5.02E-03 | 0.49 |
| Gain | cg10593047 | 2 | 232526667 |  | Island |  |  |  | 1.15E-08 | 5.05E-03 | 0.47 |
| Gain | cg02662658 | 17 | 34257571 | TRUE | N_Shore | NM_001163125;NR_027997;NR_027998;NR_027999;NM_145654;NM_001163120;NM_001163122;NM_00116 | TSS1500;Body;Body;TSS1500;Body;Body;TSS1500;Body;TSS1500;TSS1500;TSS1500;Body;Body | RDM1;RDM1;RDM1;RDM1;RDM1;RDM1;RDM1;RDM1;RDM1;RDM1;RDM1;RDM1;RDM1 | 1.17E-08 | 5.10E-03 | 0.49 |
| Gain | cg14315058 | 8 | 99182422 |  |  |  |  |  | 1.18E-08 | 5.15E-03 | 0.48 |
| Gain | cg02732509 | 12 | 58241200 |  | S_Shore | NM_005730 | TSS1500 | CTDSP2 | 1.19E-08 | 5.20E-03 | 0.48 |
| Gain | cg18691434 | 7 | 99775425 |  | Island | NM_012447;NM_152742 | TSS200;TSS1500 | STAG3;GPC2 | 1.20E-08 | 5.27E-03 | 0.49 |
| Loss | cg21723861 | 17 | 39686628 |  | S_Shelf |  |  |  | 1.21E-08 | 5.28E-03 | -0.49 |
| Gain | cg23384185 | 18 | 657227 |  | N_Shore | NM_001012716;NM_001071 | 3'UTR;TSS1500 | C18orf56;TYMS | 1.21E-08 | 5.29E-03 | 0.49 |
| Gain | cg05846166 | 16 | 2770814 |  | N_Shore | NM_031948 | TSS1500 | PRSS27 | 1.24E-08 | 5.44E-03 | 0.48 |
| Gain | cg24550865 | 3 | 190040375 | TRUE | Island | NM_021101 | TSS200 | CLDN1 | 1.27E-08 | 5.56E-03 | 0.48 |
| Gain | cg20308540 | 1 | 242612849 |  |  | NM_152666 | 5'UTR | PLD5 | 1.28E-08 | 5.60E-03 | 0.47 |
| Gain | cg03507326 | 16 | 2801952 |  | Island | NR_027275;NM_016333 | Body;TSS1500 | LOC100128788;SRRM2 | 1.28E-08 | 5.62E-03 | 0.49 |
| Gain | cg25778262 | 12 | 69327449 |  | Island | NM_198320;NM_001005502;NM_001874 | TSS1500;TSS1500;5'UTR | CPM;CPM;CPM | 1.29E-08 | 5.65E-03 | 0.49 |
| Gain | cg26377000 | 17 | 33814891 | TRUE | Island | NM_001145027 | Body | SLFN12L | 1.29E-08 | 5.66E-03 | 0.49 |
| Gain | cg03799530 | 12 | 111843215 |  | Island | NM_005475 | TSS1500 | SH2B3 | 1.34E-08 | 5.88E-03 | 0.48 |
| Gain | cg05208878 | 22 | 22901596 |  | Island | NM_206956;NM_006115;NM_206954;NM_006115;NR_027426;NM_206955;NM_206953;NM_206954;NM_2069 | TSS200;5'UTR;1stExon;1stExon;TSS200;TSS200;5'UTR;5'UTR;1stExon | PRAME;PRAME;PRAME;PRAME;LOC648691;PRAME;PRAME;PRAME;PRAME | 1.34E-08 | 5.89E-03 | 0.49 |
| Gain | cg24948962 | 18 | 2906032 |  | Island | NM_032048 | Body | EMILIN2 | 1.36E-08 | 5.93E-03 | 0.47 |
| Gain | cg00708380 | 11 | 75378502 |  | Island | NM_033063;NM_207577 | Body;Body | MAP6;MAP6 | 1.36E-08 | 5.97E-03 | 0.48 |
| Gain | cg00350942 | 9 | 140034073 |  | Island | NM_021569;NM_007327;NM_000832 | 1stExon;1stExon;1stExon | GRIN1;GRIN1;GRIN1 | 1.37E-08 | 6.02E-03 | 0.46 |
| Gain | cg25061843 | 9 | 1042970 |  | Island |  |  |  | 1.39E-08 | 6.07E-03 | 0.48 |
| Gain | cg17497271 | 15 | 40212781 |  | Island | NM_007223;NM_007223 | 5'UTR;1stExon | GPR176;GPR176 | 1.40E-08 | 6.12E-03 | 0.48 |
| Gain | cg08153345 | 12 | 2862084 |  | Island |  |  |  | 1.41E-08 | 6.19E-03 | 0.47 |
| Loss | cg05471495 | 2 | 101369330 | TRUE |  |  |  |  | 1.41E-08 | 6.19E-03 | -0.48 |
| Gain | cg05290394 | 12 | 32552899 | TRUE | Island |  |  |  | 1.43E-08 | 6.26E-03 | 0.49 |
| Gain | cg12209165 | 5 | 132073546 |  | S_Shore | NM_007054 | TSS1500 | KIF3A | 1.43E-08 | 6.27E-03 | 0.48 |
| Gain | cg20863756 | 12 | 6809797 |  | Island | NM_153685;NM_153685 | 5'UTR;1stExon | C12orf53;C12orf53 | 1.44E-08 | 6.31E-03 | 0.48 |
| Gain | cg10363337 | 8 | 144891927 |  | Island | NM_015356;NM_182706 | Body;Body | SCRIB;SCRIB | 1.46E-08 | 6.40E-03 | 0.49 |
| Gain | cg00237391 | 6 | 35265643 | TRUE |  | NM_022047 | 1stExon | DEF6 | 1.47E-08 | 6.45E-03 | 0.48 |
| Gain | cg09679923 | 17 | 36103036 |  | Island | NM_000458;NM_001165923 | Body;Body | HNF1B;HNF1B | 1.49E-08 | 6.53E-03 | 0.47 |
| Gain | cg12669395 | 6 | 30860866 |  |  | NM_013993;NM_001954;NM_013994 | Body;Body;Body | DDR1;DDR1;DDR1 | 1.49E-08 | 6.54E-03 | 0.47 |
| Gain | cg13378934 | 19 | 47910580 | TRUE | S_Shore | NM_001009813;NM_020160 | Body;Body | MEIS3;MEIS3 | 1.50E-08 | 6.55E-03 | 0.49 |
| Gain | cg07798694 | 4 | 142141972 |  | Island | NM_014487 | TSS200 | ZNF330 | 1.50E-08 | 6.56E-03 | 0.48 |
| Gain | cg15611624 | 5 | 156886970 |  | Island | NM_001099287 | TSS200 | NIPAL4 | 1.52E-08 | 6.67E-03 | 0.46 |
| Gain | cg06718763 | 1 | 24828586 |  | N_Shore | NM_013441 | TSS1500 | RCAN3 | 1.53E-08 | 6.68E-03 | 0.49 |
| Gain | cg13699808 | 20 | 45985339 |  |  | NM_183048;NM_183047;NM_012408 | Body;Body;Body | ZMYND8;ZMYND8;ZMYND8 | 1.56E-08 | 6.82E-03 | 0.49 |
| Gain | cg03490115 | 2 | 74681972 |  | Island | NM_031288 | TSS1500 | INO80B | 1.57E-08 | 6.85E-03 | 0.47 |
| Gain | cg00754688 | 2 | 225307499 |  | Island |  |  |  | 1.58E-08 | 6.91E-03 | 0.48 |
| Gain | cg18236477 | 13 | 26043066 |  | Island | NM_016529 | Body | ATP8A2 | 1.61E-08 | 7.06E-03 | 0.48 |
| Gain | cg07060551 | 19 | 51198381 |  | Island | NM_016148 | Body | SHANK1 | 1.62E-08 | 7.08E-03 | 0.48 |
| Gain | cg11967546 | 6 | 27655170 |  | N_Shore |  |  |  | 1.63E-08 | 7.12E-03 | 0.48 |
| Gain | cg08578136 | 2 | 207139443 |  | Island | NM_020923 | TSS200 | ZDBF2 | 1.63E-08 | 7.13E-03 | 0.49 |
| Gain | cg11601932 | 3 | 195578011 |  |  |  |  |  | 1.63E-08 | 7.14E-03 | 0.48 |
| Gain | cg17152981 | 6 | 110299835 | TRUE | Island | NM_005284 | TSS1500 | GPR6 | 1.64E-08 | 7.18E-03 | 0.47 |
| Gain | cg04577625 | 1 | 111506380 |  | Island | NM_001006945;NM_018372;NM_001006945;NM_018372 | 1stExon;5'UTR;5'UTR;1stExon | C1orf103;C1orf103;C1orf103;C1orf103 | 1.65E-08 | 7.22E-03 | 0.48 |
| Gain | cg01763090 | 15 | 31775406 |  | N_Shore | NM_130901 | 3'UTR | OTUD7A | 1.65E-08 | 7.24E-03 | 0.49 |
| Gain | cg08243849 | 1 | 31539007 |  | S_Shore | NM_001020658;NM_014676 | TSS1500;TSS1500 | PUM1;PUM1 | 1.66E-08 | 7.26E-03 | 0.48 |
| Gain | cg03539717 | 19 | 13065086 | TRUE | N_Shelf | NM_052850 | Body | GADD45GIP1 | 1.71E-08 | 7.50E-03 | 0.49 |
| Gain | cg18055623 | 19 | 54485327 |  | Island | NM_031895;NR_030632 | Body;TSS1500 | CACNG8;MIR935 | 1.72E-08 | 7.53E-03 | 0.46 |
| Gain | cg06741803 | 1 | 46807522 |  | S_Shore | NM_199044 | Body | NSUN4 | 1.72E-08 | 7.53E-03 | 0.47 |
| Gain | cg05548912 | 17 | 40464318 |  | Island |  |  |  | 1.72E-08 | 7.53E-03 | 0.48 |
| Gain | cg22930390 | 19 | 911815 |  | Island | NM_138774 | Body | C19orf22 | 1.73E-08 | 7.58E-03 | 0.48 |
| Gain | cg04875128 | 15 | 31775895 |  | Island | NM_130901 | Body | OTUD7A | 1.74E-08 | 7.61E-03 | 0.49 |
| Gain | cg24810473 | 5 | 139090314 |  | Island |  |  |  | 1.74E-08 | 7.63E-03 | 0.48 |
| Gain | cg17421143 | 11 | 43604527 | TRUE | S_Shore |  |  |  | 1.75E-08 | 7.64E-03 | 0.48 |
| Gain | cg00303541 | 3 | 51741280 | TRUE | Island | NM_000839;NM_001130063 | 5'UTR;5'UTR | GRM2;GRM2 | 1.75E-08 | 7.67E-03 | 0.46 |
| Gain | cg24051481 | 13 | 95364062 |  | Island | NM_007084 | 1stExon | SOX21 | 1.76E-08 | 7.69E-03 | 0.49 |
| Gain | cg22642485 | 5 | 139089846 |  | Island |  |  |  | 1.78E-08 | 7.80E-03 | 0.46 |
| Gain | cg19558029 | 18 | 44526605 | TRUE | N_Shore | NM_031303 | TSS200 | KATNAL2 | 1.80E-08 | 7.86E-03 | 0.49 |
| Gain | cg18143296 | 3 | 157812763 |  | Island |  |  |  | 1.81E-08 | 7.91E-03 | 0.49 |
| Gain | cg20275507 | 6 | 70576315 |  | N_Shore | NM_001858 | TSS200 | COL19A1 | 1.81E-08 | 7.93E-03 | 0.49 |
| Gain | cg01984858 | 6 | 28555079 | TRUE | Island | NM_052923;NM_052923 | 1stExon;5'UTR | SCAND3;SCAND3 | 1.83E-08 | 7.99E-03 | 0.48 |
| Gain | cg17674725 | 2 | 133426418 |  | N_Shore | NM_001077427;NM_144586 | 5'UTR;Body | LYPD1;LYPD1 | 1.83E-08 | 8.02E-03 | 0.47 |
| Gain | cg25139493 | 1 | 39957400 |  | Island | NM_181809;NM_181809 | 1stExon;5'UTR | BMP8A;BMP8A | 1.84E-08 | 8.07E-03 | 0.48 |
| Gain | cg01410314 | 9 | 77641437 |  | N_Shore | NM_152420 | Body | C9orf41 | 1.85E-08 | 8.10E-03 | 0.46 |
| Gain | cg01534416 | 19 | 51308546 |  | S_Shore | NM_199250;NM_199249 | TSS1500;TSS1500 | C19orf48;C19orf48 | 1.86E-08 | 8.13E-03 | 0.46 |
| Gain | cg17694795 | 8 | 67874366 | TRUE | Island |  |  |  | 1.86E-08 | 8.14E-03 | 0.49 |
| Gain | cg03397716 | 1 | 6484980 |  | Island | NM_031475;NM_031475 | 1stExon;5'UTR | ESPN;ESPN | 1.86E-08 | 8.16E-03 | 0.46 |
| Gain | cg08078694 | 8 | 48100179 |  | Island |  |  |  | 1.88E-08 | 8.22E-03 | 0.47 |
| Gain | cg06028917 | 3 | 59035407 |  | Island | NM_198463 | 5'UTR | C3orf67 | 1.89E-08 | 8.27E-03 | 0.48 |
| Gain | cg14519125 | 3 | 49893811 | TRUE | Island | NM_005879 | 1stExon | TRAIP | 1.91E-08 | 8.36E-03 | 0.49 |
| Gain | cg26596350 | 16 | 27931936 | TRUE |  | NM_001109763 | Body | GSG1L | 1.91E-08 | 8.36E-03 | 0.48 |
| Gain | cg01071511 | 7 | 2052474 | TRUE | N_Shore | NM_003550;NM_001013837;NM_001013836 | Body;Body;Body | MAD1L1;MAD1L1;MAD1L1 | 1.91E-08 | 8.38E-03 | 0.47 |
| Gain | cg23779890 | 8 | 75262522 |  | Island | NM_001040875;NM_018972 | TSS200;TSS200 | GDAP1;GDAP1 | 1.96E-08 | 8.58E-03 | 0.46 |
| Gain | cg15201877 | 1 | 71512973 | TRUE | Island | NM_198718;NR_028294;NM_198714;NR_028293;NM_198716;NM_198717;NR_028292;NM_001126044;NM_1 | 1stExon;Body;1stExon;Body;1stExon;1stExon;Body;1stExon;1stExon;1stExon | PTGER3;PTGER3;PTGER3;PTGER3;PTGER3;PTGER3;PTGER3;PTGER3;PTGER3;PTGER3 | 1.96E-08 | 8.58E-03 | 0.48 |
| Gain | cg22958571 | 5 | 57878526 | TRUE | N_Shore | NM_138453 | TSS1500 | RAB3C | 1.97E-08 | 8.61E-03 | 0.47 |
| Gain | cg03667051 | 2 | 7571641 |  | Island |  |  |  | 1.97E-08 | 8.62E-03 | 0.48 |
| Gain | cg17493727 | 6 | 108169872 | TRUE | S_Shore |  |  |  | 2.01E-08 | 8.79E-03 | 0.48 |
| Gain | cg25399461 | 17 | 80824633 | TRUE | N_Shelf | NM_005993 | Body | TBCD | 2.03E-08 | 8.88E-03 | 0.48 |
| Gain | cg11866674 | 7 | 22589544 | TRUE | S_Shore |  |  |  | 2.05E-08 | 8.96E-03 | 0.48 |
| Gain | cg01143804 | 4 | 40751845 |  | Island | NM_024677 | TSS200 | NSUN7 | 2.08E-08 | 9.09E-03 | 0.48 |
| Gain | cg08278892 | 19 | 55574619 |  | Island | NR_027382;NM_138412;NM_001145971;NR_027381 | TSS200;5'UTR;TSS200;TSS200 | RDH13;RDH13;RDH13;RDH13 | 2.08E-08 | 9.11E-03 | 0.48 |
| Gain | cg24526702 | 17 | 56833201 |  | Island | NM_014906 | TSS200 | PPM1E | 2.09E-08 | 9.14E-03 | 0.48 |
| Gain | cg06900404 | 20 | 54919037 |  | Island |  |  |  | 2.10E-08 | 9.17E-03 | 0.47 |
| Gain | cg23681664 | 3 | 9746637 | TRUE | Island | NM_153635 | Body | CPNE9 | 2.10E-08 | 9.18E-03 | 0.48 |
| Gain | cg07584077 | 2 | 240965270 |  | Island | NM_004544 | TSS1500 | NDUFA10 | 2.10E-08 | 9.21E-03 | 0.48 |
| Gain | cg09861346 | 5 | 176037510 |  | Island | NM_052899 | TSS1500 | GPRIN1 | 2.16E-08 | 9.47E-03 | 0.47 |
| Gain | cg08573435 | 7 | 1398116 |  |  |  |  |  | 2.17E-08 | 9.49E-03 | 0.48 |
| Gain | cg15777261 | 2 | 73462569 |  | S_Shore | NR_029402;NR_029403;NM_001009570;NM_006429;NM_001166284;NM_001166285 | Body;Body;Body;Body;Body;5'UTR | CCT7;CCT7;CCT7;CCT7;CCT7;CCT7 | 2.19E-08 | 9.59E-03 | 0.48 |
| Loss | cg22266824 | 4 | 159918056 | TRUE |  | NM_152543 | Body | C4orf45 | 2.20E-08 | 9.64E-03 | -0.48 |
| Gain | cg05342634 | 3 | 197807842 |  | Island | NR_003291 | TSS1500 | LOC348840 | 2.20E-08 | 9.65E-03 | 0.48 |
| Gain | cg09195550 | 21 | 45746856 |  | S_Shelf | NR_024108;NM_002626 | Body;3'UTR | PFKL;PFKL | 2.21E-08 | 9.68E-03 | 0.46 |
| Gain | cg24544803 | 6 | 29596840 |  | S_Shore | NM_021903;NM_021904;NM_001470 | TSS1500;Body;Body | GABBR1;GABBR1;GABBR1 | 2.25E-08 | 9.84E-03 | 0.48 |
| Gain | cg23981354 | 6 | 139455976 |  | N_Shore | NM_016217 | TSS1500 | HECA | 2.28E-08 | 9.99E-03 | 0.48 |
| Gain | cg25204094 | 9 | 100747473 |  | Island | NM_006401 | Body | ANP32B | 2.35E-08 | 1.03E-02 | 0.48 |
| Gain | cg18428193 | 2 | 27665017 |  | Island | NM_173853;NM_013392;NM_001168364 | TSS1500;3'UTR;TSS1500 | KRTCAP3;NRBP1;KRTCAP3 | 2.35E-08 | 1.03E-02 | 0.48 |
| Gain | cg26400491 | 8 | 144891915 |  | Island | NM_015356;NM_182706 | Body;Body | SCRIB;SCRIB | 2.38E-08 | 1.04E-02 | 0.48 |
| Gain | cg05173913 | 17 | 34091901 | TRUE | Island | NM_145272 | 3'UTR | C17orf50 | 2.40E-08 | 1.05E-02 | 0.48 |
| Gain | cg15201635 | 16 | 68482637 |  | Island | NM_018667 | TSS1500 | SMPD3 | 2.40E-08 | 1.05E-02 | 0.48 |
| Gain | cg20543544 | 10 | 81003657 |  | Island | NM_020338 | Body | ZMIZ1 | 2.41E-08 | 1.05E-02 | 0.48 |
| Gain | cg02447229 | 6 | 158957221 |  | Island | NM_020823 | TSS1500 | TMEM181 | 2.41E-08 | 1.06E-02 | 0.47 |
| Gain | cg00384539 | 8 | 70983567 |  | Island | NM_024504 | TSS200 | PRDM14 | 2.42E-08 | 1.06E-02 | 0.48 |
| Gain | cg04684152 | 2 | 72012863 |  | Island |  |  |  | 2.42E-08 | 1.06E-02 | 0.45 |
| Gain | cg12584450 | 19 | 1986191 |  | Island | NM_017797 | 3'UTR | BTBD2 | 2.44E-08 | 1.07E-02 | 0.47 |
| Gain | cg22437153 | 2 | 73110657 |  | N_Shelf |  |  |  | 2.45E-08 | 1.07E-02 | 0.48 |
| Gain | cg08209133 | 4 | 48485624 | TRUE | Island | NM_152679 | 1stExon | SLC10A4 | 2.46E-08 | 1.08E-02 | 0.48 |
| Gain | cg03801300 | 10 | 130010447 |  | S_Shore |  |  |  | 2.46E-08 | 1.08E-02 | 0.47 |
| Gain | cg20673481 | 2 | 18059454 |  | N_Shore | NM_002252 | TSS1500 | KCNS3 | 2.48E-08 | 1.08E-02 | 0.47 |
| Gain | cg04805619 | 3 | 167097928 |  | Island | NM_024687 | 5'UTR | ZBBX | 2.49E-08 | 1.09E-02 | 0.48 |
| Gain | cg08207660 | 14 | 71694874 | TRUE |  |  |  |  | 2.50E-08 | 1.09E-02 | 0.47 |
| Loss | cg27535538 | 7 | 105680155 | TRUE |  |  |  |  | 2.52E-08 | 1.10E-02 | -0.47 |
| Gain | cg23105471 | 12 | 58241215 |  | S_Shore | NM_005730 | TSS1500 | CTDSP2 | 2.55E-08 | 1.11E-02 | 0.47 |
| Gain | cg13793048 | 15 | 44092314 | TRUE | N_Shore | NM_001033517;NM_016400 | TSS200;TSS1500 | SERINC4;C15orf63 | 2.57E-08 | 1.13E-02 | 0.48 |
| Gain | cg09378441 | 7 | 36429182 |  | N_Shore | NM_018685;NM_001100425 | TSS1500;Body | ANLN;KIAA0895 | 2.58E-08 | 1.13E-02 | 0.47 |
| Gain | cg00094518 | 7 | 130418549 |  | Island | NM_138693 | 1stExon | KLF14 | 2.60E-08 | 1.14E-02 | 0.48 |
| Gain | cg22711679 | 1 | 28241425 |  | Island | NM_002946 | TSS200 | RPA2 | 2.62E-08 | 1.15E-02 | 0.47 |
| Gain | cg24621354 | 7 | 115850382 |  | Island | NM_015641 | TSS200 | TES | 2.63E-08 | 1.15E-02 | 0.45 |
| Gain | cg06095695 | 6 | 28831653 |  | N_Shore |  |  |  | 2.63E-08 | 1.15E-02 | 0.47 |
| Gain | cg19464016 | 6 | 106533958 |  | N_Shore | NM_001198 | TSS1500 | PRDM1 | 2.63E-08 | 1.15E-02 | 0.47 |
| Gain | cg07225598 | 1 | 228646916 |  | S_Shore | NM_033445 | TSS1500 | HIST3H2A | 2.68E-08 | 1.17E-02 | 0.48 |
| Gain | cg18795809 | 4 | 10458531 |  | Island | NM_053042 | 5'UTR | ZNF518B | 2.69E-08 | 1.18E-02 | 0.48 |
| Loss | cg05632420 | 7 | 130793732 |  | N_Shore | NM_001145354;NR_015431 | TSS1500;TSS200 | MKLN1;FLJ43663 | 2.69E-08 | 1.18E-02 | -0.47 |
| Gain | cg10999598 | 5 | 125936257 |  | N_Shore | NM_032177 | TSS1500 | PHAX | 2.71E-08 | 1.19E-02 | 0.47 |
| Gain | cg04653012 | 16 | 58163677 |  | Island | NM_013242 | TSS1500 | C16orf80 | 2.73E-08 | 1.19E-02 | 0.47 |
| Gain | cg15381304 | 6 | 110300099 | TRUE | Island | NM_005284 | TSS200 | GPR6 | 2.78E-08 | 1.22E-02 | 0.44 |
| Gain | cg07667161 | 6 | 42847125 |  |  | NM_198486 | TSS1500 | RPL7L1 | 2.83E-08 | 1.24E-02 | 0.48 |
| Gain | cg04777071 | 19 | 42463824 |  | S_Shore | NM_006423 | TSS1500 | RABAC1 | 2.84E-08 | 1.24E-02 | 0.47 |
| Gain | cg01839993 | 10 | 74034644 |  | Island | NM_019058 | Body | DDIT4 | 2.86E-08 | 1.25E-02 | 0.44 |
| Gain | cg25133192 | 1 | 182808143 |  | N_Shore | NM_001357 | TSS1500 | DHX9 | 2.90E-08 | 1.27E-02 | 0.46 |
| Gain | cg11970192 | 15 | 80215376 |  | Island | NR_028330;NM_001100880 | Body;5'UTR | C15orf37;ST20 | 2.90E-08 | 1.27E-02 | 0.46 |
| Loss | cg15703035 | 13 | 111522932 |  |  | NR_027701 | TSS1500 | C13orf29 | 2.91E-08 | 1.27E-02 | -0.47 |
| Gain | cg27335600 | 3 | 53528857 | TRUE | Island | NM_001128840;NM_001128839;NM_000720 | TSS200;TSS200;TSS200 | CACNA1D;CACNA1D;CACNA1D | 2.91E-08 | 1.28E-02 | 0.46 |
| Gain | cg04110283 | 11 | 17373108 |  | Island | NR_026750 | TSS1500 | DKFZp686O24166 | 2.93E-08 | 1.28E-02 | 0.47 |
| Gain | cg04100532 | 2 | 210636560 |  | Island | NM_182587;NM_032504 | TSS200;TSS200 | UNC80;UNC80 | 2.94E-08 | 1.29E-02 | 0.48 |
| Gain | cg00246386 | 20 | 2674548 |  | Island | NM_001110514 | Body | EBF4 | 2.96E-08 | 1.30E-02 | 0.48 |
| Gain | cg14940405 | 8 | 142318883 |  | Island |  |  |  | 2.98E-08 | 1.30E-02 | 0.48 |
| Gain | cg08220519 | 11 | 10562913 |  | Island | NM_016422 | TSS200 | RNF141 | 2.98E-08 | 1.30E-02 | 0.48 |
| Gain | cg09454892 | 10 | 76870850 |  | N_Shore | NM_144660 | TSS1500 | SAMD8 | 2.99E-08 | 1.31E-02 | 0.48 |
| Loss | cg10376827 | 10 | 88730324 | TRUE | N_Shore | NM_133447;NM_006829 | TSS200;Body | AGAP11;C10orf116 | 2.99E-08 | 1.31E-02 | -0.48 |
| Gain | cg11635304 | 5 | 180288855 |  | Island | NM_152283 | TSS1500 | ZFP62 | 2.99E-08 | 1.31E-02 | 0.48 |
| Gain | cg07984256 | 16 | 66304438 | TRUE | Island |  |  |  | 2.99E-08 | 1.31E-02 | 0.47 |
| Gain | cg18451114 | 1 | 38511677 |  | Island | NM_002699 | 1stExon | POU3F1 | 2.99E-08 | 1.31E-02 | 0.47 |
| Gain | cg24871691 | 12 | 3601494 |  | Island | NM_019854 | Body | PRMT8 | 3.01E-08 | 1.32E-02 | 0.47 |
| Gain | cg16010628 | 12 | 49692283 |  | S_Shore | NM_006262 | 3'UTR | PRPH | 3.02E-08 | 1.32E-02 | 0.47 |
| Gain | cg01687680 | 16 | 49312238 |  | Island | NM_004352 | 3'UTR | CBLN1 | 3.04E-08 | 1.33E-02 | 0.47 |
| Gain | cg09229918 | 16 | 2041916 |  | Island | NM_004209 | Body | SYNGR3 | 3.05E-08 | 1.33E-02 | 0.48 |
| Gain | cg07570470 | 8 | 142318841 |  | Island |  |  |  | 3.05E-08 | 1.34E-02 | 0.47 |
| Gain | cg24480120 | 17 | 7982693 |  | Island | NM_001139 | Body | ALOX12B | 3.08E-08 | 1.35E-02 | 0.48 |
| Gain | cg04121261 | 18 | 12038580 |  | Island |  |  |  | 3.09E-08 | 1.35E-02 | 0.47 |
| Gain | cg27209578 | 2 | 175193470 |  | Island |  |  |  | 3.10E-08 | 1.36E-02 | 0.48 |
| Gain | cg19005368 | 11 | 32851950 | TRUE | Island | NM_024081 | 5'UTR | PRRG4 | 3.12E-08 | 1.37E-02 | 0.47 |
| Gain | cg03842205 | 7 | 927934 |  | N_Shore | NM_015949 | Body | C7orf20 | 3.12E-08 | 1.37E-02 | 0.47 |
| Gain | cg22019158 | 1 | 27926683 | TRUE | N_Shelf | NM_001029882 | 5'UTR | AHDC1 | 3.16E-08 | 1.39E-02 | 0.44 |
| Gain | cg03777205 | 7 | 100182085 | TRUE | N_Shore | NM_002319 | Body | LRCH4 | 3.17E-08 | 1.39E-02 | 0.46 |
| Gain | cg20793071 | 1 | 54520450 |  | S_Shore | NM_153035;NM_004872 | Body;TSS1500 | C1orf83;TMEM59 | 3.18E-08 | 1.39E-02 | 0.48 |
| Gain | cg26669159 | 1 | 6967037 | TRUE |  | NM_015215 | Body | CAMTA1 | 3.25E-08 | 1.42E-02 | 0.48 |
| Gain | cg25406872 | 5 | 172385821 |  | Island | NR_026682;NR_026683;NM_016093 | Body;TSS200;TSS1500 | LOC100268168;LOC100268168;RPL26L1 | 3.25E-08 | 1.42E-02 | 0.46 |
| Gain | cg07838048 | 16 | 70720440 |  | Island | NM_138383 | TSS1500 | MTSS1L | 3.29E-08 | 1.44E-02 | 0.48 |
| Gain | cg01400401 | 19 | 4769531 |  |  | NR_029607;NR_027148 | TSS1500;Body | MIR7-3;C19orf30 | 3.30E-08 | 1.44E-02 | 0.48 |
| Gain | cg07194321 | 9 | 74524266 |  | N_Shore | NM_016014;NM_001025780 | 5'UTR;5'UTR | FAM108B1;FAM108B1 | 3.35E-08 | 1.47E-02 | 0.48 |
| Gain | cg14257676 | 8 | 10192642 |  | S_Shore | NM_001135671;NM_001135670;NM_012331 | Body;Body;Body | MSRA;MSRA;MSRA | 3.37E-08 | 1.48E-02 | 0.46 |
| Gain | cg06002197 | 16 | 81039458 |  | N_Shore | NM_001100624;NM_018455;NM_001100625;NM_020188 | TSS1500;TSS1500;TSS1500;5'UTR | CENPN;CENPN;CENPN;C16orf61 | 3.41E-08 | 1.49E-02 | 0.47 |
| Gain | cg22532475 | 10 | 104410764 | TRUE |  | NM_030912 | Body | TRIM8 | 3.41E-08 | 1.49E-02 | 0.47 |
| Gain | cg18792022 | 1 | 11709271 | TRUE | Island | NM_012168 | Body | FBXO2 | 3.42E-08 | 1.50E-02 | 0.47 |
| Gain | cg02243665 | 8 | 146013792 |  | S_Shore | NM_030580 | TSS1500 | ZNF34 | 3.42E-08 | 1.50E-02 | 0.46 |
| Gain | cg04174180 | 16 | 71496383 |  | S_Shore | NM_145911 | TSS1500 | ZNF23 | 3.44E-08 | 1.51E-02 | 0.47 |
| Gain | cg22635008 | 10 | 50970249 |  | Island | NM_001143996;NM_001143997;NM_018245 | 5'UTR;5'UTR;5'UTR | OGDHL;OGDHL;OGDHL | 3.46E-08 | 1.52E-02 | 0.47 |
| Gain | cg13112154 | 10 | 102996565 | TRUE | Island | NR_029380 | Body | FLJ41350 | 3.49E-08 | 1.53E-02 | 0.48 |
| Gain | cg08317252 | 19 | 1223163 |  | N_Shelf | NM_000455 | Body | STK11 | 3.50E-08 | 1.53E-02 | 0.46 |
| Gain | cg20234855 | 2 | 120980555 |  | Island | NR_000034 | Body | TMEM185B | 3.51E-08 | 1.54E-02 | 0.43 |
| Gain | cg01044580 | 14 | 21900820 |  |  | NM_001170629;NM_020920 | TSS1500;5'UTR | CHD8;CHD8 | 3.53E-08 | 1.55E-02 | 0.47 |
| Gain | cg10046620 | 6 | 27775042 |  | N_Shore | NM_003509 | TSS1500 | HIST1H2AI | 3.53E-08 | 1.55E-02 | 0.48 |
| Gain | cg00755063 | 19 | 13275288 | TRUE | S_Shore |  |  |  | 3.59E-08 | 1.57E-02 | 0.46 |
| Gain | cg01521106 | 17 | 78978395 |  | Island |  |  |  | 3.62E-08 | 1.58E-02 | 0.47 |
| Gain | cg26005232 | 1 | 27247829 |  | N_Shore | NM_006600 | TSS1500 | NUDC | 3.65E-08 | 1.60E-02 | 0.46 |
| Gain | cg23761616 | 4 | 159131339 |  | N_Shore | NM_018342 | TSS200 | TMEM144 | 3.71E-08 | 1.63E-02 | 0.47 |
| Gain | cg17412248 | 20 | 44803411 | TRUE | Island | NM_021248 | Body | CDH22 | 3.74E-08 | 1.64E-02 | 0.46 |
| Gain | cg09282289 | 13 | 20532174 |  | Island | NM_003453;NM_197968 | TSS1500;TSS1500 | ZMYM2;ZMYM2 | 3.78E-08 | 1.65E-02 | 0.47 |
| Gain | cg08584627 | 2 | 98703355 | TRUE | Island | NM_144992 | TSS1500 | VWA3B | 3.80E-08 | 1.66E-02 | 0.47 |
| Gain | cg00715047 | 17 | 73522054 | TRUE | Island | NM_001015002;NM_004524;NM_001031803 | 5'UTR;5'UTR;5'UTR | LLGL2;LLGL2;LLGL2 | 3.85E-08 | 1.69E-02 | 0.47 |
| Gain | cg15677364 | 6 | 30687221 |  | N_Shore | NM_178014 | TSS1500 | TUBB | 3.90E-08 | 1.71E-02 | 0.46 |
| Loss | cg01649611 | 2 | 43521066 | TRUE |  | NM_022065;NM_001083953 | Body;Body | THADA;THADA | 4.01E-08 | 1.76E-02 | -0.48 |
| Gain | cg11892747 | 3 | 160474372 |  | Island | NM_139245 | 1stExon | PPM1L | 4.03E-08 | 1.76E-02 | 0.48 |
| Gain | cg26002103 | 2 | 172967612 | TRUE | Island | NM_004405 | TSS200 | DLX2 | 4.03E-08 | 1.76E-02 | 0.47 |
| Gain | cg09848096 | 10 | 118928012 |  | Island |  |  |  | 4.04E-08 | 1.77E-02 | 0.47 |
| Gain | cg26366048 | 6 | 56820386 |  | S_Shore | NM_152731;NM_001144769 | 5'UTR;TSS1500 | BEND6;DST | 4.04E-08 | 1.77E-02 | 0.47 |
| Gain | cg07300558 | 18 | 35145353 |  | Island | NM_001025087;NM_001025089;NM_020180;NM_001025088 | 1stExon;1stExon;1stExon;1stExon | BRUNOL4;BRUNOL4;BRUNOL4;BRUNOL4 | 4.07E-08 | 1.78E-02 | 0.46 |
| Gain | cg00095976 | 6 | 118228060 |  | Island | NM_001029858 | TSS1500 | SLC35F1 | 4.07E-08 | 1.78E-02 | 0.47 |
| Gain | cg07068382 | 6 | 36947277 | TRUE |  | NM_014341 | Body | MTCH1 | 4.08E-08 | 1.79E-02 | 0.45 |
| Gain | cg14428310 | 11 | 87908722 |  | S_Shore | NM_022337 | TSS200 | RAB38 | 4.08E-08 | 1.79E-02 | 0.48 |
| Gain | cg26897150 | 17 | 57642763 |  | Island | NM_001166301;NM_024612 | TSS200;TSS200 | DHX40;DHX40 | 4.10E-08 | 1.79E-02 | 0.47 |
| Gain | cg04528819 | 7 | 130418315 |  | Island | NM_138693 | 1stExon | KLF14 | 4.11E-08 | 1.80E-02 | 0.47 |
| Gain | cg19574915 | 15 | 89195555 | TRUE |  | NM_002201 | Body | ISG20 | 4.13E-08 | 1.81E-02 | 0.46 |
| Gain | cg00015024 | 10 | 97803045 |  | Island | NM_001134376;NM_019084;NM_001134375 | TSS200;TSS200;TSS200 | CCNJ;CCNJ;CCNJ | 4.14E-08 | 1.81E-02 | 0.47 |
| Gain | cg05359249 | 2 | 220406470 |  | Island | NM_024536 | Body | CHPF | 4.20E-08 | 1.84E-02 | 0.47 |
| Gain | cg19641455 | 1 | 111506798 |  | Island | NM_018372;NM_001006945 | TSS1500;TSS1500 | C1orf103;C1orf103 | 4.20E-08 | 1.84E-02 | 0.47 |
| Loss | cg11594887 | 8 | 141535664 |  |  |  |  |  | 4.21E-08 | 1.84E-02 | -0.45 |
| Gain | cg24496614 | 11 | 61735970 |  | S_Shore | NM_002032 | TSS1500 | FTH1 | 4.23E-08 | 1.85E-02 | 0.46 |
| Gain | cg07584631 | 19 | 50366240 |  | Island | NM_007254 | Body | PNKP | 4.24E-08 | 1.86E-02 | 0.45 |
| Gain | cg18015985 | 5 | 176779051 |  | Island | NM_006816 | TSS200 | LMAN2 | 4.24E-08 | 1.86E-02 | 0.47 |
| Gain | cg13317687 | 9 | 108418916 |  | Island |  |  |  | 4.26E-08 | 1.86E-02 | 0.47 |
| Gain | cg07998461 | 10 | 35415601 |  | N_Shore | NM_182850;NM_182853;NM_183060;NM_001881;NM_183013;NM_181571 | TSS200;TSS200;TSS1500;TSS200;TSS200;TSS1500 | CREM;CREM;CREM;CREM;CREM;CREM | 4.27E-08 | 1.87E-02 | 0.46 |
| Gain | cg09137382 | 11 | 130731461 |  |  |  |  |  | 4.27E-08 | 1.87E-02 | 0.47 |
| Gain | cg17015340 | 13 | 113470495 |  | N_Shore | NM_015205;NM_032189 | Body;Body | ATP11A;ATP11A | 4.28E-08 | 1.87E-02 | 0.47 |
| Loss | cg07469815 | 2 | 174049232 | TRUE |  | NM_016653;NM_133646 | Body;Body | ZAK;ZAK | 4.34E-08 | 1.90E-02 | -0.48 |
| Gain | cg04640886 | 19 | 4769592 |  |  | NR_029607;NR_027148 | TSS1500;Body | MIR7-3;C19orf30 | 4.34E-08 | 1.90E-02 | 0.46 |
| Gain | cg03284308 | 16 | 81039325 |  | N_Shore | NM_001100624;NM_018455;NM_001100625;NM_020188 | TSS1500;TSS1500;TSS1500;5'UTR | CENPN;CENPN;CENPN;C16orf61 | 4.34E-08 | 1.90E-02 | 0.46 |
| Gain | cg02770054 | 19 | 36049123 | TRUE | Island | NM_000704 | Body | ATP4A | 4.36E-08 | 1.91E-02 | 0.47 |
| Gain | cg22335801 | 1 | 1149091 |  | Island | NM_003327 | Body | TNFRSF4 | 4.39E-08 | 1.92E-02 | 0.47 |
| Gain | cg25975256 | 17 | 43296395 |  | N_Shelf |  |  |  | 4.40E-08 | 1.93E-02 | 0.46 |
| Gain | cg24682012 | 11 | 6411087 |  | N_Shore | NR_027400;NM_000543;NM_001007593 | TSS1500;TSS1500;TSS1500 | SMPD1;SMPD1;SMPD1 | 4.40E-08 | 1.93E-02 | 0.48 |
| Gain | cg16057598 | 2 | 172967819 |  | Island | NM_004405 | TSS1500 | DLX2 | 4.48E-08 | 1.96E-02 | 0.47 |
| Gain | cg12194745 | 17 | 79423649 | TRUE | N_Shelf | NM_001080519 | Body | BAHCC1 | 4.49E-08 | 1.97E-02 | 0.48 |
| Gain | cg11322797 | 5 | 132155450 |  | Island |  |  |  | 4.50E-08 | 1.97E-02 | 0.47 |
| Gain | cg10686916 | 14 | 36004142 |  | Island | NM_032594 | 1stExon | INSM2 | 4.52E-08 | 1.98E-02 | 0.47 |
| Gain | cg11615607 | 3 | 120004483 |  | S_Shore |  |  |  | 4.56E-08 | 2.00E-02 | 0.47 |
| Gain | cg06382664 | 11 | 73098877 | TRUE |  | NM_152222;NM_032871 | 5'UTR;5'UTR | RELT;RELT | 4.56E-08 | 2.00E-02 | 0.45 |
| Loss | cg19955284 | 6 | 31833747 |  | S_Shelf | NM_025257 | Body | SLC44A4 | 4.60E-08 | 2.01E-02 | -0.47 |
| Gain | cg22030047 | 11 | 8954541 | TRUE |  | NM_020643;NM_020643 | 1stExon;5'UTR | C11orf16;C11orf16 | 4.62E-08 | 2.02E-02 | 0.46 |
| Gain | cg22118147 | 5 | 172144013 | TRUE |  |  |  |  | 4.62E-08 | 2.02E-02 | 0.47 |
| Gain | cg00474746 | 11 | 35684799 |  | Island | NM_017583 | 1stExon | TRIM44 | 4.64E-08 | 2.03E-02 | 0.46 |
| Gain | cg12850242 | 2 | 70484614 |  | N_Shore | NM_016297 | TSS1500 | PCYOX1 | 4.65E-08 | 2.04E-02 | 0.46 |
| Gain | cg18809289 | 10 | 45869816 | TRUE | Island | NM_000698 | 1stExon | ALOX5 | 4.67E-08 | 2.04E-02 | 0.47 |
| Gain | cg04290510 | 14 | 54864231 |  | Island | NM_005192;NM_001130851 | Body;Body | CDKN3;CDKN3 | 4.70E-08 | 2.06E-02 | 0.47 |
| Gain | cg26038465 | 22 | 38610514 |  | Island | NM_001161573;NM_001161574;NM_001161572;NM_012323 | Body;Body;Body;Body | MAFF;MAFF;MAFF;MAFF | 4.72E-08 | 2.06E-02 | 0.47 |
| Gain | cg26757711 | 19 | 6022589 | TRUE | N_Shelf | NM_000635;NM_134433 | Body;Body | RFX2;RFX2 | 4.73E-08 | 2.07E-02 | 0.47 |
| Gain | cg07785717 | 19 | 54485321 |  | Island | NM_031895;NR_030632 | Body;TSS1500 | CACNG8;MIR935 | 4.77E-08 | 2.09E-02 | 0.45 |
| Gain | cg05975727 | 2 | 149632640 |  | N_Shore | NM_004522 | TSS200 | KIF5C | 4.77E-08 | 2.09E-02 | 0.46 |
| Gain | cg22623927 | 10 | 134266933 | TRUE | Island |  |  |  | 4.77E-08 | 2.09E-02 | 0.47 |
| Loss | cg07866909 | 2 | 19849829 | TRUE |  |  |  |  | 4.78E-08 | 2.09E-02 | -0.47 |
| Gain | cg14665951 | 2 | 220492021 |  | N_Shore | NM_201574;NM_005070 | TSS1500;TSS1500 | SLC4A3;SLC4A3 | 4.80E-08 | 2.10E-02 | 0.46 |
| Gain | cg06666093 | 6 | 159291020 | TRUE | Island |  |  |  | 4.88E-08 | 2.14E-02 | 0.46 |
| Gain | cg19851222 | 22 | 38857936 | TRUE | S_Shore |  |  |  | 4.90E-08 | 2.15E-02 | 0.46 |
| Gain | cg26268968 | 12 | 53517561 | TRUE |  | NM_003578 | Body | SOAT2 | 4.92E-08 | 2.15E-02 | 0.47 |
| Gain | cg01798341 | 17 | 80842262 |  |  | NM_005993 | Body | TBCD | 4.93E-08 | 2.16E-02 | 0.47 |
| Gain | cg14058010 | 17 | 8029284 |  | N_Shore |  |  |  | 4.96E-08 | 2.17E-02 | 0.46 |
| Loss | cg08289567 | 4 | 129307231 | TRUE |  |  |  |  | 4.97E-08 | 2.18E-02 | -0.47 |
| Gain | cg08096786 | 16 | 57571099 |  | S_Shore | NM_033212 | TSS1500 | CCDC102A | 5.01E-08 | 2.19E-02 | 0.47 |
| Gain | cg07728507 | 16 | 88349782 |  |  |  |  |  | 5.01E-08 | 2.19E-02 | 0.47 |
| Gain | cg08057698 | 6 | 30568683 | TRUE |  | NM_002714 | 3'UTR | PPP1R10 | 5.09E-08 | 2.23E-02 | 0.47 |
| Gain | cg13941669 | 5 | 156886982 |  | Island | NM_001099287 | TSS200 | NIPAL4 | 5.11E-08 | 2.24E-02 | 0.45 |
| Gain | cg25820257 | 19 | 45996644 |  | N_Shore | NM_206900;NM_005619;NM_206901 | Body;Body;TSS200 | RTN2;RTN2;RTN2 | 5.13E-08 | 2.24E-02 | 0.46 |
| Gain | cg09754845 | 7 | 1408818 |  | Island |  |  |  | 5.16E-08 | 2.26E-02 | 0.47 |
| Gain | cg05617798 | 17 | 8113714 |  | Island | NM_004217 | 5'UTR | AURKB | 5.18E-08 | 2.27E-02 | 0.46 |
| Gain | cg12764034 | 1 | 75602846 |  | Island | NM_001001933 | Body | LHX8 | 5.20E-08 | 2.28E-02 | 0.47 |
| Gain | cg08955995 | 19 | 42503412 |  | Island | NM_002088 | Body | GRIK5 | 5.23E-08 | 2.29E-02 | 0.46 |
| Gain | cg00412842 | 20 | 17511479 |  | Island | NM_001161705;NM_001195 | Body;Body | BFSP1;BFSP1 | 5.23E-08 | 2.29E-02 | 0.47 |
| Gain | cg11599526 | 5 | 150449509 | TRUE |  | NM_006058 | 5'UTR | TNIP1 | 5.27E-08 | 2.31E-02 | 0.47 |
| Gain | cg16023943 | 10 | 27541331 |  | Island | NR_003525 | TSS200 | LOC387646 | 5.29E-08 | 2.32E-02 | 0.46 |
| Gain | cg22353329 | 17 | 77814357 |  | Island | NM_003655 | TSS1500 | CBX4 | 5.32E-08 | 2.33E-02 | 0.46 |
| Gain | cg03145963 | 11 | 104035151 |  | S_Shore | NM_025208;NM_033135 | TSS200;TSS200 | PDGFD;PDGFD | 5.34E-08 | 2.34E-02 | 0.47 |
| Gain | cg22476550 | 20 | 37590555 |  | N_Shore | NM_021931 | TSS1500 | DHX35 | 5.35E-08 | 2.34E-02 | 0.46 |
| Gain | cg19455840 | 10 | 102906171 | TRUE | Island |  |  |  | 5.35E-08 | 2.34E-02 | 0.47 |
| Gain | cg13140167 | 22 | 33454504 |  | Island | NM_001135774 | TSS200 | SYN3 | 5.35E-08 | 2.34E-02 | 0.46 |
| Gain | cg21144922 | 1 | 109204168 |  | Island | NM_144584;NM_001102592 | TSS200;TSS1500 | C1orf59;C1orf59 | 5.37E-08 | 2.35E-02 | 0.47 |
| Gain | cg18729787 | 6 | 33246307 |  | S_Shore | NM_003782 | 1stExon | B3GALT4 | 5.37E-08 | 2.35E-02 | 0.47 |
| Gain | cg27526665 | 3 | 24537050 |  | Island | NM_001128177;NM_001128176;NM_000461 | TSS1500;TSS1500;TSS1500 | THRB;THRB;THRB | 5.37E-08 | 2.35E-02 | 0.47 |
| Gain | cg06391982 | 3 | 13936753 | TRUE | Island |  |  |  | 5.41E-08 | 2.37E-02 | 0.46 |
| Gain | cg07860673 | 13 | 28494996 | TRUE | Island | NM_000209 | Body | PDX1 | 5.46E-08 | 2.39E-02 | 0.46 |
| Gain | cg10273210 | 3 | 160167665 |  | Island | NM_173084 | TSS200 | TRIM59 | 5.48E-08 | 2.40E-02 | 0.45 |
| Gain | cg00983637 | 1 | 26663115 | TRUE | Island | NM_001039775 | Body | AIM1L | 5.52E-08 | 2.42E-02 | 0.46 |
| Gain | cg13558810 | 1 | 36038708 |  | N_Shore | NM_178548 | TSS1500 | TFAP2E | 5.54E-08 | 2.42E-02 | 0.46 |
| Gain | cg27180315 | 7 | 75831194 |  | Island | NM_001110199 | TSS200 | SRRM3 | 5.59E-08 | 2.45E-02 | 0.47 |
| Gain | cg04388983 | 11 | 72929844 | TRUE | Island | NM_002564;NM_176071;NM_176072 | 5'UTR;5'UTR;5'UTR | P2RY2;P2RY2;P2RY2 | 5.61E-08 | 2.46E-02 | 0.47 |
| Gain | cg10106091 | 12 | 117176294 |  | Island | NM_024738;NM_032814;NM_001109903 | TSS1500;5'UTR;5'UTR | C12orf49;RNFT2;RNFT2 | 5.63E-08 | 2.46E-02 | 0.46 |
| Gain | cg10508778 | 12 | 110840579 |  | N_Shore | NM_001137664;NM_016238 | Body;Body | ANAPC7;ANAPC7 | 5.67E-08 | 2.48E-02 | 0.46 |
| Gain | cg27438889 | 10 | 123922704 | TRUE | N_Shore | NM_006997;NM_206862;NM_206860;NM_206861 | TSS1500;Body;TSS1500;Body | TACC2;TACC2;TACC2;TACC2 | 5.70E-08 | 2.49E-02 | 0.47 |
| Gain | cg12359904 | 3 | 156848454 |  |  |  |  |  | 5.70E-08 | 2.50E-02 | 0.45 |
| Gain | cg18867659 | 16 | 47178357 |  | Island | NM_018092 | TSS1500 | NETO2 | 5.71E-08 | 2.50E-02 | 0.47 |
| Gain | cg17100218 | 18 | 45275567 |  | Island |  |  |  | 5.79E-08 | 2.53E-02 | 0.46 |
| Loss | cg02821342 | 7 | 130793551 |  | N_Shore | NM_001145354;NR_015431 | TSS1500;Body | MKLN1;FLJ43663 | 5.80E-08 | 2.54E-02 | -0.45 |
| Loss | cg20194973 | 22 | 50524676 |  | N_Shelf | NM_139202;NM_015166 | TSS1500;TSS1500 | MLC1;MLC1 | 5.82E-08 | 2.55E-02 | -0.46 |
| Gain | cg06128028 | 7 | 35293892 |  | Island | NM_001166220;NM_001077653 | TSS200;TSS200 | TBX20;TBX20 | 5.84E-08 | 2.56E-02 | 0.47 |
| Gain | cg00228799 | 9 | 131580591 |  | Island | NM_004435 | TSS200 | ENDOG | 5.87E-08 | 2.57E-02 | 0.46 |
| Gain | cg21770622 | 11 | 46383066 |  |  | NM_201533;NM_001105540;NM_003646;NM_201532 | Body;TSS200;Body;Body | DGKZ;DGKZ;DGKZ;DGKZ | 5.93E-08 | 2.60E-02 | 0.46 |
| Gain | cg05806054 | 6 | 8103023 |  | S_Shore | NM_001135650;NM_004280 | TSS200;TSS200 | EEF1E1;EEF1E1 | 5.94E-08 | 2.60E-02 | 0.47 |
| Gain | cg04050000 | 2 | 31361687 |  | Island | NM_024572 | TSS200 | GALNT14 | 5.94E-08 | 2.60E-02 | 0.46 |
| Gain | cg19869746 | 6 | 7107108 |  | Island | NM_001003698;NM_001003700;NM_001168344;NM_001003699 | TSS1500;TSS1500;TSS1500;TSS1500 | RREB1;RREB1;RREB1;RREB1 | 5.98E-08 | 2.62E-02 | 0.45 |
| Gain | cg15463803 | 4 | 113436765 |  | Island | NM_024019 | 5'UTR | NEUROG2 | 6.01E-08 | 2.63E-02 | 0.46 |
| Gain | cg11998703 | 19 | 58446669 |  | Island | NM_133460;NM_133460 | 1stExon;5'UTR | ZNF418;ZNF418 | 6.02E-08 | 2.63E-02 | 0.47 |
| Gain | cg03588039 | 8 | 99182441 |  |  |  |  |  | 6.02E-08 | 2.63E-02 | 0.45 |
| Gain | cg20159687 | 2 | 231693070 |  | Island |  |  |  | 6.03E-08 | 2.64E-02 | 0.47 |
| Gain | cg10634619 | 1 | 3663435 |  | Island | NM_207306;NM_207306 | 1stExon;5'UTR | KIAA0495;KIAA0495 | 6.05E-08 | 2.65E-02 | 0.44 |
| Gain | cg25195288 | 11 | 115376182 |  | S_Shore | NM_001098517;NM_014333 | TSS1500;TSS1500 | CADM1;CADM1 | 6.08E-08 | 2.66E-02 | 0.46 |
| Gain | cg11086066 | 20 | 50159508 |  | Island | NM_012340;NM_001136021;NM_173091 | TSS1500;Body;TSS1500 | NFATC2;NFATC2;NFATC2 | 6.10E-08 | 2.67E-02 | 0.47 |
| Gain | cg00955482 | 18 | 712737 |  | Island | NM_017512;NM_001126123;NM_202758 | TSS200;TSS200;TSS1500 | ENOSF1;ENOSF1;ENOSF1 | 6.13E-08 | 2.68E-02 | 0.46 |
| Gain | cg09286183 | 16 | 2042466 |  | Island | NM_004209 | Body | SYNGR3 | 6.22E-08 | 2.72E-02 | 0.47 |
| Gain | cg08231709 | 8 | 99438968 | TRUE | Island | NM_020697 | TSS1500 | KCNS2 | 6.22E-08 | 2.72E-02 | 0.46 |
| Gain | cg20404336 | 12 | 133757909 |  | N_Shore | NM_001165886;NM_001165885;NM_001165887;NM_001165882;NM_001165883;NM_003415;NM_001165884 | TSS200;TSS200;TSS200;TSS200;TSS200;TSS200;TSS200;TSS200;TSS200 | ZNF268;ZNF268;ZNF268;ZNF268;ZNF268;ZNF268;ZNF268;ZNF268;ZNF268 | 6.27E-08 | 2.74E-02 | 0.45 |
| Gain | cg15386368 | 17 | 56833099 |  | Island | NM_014906 | TSS200 | PPM1E | 6.33E-08 | 2.77E-02 | 0.47 |
| Gain | cg02779535 | 20 | 34652280 | TRUE | N_Shore |  |  |  | 6.38E-08 | 2.79E-02 | 0.46 |
| Gain | cg05208605 | 11 | 116451437 | TRUE | Island |  |  |  | 6.41E-08 | 2.80E-02 | 0.47 |
| Gain | cg16704346 | 3 | 101280445 |  | N_Shore | NM_017819 | TSS1500 | RG9MTD1 | 6.41E-08 | 2.81E-02 | 0.47 |
| Loss | cg02636348 | 10 | 78923827 | TRUE |  | NM_001161353;NM_001161352;NM_002247;NM_001014797 | Body;Body;Body;Body | KCNMA1;KCNMA1;KCNMA1;KCNMA1 | 6.44E-08 | 2.82E-02 | -0.47 |
| Gain | cg00250500 | 10 | 77169599 |  | Island |  |  |  | 6.46E-08 | 2.83E-02 | 0.47 |
| Gain | cg02575697 | 1 | 39957307 |  | Island | NM_181809 | TSS200 | BMP8A | 6.54E-08 | 2.86E-02 | 0.46 |
| Gain | cg09004287 | 6 | 10426387 | TRUE | Island |  |  |  | 6.64E-08 | 2.91E-02 | 0.46 |
| Gain | cg16009734 | 1 | 65774867 |  | N_Shore | NM_014787 | Body | DNAJC6 | 6.65E-08 | 2.91E-02 | 0.47 |
| Gain | cg11719157 | 17 | 1466715 |  | S_Shore | NM_006224 | TSS1500 | PITPNA | 6.67E-08 | 2.92E-02 | 0.46 |
| Gain | cg21231458 | 20 | 21485933 |  | Island |  |  |  | 6.71E-08 | 2.94E-02 | 0.47 |
| Gain | cg25329013 | 11 | 22359326 |  | N_Shelf | NM_020346 | TSS1500 | SLC17A6 | 6.71E-08 | 2.94E-02 | 0.45 |
| Gain | cg17866732 | 2 | 110372875 |  | Island | NM_144710;NM_178584;NM_023016 | TSS1500;TSS1500;1stExon | SEPT10;SEPT10;ANKRD57 | 6.73E-08 | 2.95E-02 | 0.47 |
| Gain | cg08423149 | 7 | 5013451 |  | N_Shore | NR_023385;NR_023384;NR_015449 | TSS200;TSS200;TSS200 | RNF216L;RNF216L;RNF216L | 6.73E-08 | 2.95E-02 | 0.46 |
| Gain | cg13030331 | 2 | 241497599 |  | Island | NM_017844;NM_016552 | TSS200;TSS200 | ANKMY1;ANKMY1 | 6.79E-08 | 2.97E-02 | 0.42 |
| Gain | cg26718511 | 6 | 25652531 |  | Island | NM_006998;NM_006998 | 1stExon;5'UTR | SCGN;SCGN | 6.79E-08 | 2.97E-02 | 0.46 |
| Gain | cg19065831 | 4 | 48485289 |  | N_Shore | NM_152679 | TSS200 | SLC10A4 | 6.92E-08 | 3.03E-02 | 0.46 |
| Gain | cg07310916 | 2 | 232330273 |  | S_Shore | NM_005381 | TSS1500 | NCL | 6.94E-08 | 3.04E-02 | 0.47 |
| Gain | cg26078793 | 14 | 65006222 |  | N_Shore | NM_021979 | TSS1500 | HSPA2 | 6.97E-08 | 3.05E-02 | 0.46 |
| Gain | cg12258785 | 3 | 24537407 |  | Island | NM_001128177;NM_001128176;NM_000461 | TSS1500;TSS1500;TSS1500 | THRB;THRB;THRB | 6.99E-08 | 3.06E-02 | 0.47 |
| Gain | cg23448486 | 3 | 147129213 | TRUE | N_Shore | NM_003412 | Body | ZIC1 | 6.99E-08 | 3.06E-02 | 0.47 |
| Gain | cg15092561 | 21 | 45554033 |  | Island | NM_198155;NM_004649 | Body;Body | C21orf33;C21orf33 | 7.00E-08 | 3.06E-02 | 0.46 |
| Gain | cg23629722 | 17 | 16343399 |  | S_Shore | NR_027165;NR_027168;NR_027667;NR_027175;NR_027167;NR_027166;NR_027163;NR_027179;NR_0271 | Body;Body;Body;Body;Body;Body;Body;Body;Body;Body;Body;Body;Body;Body;TSS1500;Body;Body | NCRNA00188;NCRNA00188;NCRNA00188;NCRNA00188;NCRNA00188;NCRNA00188;NCRNA00188;NCRNA00188 | 7.03E-08 | 3.07E-02 | 0.46 |
| Gain | cg16007541 | 11 | 18728598 |  | S_Shore | NM_173588 | Body | IGSF22 | 7.03E-08 | 3.08E-02 | 0.47 |
| Gain | cg07924892 | 18 | 33767215 |  | Island | NM_017947 | TSS1500 | MOCOS | 7.05E-08 | 3.09E-02 | 0.45 |
| Gain | cg17328659 | 16 | 729498 |  | Island | NM_005861 | TSS1500 | STUB1 | 7.07E-08 | 3.09E-02 | 0.47 |
| Gain | cg23002268 | 5 | 172199318 |  | Island | NM_004417 | TSS1500 | DUSP1 | 7.09E-08 | 3.10E-02 | 0.45 |
| Gain | cg11286035 | 1 | 32714038 |  | Island | NM_032648 | Body | FAM167B | 7.10E-08 | 3.11E-02 | 0.46 |
| Gain | cg22278296 | 2 | 154335199 | TRUE | Island | NM_019845;NM_019845 | 1stExon;5'UTR | RPRM;RPRM | 7.13E-08 | 3.12E-02 | 0.46 |
| Gain | cg07314523 | 17 | 76183438 |  | Island | NM_001010982;NM_001145526;NM_001010982;NM_003258;NR_027083 | 1stExon;TSS200;5'UTR;TSS200;Body | AFMID;AFMID;AFMID;TK1;AFMID | 7.14E-08 | 3.12E-02 | 0.45 |
| Gain | cg21211187 | 13 | 114318347 |  |  |  |  |  | 7.24E-08 | 3.17E-02 | 0.46 |
| Gain | cg03062002 | 17 | 40832009 |  | Island | NM_016602 | Body | CCR10 | 7.29E-08 | 3.19E-02 | 0.46 |
| Gain | cg03734391 | 19 | 56182200 |  | N_Shelf | NM_001012478;NM_007279 | Body;Body | U2AF2;U2AF2 | 7.42E-08 | 3.25E-02 | 0.46 |
| Gain | cg01821557 | 3 | 49894282 | TRUE | S_Shore | NM_005879 | TSS1500 | TRAIP | 7.50E-08 | 3.28E-02 | 0.45 |
| Gain | cg14920289 | 14 | 23821149 |  | Island | NM_020372;NM_016609 | Body;Body | SLC22A17;SLC22A17 | 7.53E-08 | 3.30E-02 | 0.45 |
| Gain | cg12577850 | 6 | 139351113 |  | S_Shore | NM_021243 | 5'UTR | C6orf115 | 7.53E-08 | 3.30E-02 | 0.45 |
| Gain | cg15623062 | 6 | 31747133 |  |  | NM_006295 | Body | VARS | 7.60E-08 | 3.33E-02 | 0.46 |
| Gain | cg09181644 | 4 | 141490428 |  | S_Shore | NM_021833 | TSS1500 | UCP1 | 7.72E-08 | 3.38E-02 | 0.45 |
| Gain | cg09730123 | 16 | 1827948 |  | S_Shore | NM_080861 | Body | SPSB3 | 7.78E-08 | 3.40E-02 | 0.44 |
| Gain | cg09857513 | 7 | 120969044 |  | N_Shore | NM_057168;NM_016087 | TSS200;Body | WNT16;WNT16 | 7.78E-08 | 3.41E-02 | 0.47 |
| Gain | cg25507001 | 5 | 137475288 |  |  | NM_003551 | TSS200 | NME5 | 7.80E-08 | 3.41E-02 | 0.46 |
| Gain | cg19284211 | 20 | 20347495 |  | Island | NM_002196 | TSS1500 | INSM1 | 7.81E-08 | 3.42E-02 | 0.47 |
| Gain | cg08999807 | 13 | 92050776 |  | N_Shore | NM_004466 | TSS200 | GPC5 | 7.85E-08 | 3.44E-02 | 0.46 |
| Gain | cg15728256 | 1 | 40254184 |  | Island | NM_001720;NM_001720 | 5'UTR;1stExon | BMP8B;BMP8B | 7.91E-08 | 3.46E-02 | 0.46 |
| Gain | cg20852605 | 4 | 142054779 |  | S_Shore | NM_020724 | TSS200 | RNF150 | 7.93E-08 | 3.47E-02 | 0.46 |
| Gain | cg16226644 | 6 | 33246091 |  | S_Shore | NM_003782 | 1stExon | B3GALT4 | 7.99E-08 | 3.50E-02 | 0.47 |
| Gain | cg00401091 | 20 | 50159536 |  | S_Shore | NM_012340;NM_001136021;NM_173091 | TSS1500;Body;TSS1500 | NFATC2;NFATC2;NFATC2 | 8.04E-08 | 3.52E-02 | 0.46 |
| Gain | cg23235154 | 6 | 159290589 | TRUE | N_Shore |  |  |  | 8.16E-08 | 3.57E-02 | 0.46 |
| Gain | cg17080740 | 1 | 153601878 |  |  | NM_006271;NM_005979;NM_001024210 | 5'UTR;TSS1500;5'UTR | S100A1;S100A13;S100A13 | 8.22E-08 | 3.60E-02 | 0.46 |
| Gain | cg06869755 | 11 | 68199683 |  | N_Shore | NM_002335 | Body | LRP5 | 8.26E-08 | 3.62E-02 | 0.46 |
| Gain | cg18435449 | 19 | 58095445 |  | Island | NM_001010879 | TSS200 | ZIK1 | 8.29E-08 | 3.63E-02 | 0.46 |
| Gain | cg24300475 | 12 | 3601514 |  | Island | NM_019854 | Body | PRMT8 | 8.30E-08 | 3.63E-02 | 0.45 |
| Gain | cg06480265 | 6 | 24722060 |  | S_Shore |  |  |  | 8.31E-08 | 3.64E-02 | 0.45 |
| Gain | cg18352162 | 3 | 156848854 |  |  |  |  |  | 8.32E-08 | 3.64E-02 | 0.46 |
| Gain | cg26605700 | 16 | 2757124 |  | N_Shore | NM_018992 | Body | KCTD5 | 8.35E-08 | 3.65E-02 | 0.46 |
| Gain | cg17265115 | 4 | 734692 | TRUE | N_Shelf | NM_006315 | Body | PCGF3 | 8.39E-08 | 3.67E-02 | 0.46 |
| Gain | cg05752664 | 16 | 2136842 |  | N_Shelf | NM_001077183;NM_000548;NM_001114382 | Body;Body;Body | TSC2;TSC2;TSC2 | 8.41E-08 | 3.68E-02 | 0.46 |
| Gain | cg18486906 | 22 | 31218227 |  | Island | NM_030758 | Body | OSBP2 | 8.41E-08 | 3.68E-02 | 0.46 |
| Gain | cg12978575 | 19 | 58695028 |  | Island | NM_016325;NM_133502;NM_016324 | 5'UTR;5'UTR;5'UTR | ZNF274;ZNF274;ZNF274 | 8.41E-08 | 3.68E-02 | 0.46 |
| Gain | cg06810179 | 16 | 90038862 |  | Island | NM_145039;NR_003228;NR_003227;NM_145039;NR_003226 | 5'UTR;TSS200;TSS200;1stExon;TSS200 | CENPBD1;AFG3L1;AFG3L1;CENPBD1;AFG3L1 | 8.44E-08 | 3.69E-02 | 0.46 |
| Gain | cg16045612 | 5 | 158758490 |  | Island | NM_002187 | TSS1500 | IL12B | 8.54E-08 | 3.74E-02 | 0.47 |
| Gain | cg08885800 | 1 | 201084119 |  | Island |  |  |  | 8.56E-08 | 3.75E-02 | 0.46 |
| Gain | cg13458335 | 1 | 40254859 |  | Island | NM_001720 | TSS1500 | BMP8B | 8.58E-08 | 3.75E-02 | 0.45 |
| Gain | cg02304580 | 5 | 139124312 |  | N_Shore |  |  |  | 8.62E-08 | 3.77E-02 | 0.46 |
| Gain | cg20302082 | 2 | 120436534 |  | Island | NM_001105198;NM_030577;NM_001105199 | TSS1500;TSS1500;TSS1500 | TMEM177;TMEM177;TMEM177 | 8.62E-08 | 3.77E-02 | 0.46 |
| Gain | cg19095187 | 6 | 108437051 | TRUE | N_Shore |  |  |  | 8.64E-08 | 3.78E-02 | 0.46 |
| Gain | cg03565081 | 14 | 23478970 |  | N_Shore | NM_021944;NM_001130706;NM_001130708 | 5'UTR;5'UTR;5'UTR | C14orf93;C14orf93;C14orf93 | 8.67E-08 | 3.79E-02 | 0.46 |
| Gain | cg08996748 | 15 | 40651036 |  | Island | NM_033510 | Body | DISP2 | 8.69E-08 | 3.80E-02 | 0.45 |
| Gain | cg15458504 | 1 | 24828534 |  | N_Shore | NM_013441 | TSS1500 | RCAN3 | 8.72E-08 | 3.82E-02 | 0.47 |
| Gain | cg01186457 | 19 | 36336503 | TRUE | Island | NM_004646 | Body | NPHS1 | 8.74E-08 | 3.82E-02 | 0.46 |
| Gain | cg26833936 | 1 | 91303028 |  | S_Shore |  |  |  | 8.74E-08 | 3.82E-02 | 0.47 |
| Gain | cg26985149 | 17 | 40464935 |  | Island |  |  |  | 8.76E-08 | 3.83E-02 | 0.46 |
| Gain | cg11600807 | 11 | 17373180 |  | Island | NR_026750 | TSS200 | DKFZp686O24166 | 8.76E-08 | 3.83E-02 | 0.47 |
| Loss | cg07925311 | 16 | 57835436 |  | N_Shore | NM_005550;NM_001130100 | 5'UTR;5'UTR | KIFC3;KIFC3 | 8.82E-08 | 3.86E-02 | -0.46 |
| Gain | cg21328810 | 19 | 50829227 |  | Island | NM_004977 | Body | KCNC3 | 8.84E-08 | 3.87E-02 | 0.46 |
| Gain | cg12163823 | 2 | 242673721 |  | Island | NM_152783 | TSS1500 | D2HGDH | 8.87E-08 | 3.88E-02 | 0.46 |
| Gain | cg08983097 | 6 | 117586538 |  | Island | NM_153453;NM_182645 | TSS200;TSS200 | VGLL2;VGLL2 | 9.00E-08 | 3.94E-02 | 0.46 |
| Gain | cg02144516 | 1 | 1311909 |  | S_Shore | NM_001127229;NM_001127230;NM_017900 | TSS1500;TSS1500;TSS1500 | AURKAIP1;AURKAIP1;AURKAIP1 | 9.05E-08 | 3.96E-02 | 0.45 |
| Gain | cg02010772 | 16 | 58284274 |  | S_Shore | NM_001142302;NM_014157 | Body;Body | CCDC113;CCDC113 | 9.06E-08 | 3.96E-02 | 0.46 |
| Gain | cg13352836 | 16 | 31228263 |  | Island | NM_001008274;NM_152901 | Body;1stExon | TRIM72;PYDC1 | 9.06E-08 | 3.97E-02 | 0.46 |
| Gain | cg02250400 | 8 | 37698620 |  | N_Shore | NM_032777 | Body | GPR124 | 9.09E-08 | 3.98E-02 | 0.46 |
| Gain | cg23737062 | 15 | 63894296 |  | S_Shore | NM_203373 | 3'UTR | FBXL22 | 9.09E-08 | 3.98E-02 | 0.46 |
| Gain | cg23837438 | 10 | 135132457 |  | Island |  |  |  | 9.15E-08 | 4.00E-02 | 0.45 |
| Gain | cg08118159 | 19 | 41055208 |  | Island | NM_025213;NM_020971 | Body;Body | SPTBN4;SPTBN4 | 9.17E-08 | 4.01E-02 | 0.43 |
| Gain | cg05329888 | 22 | 50968312 |  | Island | NM_001113755;NM_001953;NM_001113756 | 5'UTR;5'UTR;TSS200 | TYMP;TYMP;TYMP | 9.28E-08 | 4.06E-02 | 0.46 |
| Gain | cg09829319 | 6 | 10882238 |  | N_Shore | NM_004752 | TSS200 | GCM2 | 9.34E-08 | 4.09E-02 | 0.46 |
| Gain | cg18485720 | 19 | 720740 |  | Island | NM_002579;NM_001040134 | Body;Body | PALM;PALM | 9.35E-08 | 4.09E-02 | 0.45 |
| Loss | cg07095346 | 3 | 52864812 |  |  | NM_001166449;NM_002218 | TSS200;TSS200 | ITIH4;ITIH4 | 9.41E-08 | 4.12E-02 | -0.46 |
| Gain | cg04387059 | 6 | 31698722 |  | S_Shore | NM_001288;NM_013974 | Body;TSS1500 | CLIC1;DDAH2 | 9.44E-08 | 4.13E-02 | 0.46 |
| Gain | cg12182408 | 14 | 104618441 |  | N_Shelf | NM_015656 | Body | KIF26A | 9.47E-08 | 4.14E-02 | 0.45 |
| Gain | cg09614389 | 20 | 25450839 |  |  | NM_025176 | Body | NINL | 9.48E-08 | 4.15E-02 | 0.46 |
| Gain | cg06628000 | 1 | 109756191 |  | N_Shore | NM_006513 | TSS1500 | SARS | 9.49E-08 | 4.15E-02 | 0.46 |
| Gain | cg06493386 | 8 | 72987797 | TRUE | Island | NM_007332;NM_007332 | 1stExon;5'UTR | TRPA1;TRPA1 | 9.77E-08 | 4.28E-02 | 0.46 |
| Gain | cg10025830 | 12 | 4136722 |  | N_Shelf |  |  |  | 9.78E-08 | 4.28E-02 | 0.46 |
| Loss | cg25225070 | 11 | 9587743 | TRUE |  |  |  |  | 9.87E-08 | 4.32E-02 | -0.46 |
| Gain | cg09727692 | 20 | 2505116 |  | N_Shore |  |  |  | 9.89E-08 | 4.33E-02 | 0.46 |
| Gain | cg24459409 | 19 | 46504412 | TRUE | S_Shore | NM_001080402 | Body | CCDC61 | 9.90E-08 | 4.33E-02 | 0.46 |
| Gain | cg05262711 | 16 | 2807476 |  | S_Shore | NM_016333 | Body | SRRM2 | 9.92E-08 | 4.34E-02 | 0.45 |
| Gain | cg12980128 | 18 | 55105322 |  | Island | NM_004852 | Body | ONECUT2 | 9.96E-08 | 4.36E-02 | 0.46 |
| Gain | cg15972949 | 7 | 44240380 |  | N_Shore | NM_006555 | TSS200 | YKT6 | 1.00E-07 | 4.38E-02 | 0.45 |
| Gain | cg12582003 | 9 | 131012968 |  | Island | NM_004408;NM_001005336 | Body;Body | DNM1;DNM1 | 1.00E-07 | 4.38E-02 | 0.46 |
| Gain | cg03192020 | 8 | 11204681 |  | N_Shore | NR_001578 | Body | TDH | 1.01E-07 | 4.40E-02 | 0.46 |
| Gain | cg23162310 | 5 | 172199255 |  | Island | NM_004417 | TSS1500 | DUSP1 | 1.01E-07 | 4.42E-02 | 0.46 |
| Gain | cg02268748 | 11 | 102139425 | TRUE | Island |  |  |  | 1.01E-07 | 4.43E-02 | 0.46 |
| Gain | cg11274962 | 19 | 7746796 |  | Island | NM_174894;NM_001042462;NM_001042461 | 5'UTR;5'UTR;5'UTR | TRAPPC5;TRAPPC5;TRAPPC5 | 1.02E-07 | 4.44E-02 | 0.46 |
| Gain | cg26776924 | 19 | 1969666 |  | Island | NM_001319 | 5'UTR | CSNK1G2 | 1.02E-07 | 4.45E-02 | 0.45 |
| Gain | cg19015951 | 10 | 98130398 |  |  | NM_012465 | Body | TLL2 | 1.02E-07 | 4.46E-02 | 0.45 |
| Loss | cg17185710 | 3 | 151984822 |  | N_Shore | NM_207292;NM_021038;NR_027038;NR_027037 | TSS1500;TSS1500;Body;Body | MBNL1;MBNL1;LOC401093;LOC401093 | 1.02E-07 | 4.47E-02 | -0.46 |
| Gain | cg06811183 | 3 | 48510438 |  |  | NM_016479 | 3'UTR | SHISA5 | 1.02E-07 | 4.48E-02 | 0.46 |
| Gain | cg26517584 | 19 | 1157683 |  | Island | NM_014963 | 5'UTR | SBNO2 | 1.02E-07 | 4.48E-02 | 0.45 |
| Gain | cg07529392 | 12 | 52215972 |  | Island | NM_001013690 | 1stExon | FIGNL2 | 1.02E-07 | 4.49E-02 | 0.45 |
| Gain | cg23049291 | 1 | 165204745 | TRUE | Island | NM_177398 | Body | LMX1A | 1.03E-07 | 4.50E-02 | 0.46 |
| Loss | cg16433737 | 12 | 6950123 |  |  | NM_002075 | 5'UTR | GNB3 | 1.03E-07 | 4.50E-02 | -0.46 |
| Gain | cg07523958 | 19 | 47181684 |  | Island | NM_001079880;NM_001079882;NM_016457;NM_001079881 | Body;Body;Body;Body | PRKD2;PRKD2;PRKD2;PRKD2 | 1.03E-07 | 4.50E-02 | 0.45 |
| Gain | cg04775668 | 2 | 127535185 |  | Island |  |  |  | 1.04E-07 | 4.57E-02 | 0.45 |
| Loss | cg01671895 | 11 | 100997799 |  | N_Shore | NM_000926 | Body | PGR | 1.04E-07 | 4.57E-02 | -0.46 |
| Gain | cg10855746 | 14 | 61938004 | TRUE |  | NM_006255 | Body | PRKCH | 1.05E-07 | 4.58E-02 | 0.45 |
| Gain | cg27535757 | 6 | 30434129 |  | Island |  |  |  | 1.05E-07 | 4.60E-02 | 0.46 |
| Gain | cg08367318 | 6 | 31743952 |  |  | NM_025258 | Body | C6orf27 | 1.05E-07 | 4.60E-02 | 0.46 |
| Gain | cg12165758 | 14 | 61789593 |  | S_Shore | NM_006255 | Body | PRKCH | 1.05E-07 | 4.61E-02 | 0.46 |
| Gain | cg00411411 | 10 | 103455165 |  | S_Shore | NM_022039 | TSS1500 | FBXW4 | 1.06E-07 | 4.62E-02 | 0.44 |
| Gain | cg01838971 | 11 | 45825448 |  | N_Shore | NM_001145266;NM_018389;NM_001145265 | TSS200;TSS1500;TSS200 | SLC35C1;SLC35C1;SLC35C1 | 1.07E-07 | 4.70E-02 | 0.45 |
| Gain | cg18812909 | 1 | 41328278 |  | Island | NM_133467 | TSS1500 | CITED4 | 1.08E-07 | 4.71E-02 | 0.46 |
| Gain | cg24151926 | 1 | 6240455 |  | Island | NM_015557 | TSS1500 | CHD5 | 1.08E-07 | 4.71E-02 | 0.45 |
| Gain | cg19162106 | 13 | 92050991 |  | N_Shore | NM_004466;NM_004466 | 5'UTR;1stExon | GPC5;GPC5 | 1.08E-07 | 4.72E-02 | 0.45 |
| Gain | cg18493027 | 14 | 70346151 |  | Island | NM_001034852;NM_022137;NM_022137;NM_001034852 | 5'UTR;1stExon;5'UTR;1stExon | SMOC1;SMOC1;SMOC1;SMOC1 | 1.08E-07 | 4.73E-02 | 0.46 |
| Loss | cg17971578 | 1 | 36852463 | TRUE | S_Shore | NM_032017 | TSS1500 | STK40 | 1.08E-07 | 4.75E-02 | -0.45 |
| Gain | cg12352399 | 3 | 25689774 | TRUE |  | NM_001068 | Body | TOP2B | 1.09E-07 | 4.76E-02 | 0.46 |
| Gain | cg09146183 | 22 | 38610376 |  | Island | NM_001161573;NM_001161572;NM_012323;NM_001161574 | Body;Body;Body;5'UTR | MAFF;MAFF;MAFF;MAFF | 1.09E-07 | 4.76E-02 | 0.45 |
| Gain | cg14836636 | 12 | 120106292 |  | S_Shore | NM_006253 | Body | PRKAB1 | 1.09E-07 | 4.77E-02 | 0.45 |
| Gain | cg16847696 | 7 | 124405760 |  | Island | NM_005302 | TSS200 | GPR37 | 1.09E-07 | 4.78E-02 | 0.45 |
| Gain | cg17213352 | 20 | 60886007 | TRUE | Island | NM_005560 | Body | LAMA5 | 1.10E-07 | 4.80E-02 | 0.46 |
| Gain | cg21093807 | 3 | 56717625 |  | Island | NM_001112736 | TSS1500 | C3orf63 | 1.10E-07 | 4.80E-02 | 0.46 |
| Gain | cg19780712 | 19 | 1116066 |  | N_Shore | NM_001100122;NM_014963 | Body;Body | SBNO2;SBNO2 | 1.10E-07 | 4.81E-02 | 0.44 |
| Loss | cg15110463 | 1 | 27113560 |  | N_Shore | NM_017837 | TSS1500 | PIGV | 1.10E-07 | 4.82E-02 | -0.46 |
| Gain | cg04367345 | 11 | 44332636 |  | Island | NM_021926 | TSS1500 | ALX4 | 1.11E-07 | 4.84E-02 | 0.46 |
| Gain | cg24960149 | 7 | 156786278 |  |  |  |  |  | 1.11E-07 | 4.86E-02 | 0.46 |
| Gain | cg00208967 | 19 | 10047924 |  | S_Shore | NM_058164 | TSS1500 | OLFM2 | 1.12E-07 | 4.88E-02 | 0.45 |
| Gain | cg14585700 | 9 | 37027605 | TRUE | Island | NM_016734 | Body | PAX5 | 1.12E-07 | 4.90E-02 | 0.45 |
| Gain | cg02538829 | 17 | 21226533 |  |  |  |  |  | 1.12E-07 | 4.91E-02 | 0.45 |
| Gain | cg21520772 | 12 | 58736428 | TRUE |  |  |  |  | 1.13E-07 | 4.93E-02 | 0.46 |
| Gain | cg01275297 | 20 | 35201324 |  | Island | NM_021809 | TSS1500 | TGIF2 | 1.13E-07 | 4.94E-02 | 0.44 |
| Gain | cg27447053 | 20 | 60795465 |  | Island | NM_007232 | TSS200 | HRH3 | 1.13E-07 | 4.96E-02 | 0.45 |
| Gain | cg16601415 | 19 | 10942243 |  |  | NM_001005361;NM_001005360;NM_004945;NM_001005362 | 3'UTR;3'UTR;3'UTR;3'UTR | DNM2;DNM2;DNM2;DNM2 | 1.14E-07 | 4.97E-02 | 0.45 |
| Gain | cg10636745 | 1 | 101775181 | TRUE |  |  |  |  | 1.14E-07 | 4.98E-02 | 0.45 |
