# Supplementary material for: Landscape of genome-wide age-related DNA methylation in breast tissue
Source: Oncotarget. 2017 Nov 29;8(70):114648–62. doi: 10.18632/oncotarget.22754 (PMC5777721; doi:10.18632/oncotarget.22754)
Supplement: Supplementary file 4 [file oncotarget-08-114648-s004.docx]

| **Probeset ID** | **Replicated in both adj. Normal and Tumor** | **Replicated in Adj. Normal** | **Replicated in Tumor** | **Differentially methylated according to ER status** | **Differentially methylated according to PR status** | **Differentially methylated according to HER2 status** |
| --- | --- | --- | --- | --- | --- | --- |
| cg26792755 | YES | YES | YES | NO | NO | NO |
| cg07243161 | YES | YES | YES | YES | NO | NO |
| cg11197101 | YES | YES | YES | YES | YES | NO |
| cg00702638 | YES | YES | YES | YES | NO | NO |
| cg07303143 | YES | YES | YES | YES | YES | NO |
| cg25743481 | YES | YES | YES | NO | NO | NO |
| cg12141030 | YES | YES | YES | NO | NO | NO |
| cg23606718 | YES | YES | YES | NO | NO | NO |
| cg06231995 | YES | YES | YES | NO | NO | NO |
| cg24888989 | YES | YES | YES | NO | NO | NO |
| cg06335143 | YES | YES | YES | YES | NO | NO |
| cg22736354 | YES | YES | YES | NO | NO | NO |
| cg12204897 | YES | YES | YES | YES | NO | NO |
| cg05917988 | YES | YES | YES | YES | YES | NO |
| cg13612447 | YES | YES | YES | NO | NO | NO |
| cg24079702 | YES | YES | YES | YES | YES | NO |
| cg06022942 | YES | YES | YES | NO | NO | NO |
| cg00590036 | YES | YES | YES | NO | NO | NO |
| cg01844642 | YES | YES | YES | YES | YES | NO |
| cg08318076 | YES | YES | YES | YES | NO | NO |
| cg03607117 | YES | YES | YES | NO | NO | NO |
| cg16867657 | YES | YES | YES | YES | NO | NO |
| cg23193759 | YES | YES | YES | NO | NO | NO |
| cg15906794 | YES | YES | YES | YES | NO | YES |
| cg21860429 | NO | NO | YES | YES | YES | YES |
| cg24466241 | YES | YES | YES | NO | NO | NO |
| cg07920503 | NO | NO | YES | YES | YES | NO |
| cg24724428 | YES | YES | YES | YES | YES | NO |
| cg11018337 | YES | YES | YES | NO | NO | NO |
| cg22454769 | YES | YES | YES | YES | YES | NO |
| cg06707236 | YES | YES | YES | YES | YES | NO |
| cg21159778 | YES | YES | YES | YES | YES | NO |
| cg17243289 | YES | YES | YES | YES | YES | NO |
| cg23027580 | YES | YES | YES | NO | NO | NO |
| cg19291355 | YES | YES | YES | YES | YES | NO |
| cg04100532 | YES | YES | YES | YES | YES | NO |
| cg12934382 | YES | YES | YES | YES | NO | NO |
| cg06458239 | YES | YES | YES | YES | YES | NO |
| cg14521746 | YES | YES | YES | YES | YES | NO |
| cg14704921 | YES | YES | YES | NO | NO | NO |
| cg01071511 | YES | YES | YES | YES | YES | NO |
| cg18468088 | YES | YES | YES | NO | NO | NO |
| cg00193021 | NO | NO | YES | YES | YES | YES |
| cg25287474 | YES | YES | YES | YES | YES | NO |
| cg10729426 | YES | YES | YES | YES | YES | NO |
| cg22016859 | YES | YES | YES | YES | YES | NO |
| cg25406872 | YES | YES | YES | NO | NO | NO |
| cg10943458 | YES | YES | YES | YES | YES | NO |
| cg04911280 | YES | YES | YES | YES | YES | NO |
| cg06580318 | YES | YES | YES | YES | YES | NO |
| cg02650266 | YES | YES | YES | YES | NO | NO |
| cg09022993 | NO | NO | YES | NO | NO | YES |
| cg12663656 | YES | YES | YES | NO | NO | NO |
| cg23433607 | YES | YES | YES | YES | NO | NO |
| cg11071401 | YES | YES | YES | NO | NO | NO |
| cg23995914 | YES | YES | YES | YES | YES | NO |
| cg01429039 | NO | NO | YES | NO | NO | YES |
| cg26147554 | YES | YES | YES | NO | NO | NO |
| cg00338080 | YES | YES | YES | YES | NO | NO |
| cg02721182 | YES | YES | YES | YES | YES | NO |
| cg21801378 | YES | YES | YES | NO | NO | NO |
| cg25826226 | YES | YES | YES | YES | YES | NO |
| cg18236477 | NO | NO | YES | YES | YES | YES |
| cg01644850 | YES | YES | YES | YES | YES | NO |
| cg03968755 | YES | YES | YES | YES | YES | YES |
| cg12016437 | YES | YES | YES | YES | YES | NO |
| cg15103195 | YES | YES | YES | YES | YES | NO |
| cg08095852 | YES | YES | YES | NO | NO | NO |
| cg17321954 | YES | YES | YES | YES | YES | NO |
| cg10947146 | YES | YES | YES | YES | NO | NO |
| cg09409930 | YES | YES | YES | YES | YES | NO |
| cg16026922 | YES | YES | YES | YES | YES | NO |
| cg00065935 | YES | YES | YES | YES | YES | NO |
| cg01019875 | YES | YES | YES | YES | YES | NO |
| cg08614301 | YES | YES | YES | YES | YES | NO |
| cg02383785 | YES | YES | YES | YES | YES | NO |
| cg23684204 | YES | YES | YES | YES | YES | NO |
| cg23193410 | YES | YES | YES | YES | YES | NO |
| cg00094518 | YES | YES | YES | YES | YES | NO |
| cg00292135 | YES | YES | YES | YES | YES | NO |
| cg19921279 | YES | YES | YES | YES | YES | NO |
| cg20893579 | YES | YES | YES | YES | YES | NO |
| cg14911690 | YES | YES | YES | YES | YES | NO |
| cg12373771 | YES | YES | YES | YES | YES | NO |
| cg06385324 | YES | YES | YES | YES | YES | NO |
| cg08097417 | YES | YES | YES | YES | YES | NO |
| cg13347071 | YES | YES | YES | YES | YES | NO |
| cg10820926 | YES | YES | YES | YES | YES | NO |
| cg06639320 | YES | YES | YES | YES | YES | NO |
| cg22719623 | YES | YES | YES | YES | YES | NO |
| cg11752769 | NO | NO | YES | YES | YES | NO |
| cg21186299 | YES | YES | YES | YES | NO | NO |
| cg00384539 | NO | NO | YES | NO | NO | YES |
| cg22958571 | YES | YES | YES | YES | YES | NO |
| cg04870212 | YES | YES | YES | NO | NO | NO |
| cg04268670 | YES | YES | YES | YES | YES | NO |
| cg26985149 | YES | YES | YES | YES | YES | NO |
| cg05906092 | YES | YES | YES | YES | NO | NO |
| cg14063191 | YES | YES | YES | YES | YES | NO |
| cg18148512 | YES | YES | YES | YES | NO | NO |
| cg03020208 | YES | YES | YES | YES | NO | NO |
| cg14956327 | NO | NO | YES | YES | YES | NO |
| cg02560808 | YES | YES | YES | YES | YES | NO |
| cg21105175 | YES | YES | YES | YES | YES | NO |
| cg27180315 | YES | YES | YES | NO | NO | NO |
| cg07392449 | YES | YES | YES | YES | NO | NO |
| cg07060551 | YES | YES | YES | YES | YES | NO |
| cg08541518 | YES | YES | YES | NO | NO | NO |
| cg26060489 | YES | YES | YES | YES | YES | NO |
| cg24903434 | YES | YES | YES | NO | NO | NO |
| cg07850604 | YES | YES | YES | YES | YES | NO |
| cg23091758 | YES | YES | YES | NO | NO | NO |
| cg21282131 | YES | YES | YES | NO | NO | NO |
| cg25204094 | YES | YES | YES | YES | YES | NO |
| cg21422770 | YES | YES | YES | NO | NO | NO |
| cg25478614 | YES | YES | YES | YES | YES | NO |
| cg08983097 | NO | NO | YES | YES | YES | YES |
| cg06493386 | NO | NO | YES | NO | NO | NO |
| cg23756251 | YES | YES | YES | YES | NO | YES |
| cg19639560 | YES | YES | YES | YES | YES | YES |
| cg07584631 | YES | YES | YES | YES | YES | NO |
| cg02118776 | YES | YES | YES | YES | YES | NO |
| cg04834794 | YES | YES | YES | YES | YES | NO |
| cg06144905 | YES | YES | YES | YES | YES | NO |
| cg07520506 | YES | YES | YES | YES | YES | NO |
| cg16847696 | YES | YES | YES | YES | NO | NO |
| cg08715791 | YES | YES | YES | YES | YES | NO |
| cg16541931 | YES | YES | YES | YES | YES | NO |
| cg12265959 | YES | YES | YES | YES | YES | NO |
| cg12685560 | YES | YES | YES | YES | YES | NO |
| cg20119148 | YES | YES | YES | YES | YES | NO |
| cg14633456 | YES | YES | YES | YES | YES | NO |
| cg23156348 | YES | YES | YES | YES | YES | NO |
| cg12451153 | YES | YES | YES | NO | NO | NO |
| cg03545227 | YES | YES | YES | YES | YES | NO |
| cg05617798 | YES | YES | YES | YES | YES | NO |
| cg07873590 | YES | YES | YES | YES | YES | NO |
| cg07180355 | YES | YES | YES | YES | YES | NO |
| cg00439658 | YES | YES | YES | YES | YES | NO |
| cg00059225 | YES | YES | YES | NO | NO | NO |
| cg25316339 | NO | NO | YES | YES | YES | NO |
| cg25942450 | YES | YES | YES | YES | YES | NO |
| cg15000966 | YES | YES | YES | YES | YES | NO |
| cg06073351 | NO | NO | YES | YES | YES | NO |
| cg12678562 | YES | YES | YES | YES | YES | NO |
| cg12584450 | YES | YES | YES | YES | YES | NO |
| cg26522319 | YES | YES | YES | YES | NO | NO |
| cg14361627 | YES | YES | YES | YES | YES | NO |
| cg16226644 | YES | YES | YES | YES | YES | NO |
| cg20426994 | YES | YES | YES | YES | YES | NO |
| cg18485720 | YES | YES | YES | YES | YES | NO |
| cg12603531 | YES | YES | YES | YES | YES | NO |
| cg27665659 | YES | YES | YES | YES | YES | NO |
| cg03555227 | YES | YES | YES | NO | NO | NO |
| cg05708550 | YES | YES | YES | YES | YES | NO |
| cg08317252 | YES | YES | YES | YES | YES | NO |
| cg27320127 | YES | YES | YES | YES | YES | NO |
| cg23158811 | YES | YES | YES | YES | YES | NO |
| cg09593028 | YES | YES | YES | YES | NO | NO |
| cg15158376 | YES | YES | YES | NO | NO | NO |
| cg21845273 | NO | NO | YES | YES | YES | YES |
| cg18633600 | YES | YES | YES | YES | YES | NO |
| cg07806886 | YES | YES | YES | YES | YES | NO |
| cg10625705 | YES | YES | YES | YES | NO | NO |
| cg22168941 | YES | YES | YES | YES | YES | NO |
| cg11082362 | YES | YES | YES | YES | YES | NO |
| cg02331561 | YES | YES | YES | YES | YES | NO |
| cg19996355 | YES | YES | YES | YES | YES | NO |
| cg02489908 | NO | NO | YES | YES | YES | NO |
| cg24393316 | NO | NO | YES | YES | YES | NO |
| cg18795809 | YES | YES | YES | YES | YES | NO |
| cg02243665 | YES | YES | YES | YES | YES | NO |
| cg16002963 | YES | YES | YES | YES | YES | NO |
| cg01912040 | YES | YES | YES | YES | YES | NO |
| cg23045594 | YES | YES | YES | YES | YES | NO |
| cg15091323 | NO | NO | YES | YES | YES | YES |
| cg02018902 | YES | YES | YES | NO | NO | NO |
| cg12669395 | YES | YES | YES | YES | YES | NO |
| cg00032205 | YES | YES | YES | YES | YES | NO |
| cg16480692 | YES | YES | YES | YES | YES | NO |
| cg04528819 | YES | YES | YES | YES | YES | NO |
| cg13016408 | NO | NO | YES | YES | YES | YES |
| cg02903907 | YES | YES | YES | YES | YES | NO |
| cg20308540 | YES | YES | YES | YES | YES | NO |
| cg13830081 | YES | YES | YES | NO | NO | NO |
| cg14311320 | YES | YES | YES | NO | NO | NO |
| cg10778288 | YES | YES | YES | YES | YES | NO |
| cg08120263 | YES | YES | YES | YES | YES | NO |
| cg03602730 | YES | YES | YES | YES | YES | NO |
| cg10686916 | YES | YES | YES | YES | YES | NO |
| cg19677989 | YES | YES | YES | NO | NO | NO |
| cg04880546 | YES | YES | YES | NO | NO | NO |
| cg03688818 | YES | YES | YES | YES | YES | NO |
| cg01627823 | YES | YES | YES | NO | NO | NO |
| cg24810473 | YES | YES | YES | YES | NO | NO |
| cg03036557 | YES | YES | YES | YES | YES | NO |
| cg12589298 | YES | YES | YES | NO | NO | NO |
| cg07927379 | NO | NO | YES | YES | NO | NO |
| cg25410668 | YES | YES | YES | YES | YES | NO |
| cg15936446 | YES | YES | YES | YES | YES | NO |
| cg26158959 | YES | YES | YES | YES | YES | YES |
| cg22635008 | YES | YES | YES | YES | YES | NO |
| cg11970192 | YES | YES | YES | NO | NO | NO |
| cg05396044 | YES | YES | YES | YES | NO | NO |
| cg16368750 | YES | YES | YES | YES | YES | NO |
| cg23813012 | YES | YES | YES | YES | YES | NO |
| cg07112260 | YES | YES | YES | NO | NO | NO |
| cg07687610 | YES | YES | YES | YES | YES | NO |
| cg09829319 | NO | NO | YES | NO | NO | YES |
| cg27518993 | YES | YES | YES | YES | YES | NO |
| cg13649056 | YES | YES | YES | YES | YES | NO |
| cg03943826 | YES | YES | YES | NO | NO | NO |
| cg19150852 | YES | YES | YES | NO | NO | NO |
| cg00246386 | YES | YES | YES | YES | YES | NO |
| cg02575697 | YES | YES | YES | YES | NO | NO |
| cg21431125 | YES | YES | YES | YES | YES | NO |
| cg16219246 | NO | NO | YES | YES | YES | YES |
| cg12559031 | YES | YES | YES | YES | YES | NO |
| cg01534416 | YES | YES | YES | YES | YES | NO |
| cg08888178 | YES | YES | YES | YES | YES | NO |
| cg09609212 | YES | YES | YES | NO | NO | NO |
| cg21927946 | YES | YES | YES | NO | NO | NO |
| cg15381304 | YES | YES | YES | NO | NO | NO |
| cg08999807 | YES | YES | YES | YES | YES | NO |
| cg08955995 | NO | NO | YES | YES | NO | YES |
| cg26776924 | YES | YES | YES | YES | YES | NO |
| cg04777071 | YES | YES | YES | NO | NO | NO |
| cg07720856 | YES | YES | YES | NO | NO | NO |
| cg25329013 | YES | YES | YES | NO | NO | NO |
| cg02189888 | YES | YES | YES | YES | YES | NO |
| cg02681442 | YES | YES | YES | YES | YES | NO |
| cg13317687 | NO | NO | YES | YES | NO | YES |
| cg24960149 | YES | YES | YES | YES | YES | YES |
| cg09226692 | YES | YES | YES | YES | NO | NO |
| cg00955482 | YES | YES | YES | NO | NO | NO |
| cg23854009 | YES | YES | YES | YES | YES | NO |
| cg12946225 | YES | YES | YES | YES | NO | NO |
| cg17652435 | NO | NO | YES | YES | YES | YES |
| cg18428193 | YES | YES | YES | YES | YES | NO |
| cg02355885 | YES | YES | YES | YES | YES | YES |
| cg23239612 | YES | YES | YES | YES | YES | NO |
| cg27541691 | YES | YES | YES | YES | YES | NO |
| cg20315346 | YES | YES | YES | YES | YES | NO |
| cg04269188 | NO | NO | YES | YES | YES | NO |
| cg08160331 | YES | YES | YES | YES | YES | NO |
| cg09012544 | YES | YES | YES | YES | YES | NO |
| cg01352586 | YES | YES | YES | NO | NO | NO |
| cg02851087 | YES | YES | YES | YES | NO | NO |
| cg05548912 | YES | YES | YES | NO | YES | NO |
| cg13854874 | YES | YES | YES | YES | YES | NO |
| cg01763090 | YES | YES | YES | YES | YES | NO |
| cg07193234 | NO | NO | YES | NO | NO | NO |
| cg09679923 | NO | NO | YES | YES | YES | YES |
| cg15425280 | YES | YES | YES | NO | NO | NO |
| cg24038454 | YES | YES | YES | YES | YES | NO |
| cg00095976 | NO | NO | YES | YES | YES | YES |
| cg02232208 | YES | YES | YES | YES | NO | YES |
| cg24422198 | YES | YES | YES | YES | YES | NO |
| cg12582003 | YES | YES | YES | YES | YES | NO |
| cg19891728 | NO | NO | YES | YES | YES | YES |
| cg02980971 | YES | YES | YES | YES | YES | NO |
| cg18546419 | YES | YES | YES | NO | NO | NO |
| cg24300475 | YES | YES | YES | NO | NO | NO |
| cg00088042 | YES | YES | YES | YES | YES | NO |
| cg15355387 | YES | YES | YES | NO | NO | NO |
| cg03710354 | YES | YES | YES | NO | NO | NO |
| cg18451114 | NO | NO | YES | YES | YES | NO |
| cg09857513 | YES | YES | YES | NO | NO | NO |
| cg26796095 | NO | NO | YES | YES | YES | NO |
| cg17213352 | YES | YES | YES | YES | YES | NO |
| cg01616356 | YES | YES | YES | YES | YES | NO |
| cg07224469 | YES | YES | YES | NO | NO | NO |
| cg11229185 | YES | YES | YES | YES | YES | NO |
| cg01356044 | YES | YES | YES | YES | YES | NO |
| cg21623865 | YES | YES | YES | YES | YES | NO |
| cg21724796 | YES | YES | YES | NO | NO | NO |
| cg26956371 | YES | YES | YES | YES | YES | NO |
| cg14415616 | NO | NO | YES | YES | YES | YES |
| cg23049291 | YES | YES | YES | YES | YES | NO |
| cg26734668 | YES | YES | YES | YES | YES | NO |
| cg22930390 | YES | YES | YES | NO | NO | NO |
| cg00221745 | YES | YES | YES | NO | NO | NO |
| cg11635304 | YES | YES | YES | NO | NO | NO |
| cg27522573 | NO | NO | YES | YES | YES | YES |
| cg19162106 | NO | NO | YES | YES | YES | NO |
| cg24150153 | YES | YES | YES | NO | YES | NO |
| cg09984392 | YES | YES | YES | YES | YES | NO |
| cg21709871 | YES | YES | YES | NO | NO | NO |
| cg07846081 | YES | YES | YES | YES | YES | NO |
| cg09941452 | YES | YES | YES | YES | NO | NO |
| cg02662658 | YES | YES | YES | NO | NO | NO |
| cg03041738 | NO | NO | YES | NO | NO | YES |
| cg17674725 | YES | YES | YES | YES | YES | NO |
| cg14848772 | YES | YES | YES | YES | NO | NO |
| cg24847541 | NO | NO | YES | YES | YES | NO |
| cg13941669 | YES | YES | YES | YES | NO | YES |
| cg14319409 | YES | YES | YES | NO | NO | NO |
| cg23483765 | YES | YES | YES | NO | NO | YES |
| cg14261272 | NO | NO | YES | YES | YES | YES |
| cg13140167 | YES | YES | YES | YES | YES | YES |
| cg12584718 | YES | YES | YES | NO | NO | NO |
| cg07066898 | YES | YES | YES | YES | YES | NO |
| cg26974111 | YES | YES | YES | YES | YES | NO |
| cg26118408 | YES | YES | YES | NO | NO | NO |
| cg08209133 | YES | YES | YES | YES | YES | NO |
| cg08057698 | YES | YES | YES | YES | YES | NO |
| cg01875838 | YES | YES | YES | YES | YES | NO |
| cg21567504 | YES | YES | YES | YES | YES | YES |
| cg05176991 | YES | YES | YES | YES | YES | NO |
| cg21449170 | YES | YES | YES | YES | YES | NO |
| cg15611624 | YES | YES | YES | YES | NO | YES |
| cg03578662 | YES | YES | YES | NO | YES | NO |
| cg15778457 | NO | NO | YES | NO | NO | NO |
| cg03801300 | YES | YES | YES | NO | NO | NO |
| cg24051481 | NO | NO | YES | YES | YES | YES |
| cg12764034 | NO | NO | YES | YES | NO | YES |
| cg03734391 | YES | YES | YES | YES | YES | NO |
| cg23998119 | YES | YES | YES | NO | NO | NO |
| cg09499629 | YES | YES | YES | YES | YES | NO |
| cg03350900 | YES | YES | YES | YES | NO | NO |
| cg04387059 | YES | YES | YES | YES | YES | NO |
| cg10548038 | YES | YES | YES | YES | YES | NO |
| cg03777205 | YES | YES | YES | NO | NO | NO |
| cg23654174 | YES | YES | YES | YES | YES | NO |
| cg06879152 | NO | NO | YES | YES | YES | YES |
| cg06128028 | YES | YES | YES | YES | NO | NO |
| cg18729787 | YES | YES | YES | YES | YES | NO |
| cg00350942 | YES | YES | YES | YES | YES | NO |
| cg13029847 | YES | YES | YES | YES | NO | YES |
| cg20302082 | YES | YES | YES | NO | NO | NO |
| cg10732215 | NO | NO | YES | YES | YES | NO |
| cg22278296 | YES | YES | YES | YES | YES | NO |
| cg04367345 | NO | NO | YES | YES | YES | YES |
| cg07589899 | YES | YES | YES | YES | YES | NO |
| cg13805608 | YES | YES | YES | YES | YES | NO |
| cg01637011 | YES | YES | YES | YES | YES | NO |
| cg20777920 | YES | YES | YES | NO | NO | NO |
| cg18569335 | YES | YES | YES | YES | YES | NO |
| cg07388347 | YES | YES | YES | NO | NO | NO |
| cg05262711 | YES | YES | YES | YES | YES | NO |
| cg18622870 | YES | YES | YES | NO | NO | NO |
| cg23681664 | NO | NO | YES | YES | YES | YES |
| cg04169021 | YES | YES | YES | NO | NO | NO |
| cg08243849 | YES | YES | YES | YES | YES | NO |
| cg27209578 | YES | YES | YES | NO | NO | NO |
| cg25778262 | YES | YES | YES | YES | YES | NO |
| cg07420163 | YES | YES | YES | NO | NO | NO |
| cg03554283 | YES | YES | YES | YES | NO | NO |
| cg14674720 | YES | YES | YES | YES | YES | YES |
| cg09853371 | NO | NO | YES | YES | YES | YES |
| cg10432620 | YES | YES | YES | NO | NO | NO |
| cg19851222 | YES | YES | YES | YES | YES | NO |
| cg16896647 | YES | YES | YES | NO | NO | NO |
| cg16704346 | YES | YES | YES | YES | YES | NO |
| cg20879085 | YES | YES | YES | YES | YES | NO |
| cg07584066 | YES | YES | YES | YES | YES | NO |
| cg18867659 | YES | YES | YES | NO | NO | NO |
| cg16969368 | YES | YES | YES | YES | YES | NO |
| cg05965414 | YES | YES | YES | YES | YES | NO |
| cg26005082 | YES | YES | YES | NO | NO | NO |
| cg24229963 | YES | YES | YES | YES | NO | NO |
| cg15149655 | YES | YES | YES | YES | NO | NO |
| cg14371731 | YES | YES | YES | NO | YES | NO |
| cg24871691 | YES | YES | YES | NO | NO | YES |
| cg15789607 | NO | YES | NO | N/A | N/A | N/A |
| cg20977794 | NO | NO | YES | YES | YES | YES |
| cg25108022 | YES | YES | YES | NO | NO | NO |
| cg22642485 | NO | NO | YES | YES | YES | NO |
| cg02732509 | YES | YES | YES | YES | NO | NO |
| cg23861715 | YES | YES | YES | YES | NO | NO |
| cg03015610 | YES | YES | YES | YES | YES | NO |
| cg17497271 | YES | YES | YES | NO | NO | NO |
| cg25427880 | YES | YES | YES | NO | NO | NO |
| cg14023774 | NO | NO | YES | YES | YES | NO |
| cg04368796 | YES | YES | YES | YES | YES | NO |
| cg13030331 | YES | YES | YES | NO | NO | NO |
| cg03719693 | NO | NO | YES | YES | YES | YES |
| cg17110586 | YES | YES | YES | YES | NO | NO |
| cg24214260 | YES | YES | YES | YES | YES | NO |
| cg17694795 | NO | NO | YES | YES | YES | YES |
| cg01717446 | NO | NO | YES | YES | YES | NO |
| cg19065831 | NO | NO | YES | YES | YES | YES |
| cg07860673 | NO | NO | YES | YES | YES | NO |
| cg14920289 | YES | YES | YES | YES | YES | NO |
| cg03399905 | YES | YES | YES | YES | NO | NO |
| cg09730123 | YES | YES | YES | YES | YES | NO |
| cg14112635 | YES | YES | YES | YES | NO | NO |
| cg06034933 | NO | NO | YES | YES | YES | NO |
| cg26921969 | YES | YES | YES | YES | YES | NO |
| cg19584530 | NO | NO | YES | YES | YES | NO |
| cg01122755 | YES | YES | YES | YES | YES | NO |
| cg25047092 | YES | YES | YES | YES | YES | NO |
| cg03539717 | YES | YES | YES | YES | YES | NO |
| cg00870662 | YES | YES | YES | NO | NO | NO |
| cg22235877 | YES | YES | YES | YES | NO | NO |
| cg08885800 | NO | NO | YES | YES | YES | YES |
| cg02636348 | YES | YES | YES | YES | YES | NO |
| cg06869755 | NO | NO | YES | YES | YES | NO |
| cg05632420 | YES | YES | YES | YES | YES | NO |
| cg03397716 | YES | YES | YES | NO | NO | NO |
| cg25622481 | YES | YES | YES | YES | YES | NO |
| cg26518431 | YES | YES | YES | YES | YES | NO |
| cg00840310 | YES | YES | YES | YES | YES | YES |
| cg04775668 | YES | YES | YES | YES | NO | NO |
| cg04290510 | YES | YES | YES | NO | NO | NO |
| cg04875128 | YES | YES | YES | YES | YES | NO |
| cg24637426 | YES | YES | YES | YES | YES | NO |
| cg03065467 | NO | NO | YES | YES | YES | NO |
| cg05540369 | YES | YES | YES | YES | YES | NO |
| cg21725716 | YES | YES | YES | NO | NO | NO |
| cg07523958 | YES | YES | YES | YES | YES | NO |
| cg09849846 | YES | YES | YES | NO | NO | NO |
| cg00593900 | YES | YES | YES | NO | NO | NO |
| cg23500537 | YES | YES | YES | YES | YES | NO |
| cg01541867 | NO | NO | YES | YES | YES | YES |
| cg01214340 | YES | YES | YES | YES | YES | NO |
| cg04684267 | YES | YES | YES | YES | YES | NO |
| cg08584627 | YES | YES | YES | NO | NO | NO |
| cg19586576 | YES | YES | YES | YES | YES | NO |
| cg18145080 | YES | YES | YES | NO | NO | NO |
| cg14844236 | YES | YES | YES | YES | YES | YES |
| cg00745389 | NO | NO | YES | YES | YES | YES |
| cg00460776 | YES | YES | YES | NO | NO | NO |
| cg08484671 | YES | YES | YES | YES | YES | NO |
| cg18928900 | YES | YES | YES | YES | YES | NO |
| cg01143804 | YES | YES | YES | YES | NO | NO |
| cg19106932 | YES | YES | YES | YES | YES | NO |
| cg21475150 | YES | YES | YES | YES | YES | NO |
| cg25090514 | YES | YES | YES | YES | NO | NO |
| cg22624255 | YES | YES | YES | NO | NO | NO |
| cg06718763 | YES | YES | YES | YES | YES | YES |
| cg15201877 | YES | YES | YES | YES | YES | NO |
| cg19284211 | NO | NO | YES | YES | YES | YES |
| cg04769392 | YES | YES | YES | YES | NO | NO |
| cg26129417 | YES | YES | YES | YES | YES | NO |
| cg16601415 | YES | YES | YES | YES | NO | NO |
| cg24497836 | YES | YES | YES | YES | YES | NO |
| cg10102418 | YES | YES | YES | YES | NO | NO |
| cg08687825 | NO | NO | YES | YES | YES | YES |
| cg27645517 | YES | YES | YES | YES | YES | NO |
| cg13201172 | YES | YES | YES | YES | YES | NO |
| cg26417361 | YES | YES | YES | NO | NO | NO |
| cg19421368 | YES | YES | YES | YES | YES | NO |
| cg00252781 | YES | YES | YES | YES | YES | NO |
| cg04293888 | YES | YES | YES | YES | NO | NO |
| cg27559724 | YES | YES | YES | YES | YES | NO |
| cg17100218 | YES | YES | YES | NO | NO | NO |
| cg07502389 | YES | YES | YES | YES | NO | YES |
| cg11051055 | NO | NO | YES | NO | NO | NO |
| cg06493994 | YES | YES | YES | NO | NO | YES |
| cg22775000 | NO | NO | YES | YES | YES | NO |
| cg04420878 | YES | YES | YES | YES | YES | NO |
| cg14484688 | YES | YES | YES | YES | YES | NO |
| cg22149516 | YES | YES | YES | NO | NO | NO |
| cg19743881 | YES | YES | YES | YES | YES | NO |
| cg00664406 | YES | YES | YES | YES | NO | NO |
| cg23428445 | YES | YES | YES | YES | NO | NO |
| cg08694014 | YES | YES | YES | YES | YES | NO |
| cg10806820 | YES | YES | YES | YES | YES | NO |
| cg23652182 | YES | YES | YES | YES | YES | NO |
| cg00830285 | NO | NO | YES | YES | YES | NO |
| cg00983637 | YES | YES | YES | NO | NO | NO |
| cg15703035 | YES | YES | YES | YES | YES | NO |
| cg13202816 | YES | YES | YES | YES | YES | NO |
| cg16778903 | YES | YES | YES | YES | NO | NO |
| cg26290632 | YES | YES | YES | YES | YES | NO |
| cg16113692 | YES | YES | YES | NO | NO | NO |
| cg23582919 | NO | YES | NO | N/A | N/A | N/A |
| cg07584077 | YES | YES | YES | NO | NO | NO |
| cg20413454 | YES | YES | YES | YES | YES | NO |
| cg02722188 | NO | NO | YES | YES | YES | NO |
| cg02770054 | NO | NO | YES | YES | YES | NO |
| cg18982073 | YES | YES | YES | YES | YES | NO |
| cg16367511 | YES | YES | YES | YES | YES | NO |
| cg25090510 | YES | YES | YES | YES | NO | YES |
| cg04241863 | YES | YES | YES | YES | NO | NO |
| cg16370875 | YES | YES | YES | YES | YES | NO |
| cg02805871 | NO | NO | YES | YES | YES | NO |
| cg24159214 | YES | YES | YES | NO | NO | NO |
| cg05757654 | YES | YES | YES | YES | YES | NO |
| cg06666093 | YES | YES | YES | NO | NO | NO |
| cg25148589 | YES | YES | YES | NO | NO | YES |
| cg25505610 | YES | YES | YES | YES | YES | NO |
| cg01693650 | NO | YES | NO | N/A | N/A | N/A |
| cg16717122 | YES | YES | YES | YES | YES | YES |
| cg11359984 | YES | YES | YES | YES | YES | NO |
| cg08734918 | NO | NO | YES | YES | NO | NO |
| cg18493027 | NO | NO | YES | YES | YES | NO |
| cg25390506 | NO | YES | NO | N/A | N/A | N/A |
| cg26377000 | NO | NO | YES | YES | YES | NO |
| cg13564889 | YES | YES | YES | NO | NO | NO |
| cg21477033 | YES | YES | YES | YES | YES | NO |
| cg08748615 | YES | YES | YES | YES | YES | NO |
| cg22310062 | YES | YES | YES | YES | YES | NO |
| cg05725804 | NO | NO | YES | YES | YES | NO |
| cg05492839 | YES | YES | YES | YES | YES | NO |
| cg05602648 | YES | YES | YES | YES | YES | NO |
| cg17437852 | YES | YES | YES | YES | NO | NO |
| cg18267374 | YES | YES | YES | YES | YES | YES |
| cg14594876 | YES | YES | YES | YES | YES | NO |
| cg07225598 | NO | NO | YES | YES | YES | NO |
| cg20638016 | YES | YES | YES | NO | NO | NO |
| cg09286183 | YES | YES | YES | NO | NO | NO |
| cg14585700 | NO | NO | YES | YES | YES | NO |
| cg25712567 | NO | NO | YES | YES | YES | NO |
| cg01667837 | YES | YES | YES | NO | NO | NO |
| cg25987194 | NO | NO | YES | YES | YES | YES |
| cg10825040 | YES | YES | YES | NO | NO | NO |
| cg12850242 | YES | YES | YES | YES | YES | NO |
| cg21723861 | NO | NO | YES | YES | YES | NO |
| cg04309212 | YES | YES | YES | YES | YES | NO |
| cg27509306 | YES | YES | YES | YES | YES | NO |
| cg26897150 | YES | YES | YES | YES | YES | NO |
| cg00743094 | YES | YES | YES | YES | YES | YES |
| cg08482682 | YES | YES | YES | YES | YES | NO |
| cg04453050 | YES | YES | YES | YES | NO | NO |
| cg00303541 | YES | YES | YES | NO | NO | NO |
| cg16379337 | YES | YES | YES | YES | NO | NO |
| cg12401425 | YES | YES | YES | NO | NO | NO |
| cg11667101 | YES | YES | YES | YES | NO | NO |
| cg04653012 | YES | YES | YES | YES | YES | NO |
| cg23105471 | YES | YES | YES | YES | YES | NO |
| cg19410770 | NO | YES | NO | N/A | N/A | N/A |
| cg22335801 | NO | NO | YES | YES | NO | NO |
| cg23553442 | YES | YES | YES | YES | YES | NO |
| cg02442436 | YES | YES | YES | YES | YES | NO |
| cg15201635 | YES | YES | YES | NO | NO | NO |
| cg18417423 | YES | YES | YES | YES | YES | NO |
| cg07189157 | YES | YES | YES | NO | NO | NO |
| cg14107807 | YES | YES | YES | YES | YES | NO |
| cg12226046 | YES | YES | YES | YES | YES | NO |
| cg17412248 | YES | YES | YES | YES | YES | NO |
| cg09453076 | YES | YES | YES | YES | YES | NO |
| cg10284662 | YES | YES | YES | YES | NO | NO |
| cg10548492 | YES | YES | YES | YES | YES | NO |
| cg02479575 | NO | YES | NO | N/A | N/A | N/A |
| cg07570470 | YES | YES | YES | YES | YES | NO |
| cg00878605 | YES | YES | YES | YES | YES | NO |
| cg27438889 | YES | YES | YES | YES | YES | NO |
| cg14940405 | YES | YES | YES | YES | YES | NO |
| cg23002268 | YES | YES | YES | NO | NO | NO |
| cg14901671 | YES | YES | YES | YES | YES | NO |
| cg05208605 | NO | NO | YES | YES | YES | YES |
| cg07547549 | YES | YES | YES | YES | YES | YES |
| cg09379755 | YES | YES | YES | YES | YES | NO |
| cg04084157 | YES | YES | YES | YES | YES | NO |
| cg26517584 | YES | YES | YES | YES | YES | NO |
| cg02847037 | YES | YES | YES | YES | YES | NO |
| cg14424579 | YES | YES | YES | YES | YES | NO |
| cg09754845 | YES | YES | YES | YES | YES | NO |
| cg00237391 | YES | YES | YES | NO | NO | NO |
| cg08578136 | NO | NO | YES | YES | YES | YES |
| cg23490822 | YES | YES | YES | YES | YES | NO |
| cg09202227 | YES | YES | YES | NO | NO | NO |
| cg24151926 | YES | YES | YES | NO | NO | NO |
| cg15822346 | YES | YES | YES | YES | YES | NO |
| cg21182694 | YES | YES | YES | YES | YES | NO |
| cg23448486 | NO | NO | YES | YES | YES | YES |
| cg19581424 | YES | YES | YES | YES | YES | YES |
| cg10254690 | YES | YES | YES | YES | YES | NO |
| cg09190280 | YES | YES | YES | YES | YES | NO |
| cg00516222 | NO | NO | YES | YES | YES | YES |
| cg22186515 | NO | NO | YES | YES | YES | NO |
| cg14686745 | NO | YES | NO | N/A | N/A | N/A |
| cg16489193 | NO | YES | NO | N/A | N/A | N/A |
| cg14692377 | NO | YES | NO | N/A | N/A | N/A |
| cg00208967 | NO | NO | YES | YES | YES | YES |
| cg20345589 | YES | YES | YES | NO | NO | NO |
| cg21877855 | YES | YES | YES | YES | YES | NO |
| cg17758721 | YES | YES | YES | NO | YES | NO |
| cg17724147 | NO | NO | YES | YES | YES | NO |
| cg25061843 | YES | YES | YES | YES | YES | YES |
| cg23956238 | NO | YES | NO | N/A | N/A | N/A |
| cg13112154 | NO | NO | YES | NO | NO | YES |
| cg01592801 | YES | YES | YES | YES | YES | NO |
| cg04782470 | YES | YES | YES | YES | YES | NO |
| cg13503413 | YES | YES | YES | YES | YES | YES |
| cg07169873 | YES | YES | YES | YES | YES | YES |
| cg11908384 | YES | YES | YES | NO | NO | NO |
| cg15623062 | YES | YES | YES | YES | YES | YES |
| cg04768203 | NO | YES | NO | N/A | N/A | N/A |
| cg00068155 | YES | YES | YES | YES | YES | NO |
| cg01521106 | NO | NO | YES | YES | YES | YES |
| cg10129041 | YES | YES | YES | YES | YES | NO |
| cg03192020 | NO | NO | YES | YES | NO | NO |
| cg05308656 | YES | YES | YES | YES | YES | NO |
| cg02821342 | YES | YES | YES | YES | YES | NO |
| cg09977376 | NO | YES | NO | N/A | N/A | N/A |
| cg24496614 | YES | YES | YES | YES | YES | NO |
| cg15677364 | YES | YES | YES | YES | YES | NO |
| cg15995695 | YES | YES | YES | YES | YES | NO |
| cg11274962 | YES | YES | YES | YES | YES | YES |
| cg08289567 | YES | YES | YES | YES | YES | NO |
| cg21231458 | NO | NO | YES | NO | NO | NO |
| cg16430166 | YES | YES | YES | YES | YES | NO |
| cg24459409 | YES | YES | YES | NO | NO | NO |
| cg22353329 | YES | YES | YES | YES | YES | NO |
| cg01186457 | YES | YES | YES | NO | YES | NO |
| cg09195550 | YES | YES | YES | YES | YES | NO |
| cg02949991 | NO | YES | NO | N/A | N/A | N/A |
| cg01867395 | YES | YES | YES | YES | YES | YES |
| cg10696191 | YES | YES | YES | YES | YES | NO |
| cg25380622 | NO | YES | NO | N/A | N/A | N/A |
| cg12639558 | YES | YES | YES | YES | YES | NO |
| cg02447229 | YES | YES | YES | YES | NO | NO |
| cg06737494 | YES | YES | YES | NO | NO | NO |
| cg19897172 | NO | YES | NO | N/A | N/A | N/A |
| cg07640648 | NO | NO | YES | YES | YES | NO |
| cg02048412 | YES | YES | YES | NO | NO | NO |
| cg08622677 | YES | YES | YES | YES | YES | NO |
| cg23517605 | YES | YES | YES | NO | NO | NO |
| cg01410314 | YES | YES | YES | YES | YES | NO |
| cg09364688 | YES | YES | YES | YES | NO | YES |
| cg23197992 | NO | YES | NO | N/A | N/A | N/A |
| cg04577625 | YES | YES | YES | YES | YES | NO |
| cg16792632 | YES | YES | YES | YES | YES | NO |
| cg19841423 | YES | YES | YES | YES | YES | NO |
| cg26400491 | YES | YES | YES | YES | YES | NO |
| cg24550865 | YES | YES | YES | YES | YES | NO |
| cg04079139 | NO | YES | NO | N/A | N/A | N/A |
| cg26545313 | YES | YES | YES | NO | YES | NO |
| cg10523019 | YES | YES | YES | NO | NO | NO |
| cg14676592 | NO | YES | NO | N/A | N/A | N/A |
| cg11299854 | NO | YES | NO | N/A | N/A | N/A |
| cg09997676 | YES | YES | YES | YES | YES | NO |
| cg05342634 | YES | YES | YES | YES | YES | NO |
| cg06648759 | NO | YES | NO | N/A | N/A | N/A |
| cg02538829 | YES | YES | YES | YES | YES | NO |
| cg17265115 | YES | YES | YES | YES | YES | YES |
| cg20852605 | NO | YES | NO | N/A | N/A | N/A |
| cg00481951 | YES | YES | YES | YES | YES | NO |
| cg01798341 | YES | YES | YES | YES | YES | NO |
| cg19780712 | YES | YES | YES | YES | YES | NO |
| cg11614451 | NO | YES | NO | N/A | N/A | N/A |
| cg05173913 | YES | YES | YES | YES | YES | NO |
| cg08541521 | YES | YES | YES | YES | YES | NO |
| cg21899500 | YES | YES | YES | YES | NO | NO |
| cg19343530 | YES | YES | YES | YES | YES | NO |
| cg23761616 | NO | YES | NO | N/A | N/A | N/A |
| cg26490949 | YES | YES | YES | YES | YES | NO |
| cg07838048 | YES | YES | YES | YES | YES | NO |
| cg02945056 | YES | YES | YES | YES | YES | NO |
| cg05936895 | YES | YES | YES | NO | NO | NO |
| cg08153345 | YES | YES | YES | NO | NO | NO |
| cg03667051 | NO | YES | NO | N/A | N/A | N/A |
| cg06002197 | YES | YES | YES | YES | YES | NO |
| cg11896587 | YES | YES | YES | YES | YES | NO |
| cg17576288 | NO | NO | YES | YES | YES | YES |
| cg19281363 | NO | NO | YES | YES | YES | NO |
| cg26804595 | YES | YES | YES | YES | YES | NO |
| cg19464016 | NO | NO | YES | YES | YES | NO |
| cg05975727 | YES | YES | YES | NO | NO | NO |
| cg22598841 | NO | YES | NO | N/A | N/A | N/A |
| cg04684152 | YES | YES | YES | YES | YES | NO |
| cg00228799 | NO | YES | NO | N/A | N/A | N/A |
| cg25112291 | YES | YES | YES | YES | YES | NO |
| cg04271792 | NO | YES | NO | N/A | N/A | N/A |
| cg03777575 | YES | YES | YES | YES | YES | NO |
| cg05555455 | YES | YES | YES | YES | YES | NO |
| cg02381192 | YES | YES | YES | NO | NO | NO |
| cg22623927 | YES | YES | YES | YES | YES | NO |
| cg01908954 | YES | YES | YES | YES | YES | YES |
| cg00015024 | NO | NO | YES | YES | YES | NO |
| cg14279035 | YES | YES | YES | YES | YES | NO |
| cg13744663 | NO | NO | YES | YES | YES | NO |
| cg16045612 | NO | NO | YES | YES | YES | NO |
| cg00180930 | YES | YES | YES | YES | YES | NO |
| cg21453443 | NO | NO | YES | YES | YES | NO |
| cg17421143 | YES | YES | YES | YES | YES | NO |
| cg06810179 | YES | YES | YES | NO | NO | NO |
| cg09136245 | NO | YES | NO | N/A | N/A | N/A |
| cg07798694 | NO | YES | NO | N/A | N/A | N/A |
| cg06365567 | YES | YES | YES | YES | NO | YES |
| cg08243728 | YES | YES | YES | YES | YES | NO |
| cg10193817 | NO | YES | NO | N/A | N/A | N/A |
| cg15728256 | NO | NO | YES | YES | YES | NO |
| cg16909962 | NO | YES | NO | N/A | N/A | N/A |
| cg05590196 | NO | YES | NO | N/A | N/A | N/A |
| cg22532475 | YES | YES | YES | YES | YES | NO |
| cg05630556 | NO | YES | NO | N/A | N/A | N/A |
| cg24544803 | YES | YES | YES | YES | YES | NO |
| cg00708380 | YES | YES | YES | YES | NO | NO |
| cg09177567 | YES | YES | YES | YES | YES | NO |
| cg25139493 | YES | YES | YES | YES | YES | NO |
| cg02865822 | NO | YES | NO | N/A | N/A | N/A |
| cg07121644 | YES | YES | YES | NO | NO | YES |
| cg25225070 | YES | YES | YES | YES | YES | YES |
| cg07068382 | NO | NO | YES | YES | YES | NO |
| cg24315421 | YES | YES | YES | YES | YES | NO |
| cg24016624 | NO | YES | NO | N/A | N/A | N/A |
| cg07314523 | NO | YES | NO | N/A | N/A | N/A |
| cg18549036 | NO | YES | NO | N/A | N/A | N/A |
| cg19455840 | NO | NO | YES | NO | NO | YES |
| cg01561864 | YES | YES | YES | YES | YES | NO |
| cg26002103 | NO | YES | NO | N/A | N/A | N/A |
| cg08286012 | NO | NO | YES | YES | YES | NO |
| cg14836636 | NO | YES | NO | N/A | N/A | N/A |
| cg08785215 | NO | YES | NO | N/A | N/A | N/A |
| cg20159687 | YES | YES | YES | YES | YES | YES |
| cg21483700 | YES | YES | YES | YES | YES | NO |
| cg00474746 | NO | YES | NO | N/A | N/A | N/A |
| cg00573165 | NO | NO | YES | YES | YES | NO |
| cg06900404 | YES | YES | YES | YES | YES | YES |
| cg10593047 | YES | YES | YES | YES | YES | NO |
| cg26038465 | YES | YES | YES | NO | NO | NO |
| cg08904363 | NO | YES | NO | N/A | N/A | N/A |
| cg13948585 | YES | YES | YES | NO | NO | NO |
| cg24408436 | YES | YES | YES | YES | NO | YES |
| cg06889481 | YES | YES | YES | YES | YES | NO |
| cg27526665 | NO | YES | NO | N/A | N/A | N/A |
| cg08461586 | YES | YES | YES | YES | YES | NO |
| cg20404336 | NO | YES | NO | N/A | N/A | N/A |
| cg06993413 | NO | YES | NO | N/A | N/A | N/A |
| cg09229918 | NO | NO | YES | YES | YES | NO |
| cg19451698 | YES | YES | YES | NO | NO | NO |
| cg19055803 | NO | YES | NO | N/A | N/A | N/A |
| cg24682012 | NO | YES | NO | N/A | N/A | N/A |
| cg09369954 | NO | YES | NO | N/A | N/A | N/A |
| cg05261559 | YES | YES | YES | YES | NO | NO |
| cg08169949 | YES | YES | YES | YES | YES | NO |
| cg07908508 | NO | YES | NO | N/A | N/A | N/A |
| cg13220457 | NO | YES | NO | N/A | N/A | N/A |
| cg16032102 | NO | YES | NO | N/A | N/A | N/A |
| cg09378441 | NO | YES | NO | N/A | N/A | N/A |
| cg04640886 | NO | YES | NO | N/A | N/A | N/A |
| cg11615607 | YES | YES | YES | YES | YES | NO |
| cg03490115 | NO | YES | NO | N/A | N/A | N/A |
| cg04008429 | NO | YES | NO | N/A | N/A | N/A |
| cg16321523 | YES | YES | YES | NO | NO | NO |
| cg18143296 | NO | NO | YES | NO | YES | NO |
| cg02748419 | NO | NO | YES | YES | YES | NO |
| cg05412990 | NO | YES | NO | N/A | N/A | N/A |
| cg09058748 | YES | YES | YES | YES | YES | NO |
| cg07751287 | YES | YES | YES | YES | YES | YES |
| cg19869746 | NO | NO | YES | NO | YES | NO |
| cg02933139 | NO | YES | NO | N/A | N/A | N/A |
| cg04050000 | NO | YES | NO | N/A | N/A | N/A |
| cg20797766 | YES | YES | YES | YES | YES | NO |
| cg13352836 | NO | NO | YES | YES | NO | NO |
| cg16181396 | YES | YES | YES | YES | YES | NO |
| cg03314644 | NO | YES | NO | N/A | N/A | N/A |
| cg01687680 | NO | NO | YES | YES | YES | YES |
| cg24621354 | YES | YES | YES | YES | YES | NO |
| cg24853724 | NO | YES | NO | N/A | N/A | N/A |
| cg06811183 | YES | YES | YES | NO | NO | NO |
| cg23981354 | NO | YES | NO | N/A | N/A | N/A |
| cg00601711 | NO | YES | NO | N/A | N/A | N/A |
| cg04986675 | NO | YES | NO | N/A | N/A | N/A |
| cg22367678 | NO | YES | NO | N/A | N/A | N/A |
| cg11915218 | NO | NO | YES | YES | YES | NO |
| cg10469100 | NO | YES | NO | N/A | N/A | N/A |
| cg18055623 | YES | YES | YES | YES | NO | NO |
| cg16192371 | NO | YES | NO | N/A | N/A | N/A |
| cg03678609 | YES | YES | YES | YES | YES | NO |
| cg06391982 | YES | YES | YES | NO | NO | NO |
| cg06603828 | NO | YES | NO | N/A | N/A | N/A |
| cg05329888 | NO | YES | NO | N/A | N/A | N/A |
| cg10665379 | NO | YES | NO | N/A | N/A | N/A |
| cg00312553 | NO | NO | YES | YES | NO | YES |
| cg07172885 | NO | YES | NO | N/A | N/A | N/A |
| cg26366048 | YES | YES | YES | YES | YES | NO |
| cg07529392 | YES | YES | YES | YES | YES | NO |
| cg08231709 | YES | YES | YES | YES | YES | NO |
| cg01799653 | NO | NO | YES | YES | YES | YES |
| cg20793071 | YES | YES | YES | YES | YES | YES |
| cg08342886 | NO | YES | NO | N/A | N/A | N/A |
| cg16465768 | NO | YES | NO | N/A | N/A | N/A |
| cg14305262 | NO | YES | NO | N/A | N/A | N/A |
| cg25334393 | NO | YES | NO | N/A | N/A | N/A |
| cg21328810 | NO | YES | NO | N/A | N/A | N/A |
| cg19401340 | YES | YES | YES | YES | YES | NO |
| cg08220519 | NO | YES | NO | N/A | N/A | N/A |
| cg25585523 | NO | YES | NO | N/A | N/A | N/A |
| cg12182408 | NO | YES | NO | N/A | N/A | N/A |
| cg26546105 | YES | YES | YES | YES | YES | NO |
| cg25670583 | YES | YES | YES | YES | YES | NO |
| cg24948962 | YES | YES | YES | YES | YES | NO |
| cg12983839 | NO | YES | NO | N/A | N/A | N/A |
| cg11599526 | NO | YES | NO | N/A | N/A | N/A |
| cg09278980 | NO | NO | YES | YES | NO | YES |
| cg09513380 | NO | NO | YES | YES | YES | NO |
| cg13814485 | NO | YES | NO | N/A | N/A | N/A |
| cg08571883 | NO | YES | NO | N/A | N/A | N/A |
| cg09298289 | YES | YES | YES | YES | YES | NO |
| cg05668372 | YES | YES | YES | YES | YES | YES |
| cg22118147 | NO | YES | NO | N/A | N/A | N/A |
| cg16510278 | NO | YES | NO | N/A | N/A | N/A |
| cg09105193 | NO | NO | YES | YES | YES | NO |
| cg13442820 | NO | YES | NO | N/A | N/A | N/A |
| cg16019898 | YES | YES | YES | YES | YES | NO |
| cg07785717 | YES | YES | YES | NO | NO | NO |
| cg08147050 | NO | YES | NO | N/A | N/A | N/A |
| cg01789150 | YES | YES | YES | YES | YES | NO |
| cg21770622 | NO | YES | NO | N/A | N/A | N/A |
| cg07848706 | NO | YES | NO | N/A | N/A | N/A |
| cg08224212 | NO | YES | NO | N/A | N/A | N/A |
| cg06544316 | NO | YES | NO | N/A | N/A | N/A |
| cg08431693 | YES | YES | YES | NO | NO | NO |
| cg03251287 | NO | NO | YES | YES | YES | NO |
| cg07447260 | NO | YES | NO | N/A | N/A | N/A |
| cg04749646 | NO | NO | YES | YES | YES | YES |
| cg08377768 | NO | YES | NO | N/A | N/A | N/A |
| cg19031565 | NO | YES | NO | N/A | N/A | N/A |
| cg13575161 | NO | NO | YES | YES | YES | NO |
| cg21956434 | NO | YES | NO | N/A | N/A | N/A |
| cg13682722 | NO | YES | NO | N/A | N/A | N/A |
| cg08278892 | NO | YES | NO | N/A | N/A | N/A |
| cg26816688 | NO | YES | NO | N/A | N/A | N/A |
| cg08770761 | NO | YES | NO | N/A | N/A | N/A |
| cg06382664 | NO | NO | YES | YES | YES | NO |
| cg21555177 | NO | YES | NO | N/A | N/A | N/A |
| cg07122805 | NO | NO | YES | YES | YES | NO |
| cg23040782 | NO | YES | NO | N/A | N/A | N/A |
| cg07924892 | NO | YES | NO | N/A | N/A | N/A |
| cg02220965 | YES | YES | YES | YES | YES | NO |
| cg12018403 | NO | YES | NO | N/A | N/A | N/A |
| cg18435449 | NO | NO | YES | NO | NO | YES |
| cg04878973 | NO | YES | NO | N/A | N/A | N/A |
| cg16010628 | NO | YES | NO | N/A | N/A | N/A |
| cg04925385 | NO | YES | NO | N/A | N/A | N/A |
| cg26596350 | NO | YES | NO | N/A | N/A | N/A |
| cg25590826 | NO | YES | NO | N/A | N/A | N/A |
| cg09784307 | YES | YES | YES | YES | YES | NO |
| cg15458504 | NO | YES | NO | N/A | N/A | N/A |
| cg26367730 | NO | YES | NO | N/A | N/A | N/A |
| cg26830108 | NO | YES | NO | N/A | N/A | N/A |
| cg04845466 | NO | YES | NO | N/A | N/A | N/A |
| cg16023943 | NO | YES | NO | N/A | N/A | N/A |
| cg17009433 | NO | YES | NO | N/A | N/A | N/A |
| cg01929377 | NO | YES | NO | N/A | N/A | N/A |
| cg09630437 | NO | YES | NO | N/A | N/A | N/A |
| cg08078694 | NO | YES | NO | N/A | N/A | N/A |
| cg04174180 | NO | NO | YES | YES | YES | NO |
| cg11286035 | NO | YES | NO | N/A | N/A | N/A |
| cg15777261 | NO | YES | NO | N/A | N/A | N/A |
| cg26189067 | NO | NO | YES | YES | YES | NO |
| cg11204099 | NO | YES | NO | N/A | N/A | N/A |
| cg01400401 | NO | YES | NO | N/A | N/A | N/A |
| cg15571730 | NO | NO | YES | NO | NO | NO |
| cg09614389 | NO | YES | NO | N/A | N/A | N/A |
| cg17080740 | NO | YES | NO | N/A | N/A | N/A |
| cg17152981 | NO | YES | NO | N/A | N/A | N/A |
| cg10894072 | NO | YES | NO | N/A | N/A | N/A |
| cg14064148 | NO | YES | NO | N/A | N/A | N/A |
| cg02268748 | NO | YES | NO | N/A | N/A | N/A |
| cg26112929 | NO | YES | NO | N/A | N/A | N/A |
| cg15697646 | NO | YES | NO | N/A | N/A | N/A |
| cg26355004 | NO | YES | NO | N/A | N/A | N/A |
| cg22866825 | NO | YES | NO | N/A | N/A | N/A |
| cg20121753 | NO | YES | NO | N/A | N/A | N/A |
| cg08118159 | NO | NO | YES | YES | YES | NO |
| cg25105633 | NO | YES | NO | N/A | N/A | N/A |
| cg24480120 | YES | YES | YES | YES | YES | NO |
| cg12641275 | NO | YES | NO | N/A | N/A | N/A |
| cg02144516 | NO | NO | YES | YES | YES | NO |
| cg02759846 | NO | YES | NO | N/A | N/A | N/A |
| cg01329687 | NO | YES | NO | N/A | N/A | N/A |
| cg05218976 | NO | YES | NO | N/A | N/A | N/A |
| cg19688118 | NO | YES | NO | N/A | N/A | N/A |
| cg08207660 | NO | YES | NO | N/A | N/A | N/A |
| cg12189835 | NO | YES | NO | N/A | N/A | N/A |
| cg10795738 | NO | YES | NO | N/A | N/A | N/A |
| cg06142537 | YES | YES | YES | YES | YES | NO |
| cg18213931 | NO | YES | NO | N/A | N/A | N/A |
| cg10634619 | NO | NO | YES | NO | NO | YES |
| cg15972949 | NO | NO | NO | N/A | N/A | N/A |
| cg00489219 | NO | YES | NO | N/A | N/A | N/A |
| cg10106091 | NO | YES | NO | N/A | N/A | N/A |
| cg20673481 | NO | NO | NO | N/A | N/A | N/A |
| cg01585703 | NO | YES | NO | N/A | N/A | N/A |
| cg26272477 | NO | YES | NO | N/A | N/A | N/A |
| cg19095187 | NO | NO | YES | YES | NO | NO |
| cg11086066 | NO | YES | NO | N/A | N/A | N/A |
| cg09861346 | NO | YES | NO | N/A | N/A | N/A |
| cg27106513 | NO | NO | NO | N/A | N/A | N/A |
| cg03062002 | NO | NO | NO | N/A | N/A | N/A |
| cg06061878 | NO | YES | NO | N/A | N/A | N/A |
| cg22224597 | NO | YES | NO | N/A | N/A | N/A |
| cg07667161 | NO | YES | NO | N/A | N/A | N/A |
| cg15061025 | NO | YES | NO | N/A | N/A | N/A |
| cg25397922 | NO | YES | NO | N/A | N/A | N/A |
| cg05290394 | NO | YES | NO | N/A | N/A | N/A |
| cg09146183 | NO | YES | NO | N/A | N/A | N/A |
| cg17328659 | NO | YES | NO | N/A | N/A | N/A |
| cg10999598 | NO | YES | NO | N/A | N/A | N/A |
| cg25100532 | NO | YES | NO | N/A | N/A | N/A |
| cg00092518 | NO | YES | NO | N/A | N/A | N/A |
| cg23737062 | NO | YES | NO | N/A | N/A | N/A |
| cg08541297 | NO | YES | NO | N/A | N/A | N/A |
| cg06741803 | NO | YES | NO | N/A | N/A | N/A |
| cg03565081 | NO | YES | NO | N/A | N/A | N/A |
| cg23244398 | NO | YES | NO | N/A | N/A | N/A |
| cg00888561 | NO | YES | NO | N/A | N/A | N/A |
| cg16603784 | NO | YES | NO | N/A | N/A | N/A |
| cg22030047 | NO | YES | NO | N/A | N/A | N/A |
| cg27535757 | NO | YES | NO | N/A | N/A | N/A |
| cg01144053 | NO | NO | NO | N/A | N/A | N/A |
| cg07671586 | NO | YES | NO | N/A | N/A | N/A |
| cg09848096 | NO | YES | NO | N/A | N/A | N/A |
| cg02071825 | NO | YES | NO | N/A | N/A | N/A |
| cg11866674 | NO | NO | YES | YES | YES | YES |
| cg14241323 | NO | YES | NO | N/A | N/A | N/A |
| cg08013270 | NO | YES | NO | N/A | N/A | N/A |
| cg26475688 | NO | YES | NO | N/A | N/A | N/A |
| cg07095346 | NO | NO | YES | YES | YES | NO |
| cg08096786 | NO | YES | NO | N/A | N/A | N/A |
| cg14058010 | NO | YES | NO | N/A | N/A | N/A |
| cg02078370 | NO | YES | NO | N/A | N/A | N/A |
| cg15993083 | NO | NO | NO | N/A | N/A | N/A |
| cg12563644 | NO | YES | NO | N/A | N/A | N/A |
| cg19641455 | NO | NO | NO | N/A | N/A | N/A |
| cg09871669 | NO | YES | NO | N/A | N/A | N/A |
| cg23235154 | NO | YES | NO | N/A | N/A | N/A |
| cg01275297 | NO | NO | YES | YES | YES | NO |
| cg08881796 | NO | YES | NO | N/A | N/A | N/A |
| cg19273773 | NO | YES | NO | N/A | N/A | N/A |
| cg25399461 | NO | NO | NO | N/A | N/A | N/A |
| cg00715047 | NO | YES | NO | N/A | N/A | N/A |
| cg22108374 | NO | YES | NO | N/A | N/A | N/A |
| cg04738827 | NO | YES | NO | N/A | N/A | N/A |
| cg10699064 | NO | YES | NO | N/A | N/A | N/A |
| cg26669159 | NO | YES | NO | N/A | N/A | N/A |
| cg14519125 | NO | YES | NO | N/A | N/A | N/A |
| cg19904265 | NO | YES | NO | N/A | N/A | N/A |
| cg06704773 | NO | YES | NO | N/A | N/A | N/A |
| cg19139210 | NO | YES | NO | N/A | N/A | N/A |
| cg13640100 | NO | YES | NO | N/A | N/A | N/A |
| cg10273210 | NO | YES | NO | N/A | N/A | N/A |
| cg06074958 | NO | YES | NO | N/A | N/A | N/A |
| cg18691434 | NO | NO | NO | N/A | N/A | N/A |
| cg01471153 | NO | NO | YES | YES | YES | NO |
| cg07300558 | NO | YES | NO | N/A | N/A | N/A |
| cg21072025 | NO | YES | NO | N/A | N/A | N/A |
| cg27447053 | NO | YES | NO | N/A | N/A | N/A |
| cg25015277 | NO | YES | NO | N/A | N/A | N/A |
| cg16541852 | NO | YES | NO | N/A | N/A | N/A |
| cg18809289 | NO | YES | NO | N/A | N/A | N/A |
| cg04624363 | NO | YES | NO | N/A | N/A | N/A |
| cg08526825 | NO | YES | NO | N/A | N/A | N/A |
| cg20375220 | NO | YES | NO | N/A | N/A | N/A |
| cg02304580 | NO | YES | NO | N/A | N/A | N/A |
| cg12258785 | NO | YES | NO | N/A | N/A | N/A |
| cg07866909 | NO | YES | NO | N/A | N/A | N/A |
| cg20382695 | NO | YES | NO | N/A | N/A | N/A |
| cg23384185 | NO | YES | NO | N/A | N/A | N/A |
| cg14611683 | NO | YES | NO | N/A | N/A | N/A |
| cg05674150 | NO | YES | NO | N/A | N/A | N/A |
| cg16959606 | NO | YES | NO | N/A | N/A | N/A |
| cg10341152 | NO | YES | NO | N/A | N/A | N/A |
| cg25133192 | NO | YES | NO | N/A | N/A | N/A |
| cg22088594 | NO | YES | NO | N/A | N/A | N/A |
| cg21211187 | NO | YES | NO | N/A | N/A | N/A |
| cg09773586 | NO | YES | NO | N/A | N/A | N/A |
| cg07925311 | NO | NO | YES | YES | YES | NO |
| cg20543544 | NO | YES | NO | N/A | N/A | N/A |
| cg19399220 | NO | YES | NO | N/A | N/A | N/A |
| cg15623892 | NO | YES | NO | N/A | N/A | N/A |
| cg27281285 | NO | YES | NO | N/A | N/A | N/A |
| cg09282289 | NO | YES | NO | N/A | N/A | N/A |
| cg01837410 | NO | YES | NO | N/A | N/A | N/A |
| cg10636745 | NO | NO | YES | YES | YES | YES |
| cg16956501 | NO | YES | NO | N/A | N/A | N/A |
| cg15337815 | NO | YES | NO | N/A | N/A | N/A |
| cg14927724 | NO | YES | NO | N/A | N/A | N/A |
| cg22266824 | NO | YES | NO | N/A | N/A | N/A |
| cg26883434 | NO | YES | NO | N/A | N/A | N/A |
| cg10046620 | NO | YES | NO | N/A | N/A | N/A |
| cg12980128 | NO | YES | NO | N/A | N/A | N/A |
| cg02734527 | NO | YES | NO | N/A | N/A | N/A |
| cg11601932 | NO | YES | NO | N/A | N/A | N/A |
| cg21883293 | NO | YES | NO | N/A | N/A | N/A |
| cg20760116 | NO | YES | NO | N/A | N/A | N/A |
| cg26005232 | NO | YES | NO | N/A | N/A | N/A |
| cg13378934 | NO | NO | NO | N/A | N/A | N/A |
| cg07984256 | NO | YES | NO | N/A | N/A | N/A |
| cg06475223 | YES | YES | YES | YES | YES | NO |
| cg17303833 | NO | YES | NO | N/A | N/A | N/A |
| cg23704082 | NO | YES | NO | N/A | N/A | N/A |
| cg22019158 | NO | YES | NO | N/A | N/A | N/A |
| cg23718606 | NO | YES | NO | N/A | N/A | N/A |
| cg18337963 | NO | YES | NO | N/A | N/A | N/A |
| cg10508778 | NO | NO | NO | N/A | N/A | N/A |
| cg01838971 | NO | YES | NO | N/A | N/A | N/A |
| cg02026306 | NO | YES | NO | N/A | N/A | N/A |
| cg16986298 | NO | YES | NO | N/A | N/A | N/A |
| cg06858294 | NO | YES | NO | N/A | N/A | N/A |
| cg05359249 | NO | YES | NO | N/A | N/A | N/A |
| cg26718511 | NO | YES | NO | N/A | N/A | N/A |
| cg13558810 | NO | YES | NO | N/A | N/A | N/A |
| cg08573435 | NO | YES | NO | N/A | N/A | N/A |
| cg12577850 | NO | NO | NO | N/A | N/A | N/A |
| cg01947224 | NO | YES | NO | N/A | N/A | N/A |
| cg08378505 | NO | YES | NO | N/A | N/A | N/A |
| cg00471645 | NO | NO | NO | N/A | N/A | N/A |
| cg11566154 | NO | YES | NO | N/A | N/A | N/A |
| cg04436083 | NO | YES | NO | N/A | N/A | N/A |
| cg16477091 | NO | YES | NO | N/A | N/A | N/A |
| cg02774015 | NO | YES | NO | N/A | N/A | N/A |
| cg10363337 | NO | NO | NO | N/A | N/A | N/A |
| cg11322797 | NO | YES | NO | N/A | N/A | N/A |
| cg24338780 | NO | NO | NO | N/A | N/A | N/A |
| cg24074033 | NO | YES | NO | N/A | N/A | N/A |
| cg24004532 | NO | YES | NO | N/A | N/A | N/A |
| cg17493727 | NO | YES | NO | N/A | N/A | N/A |
| cg22851880 | NO | YES | NO | N/A | N/A | N/A |
| cg03284308 | NO | YES | NO | N/A | N/A | N/A |
| cg26605700 | NO | YES | NO | N/A | N/A | N/A |
| cg03799530 | NO | YES | NO | N/A | N/A | N/A |
| cg23629722 | NO | NO | NO | N/A | N/A | N/A |
| cg26161329 | NO | YES | NO | N/A | N/A | N/A |
| cg22331349 | NO | YES | NO | N/A | N/A | N/A |
| cg15832311 | NO | YES | NO | N/A | N/A | N/A |
| cg16007541 | NO | YES | NO | N/A | N/A | N/A |
| cg26078793 | NO | YES | NO | N/A | N/A | N/A |
| cg19005368 | NO | YES | NO | N/A | N/A | N/A |
| cg04121261 | NO | NO | NO | N/A | N/A | N/A |
| cg09137382 | NO | YES | NO | N/A | N/A | N/A |
| cg26758551 | NO | YES | NO | N/A | N/A | N/A |
| cg13460409 | NO | YES | NO | N/A | N/A | N/A |
| cg10164300 | NO | NO | NO | N/A | N/A | N/A |
| cg02455820 | NO | NO | NO | N/A | N/A | N/A |
| cg15386368 | NO | NO | NO | N/A | N/A | N/A |
| cg25246431 | NO | YES | NO | N/A | N/A | N/A |
| cg10376827 | NO | YES | NO | N/A | N/A | N/A |
| cg23779890 | NO | YES | NO | N/A | N/A | N/A |
| cg14428310 | NO | YES | NO | N/A | N/A | N/A |
| cg00250500 | NO | YES | NO | N/A | N/A | N/A |
| cg04388983 | NO | YES | NO | N/A | N/A | N/A |
| cg09727692 | NO | NO | NO | N/A | N/A | N/A |
| cg24526702 | NO | NO | NO | N/A | N/A | N/A |
| cg21144922 | NO | NO | NO | N/A | N/A | N/A |
| cg09547119 | NO | YES | NO | N/A | N/A | N/A |
| cg01044580 | NO | YES | NO | N/A | N/A | N/A |
| cg11075316 | NO | YES | NO | N/A | N/A | N/A |
| cg02779535 | NO | YES | NO | N/A | N/A | N/A |
| cg05752664 | NO | YES | NO | N/A | N/A | N/A |
| cg02010772 | NO | YES | NO | N/A | N/A | N/A |
| cg14068796 | NO | YES | NO | N/A | N/A | N/A |
| cg00401091 | NO | YES | NO | N/A | N/A | N/A |
| cg07594674 | NO | YES | NO | N/A | N/A | N/A |
| cg16738971 | NO | YES | NO | N/A | N/A | N/A |
| cg13156863 | NO | YES | NO | N/A | N/A | N/A |
| cg18792022 | NO | NO | NO | N/A | N/A | N/A |
| cg13458335 | NO | YES | NO | N/A | N/A | N/A |
| cg26856080 | NO | YES | NO | N/A | N/A | N/A |
| cg15110463 | NO | NO | NO | N/A | N/A | N/A |
| cg08423149 | NO | YES | NO | N/A | N/A | N/A |
| cg03507326 | NO | NO | NO | N/A | N/A | N/A |
| cg12737588 | NO | NO | NO | N/A | N/A | N/A |
| cg18812909 | NO | YES | NO | N/A | N/A | N/A |
| cg20863756 | NO | YES | NO | N/A | N/A | N/A |
| cg22682373 | NO | YES | NO | N/A | N/A | N/A |
| cg27024922 | NO | YES | NO | N/A | N/A | N/A |
| cg22395019 | NO | YES | NO | N/A | N/A | N/A |
| cg00991848 | NO | YES | NO | N/A | N/A | N/A |
| cg00412842 | NO | YES | NO | N/A | N/A | N/A |
| cg03110382 | NO | YES | NO | N/A | N/A | N/A |
| cg22450406 | NO | YES | NO | N/A | N/A | N/A |
| cg16057598 | NO | YES | NO | N/A | N/A | N/A |
| cg17462962 | NO | YES | NO | N/A | N/A | N/A |
| cg12352399 | NO | YES | NO | N/A | N/A | N/A |
| cg18352162 | NO | YES | NO | N/A | N/A | N/A |
| cg18240400 | NO | YES | NO | N/A | N/A | N/A |
| cg20263901 | NO | YES | NO | N/A | N/A | N/A |
| cg08655662 | NO | YES | NO | N/A | N/A | N/A |
| cg04239375 | NO | YES | NO | N/A | N/A | N/A |
| cg14665951 | NO | YES | NO | N/A | N/A | N/A |
| cg14257676 | NO | NO | NO | N/A | N/A | N/A |
| cg13816999 | NO | YES | NO | N/A | N/A | N/A |
| cg12938003 | NO | NO | NO | N/A | N/A | N/A |
| cg02250400 | NO | YES | NO | N/A | N/A | N/A |
| cg11719157 | NO | YES | NO | N/A | N/A | N/A |
| cg18557556 | NO | YES | NO | N/A | N/A | N/A |
| cg23162310 | NO | YES | NO | N/A | N/A | N/A |
| cg22476550 | NO | YES | NO | N/A | N/A | N/A |
| cg27335600 | NO | YES | NO | N/A | N/A | N/A |
| cg25104186 | NO | YES | NO | N/A | N/A | N/A |
| cg17988780 | NO | YES | NO | N/A | N/A | N/A |
| cg14392031 | NO | NO | NO | N/A | N/A | N/A |
| cg19574915 | NO | YES | NO | N/A | N/A | N/A |
| cg09597312 | NO | YES | NO | N/A | N/A | N/A |
| cg06121469 | NO | YES | NO | N/A | N/A | N/A |
| cg25975256 | NO | YES | NO | N/A | N/A | N/A |
| cg04805619 | NO | YES | NO | N/A | N/A | N/A |
| cg08367318 | NO | YES | NO | N/A | N/A | N/A |
| cg21183502 | NO | YES | NO | N/A | N/A | N/A |
| cg05806054 | NO | YES | NO | N/A | N/A | N/A |
| cg08114812 | NO | NO | NO | N/A | N/A | N/A |
| cg16386080 | NO | YES | NO | N/A | N/A | N/A |
| cg19768599 | NO | YES | NO | N/A | N/A | N/A |
| cg11706635 | NO | YES | NO | N/A | N/A | N/A |
| cg03842205 | NO | YES | NO | N/A | N/A | N/A |
| cg05471495 | NO | YES | NO | N/A | N/A | N/A |
| cg06095695 | NO | NO | NO | N/A | N/A | N/A |
| cg18015985 | NO | YES | NO | N/A | N/A | N/A |
| cg18787012 | NO | YES | NO | N/A | N/A | N/A |
| cg21520772 | NO | YES | NO | N/A | N/A | N/A |
| cg18486906 | NO | YES | NO | N/A | N/A | N/A |
| cg22711679 | NO | NO | NO | N/A | N/A | N/A |
| cg06283270 | NO | YES | NO | N/A | N/A | N/A |
| cg00160619 | NO | YES | NO | N/A | N/A | N/A |
| cg05651778 | NO | YES | NO | N/A | N/A | N/A |
| cg07059052 | NO | YES | NO | N/A | N/A | N/A |
| cg09454892 | NO | YES | NO | N/A | N/A | N/A |
| cg07998461 | NO | YES | NO | N/A | N/A | N/A |
| cg22417733 | NO | YES | NO | N/A | N/A | N/A |
| cg12194745 | NO | YES | NO | N/A | N/A | N/A |
| cg09644065 | NO | NO | NO | N/A | N/A | N/A |
| cg05460965 | NO | NO | NO | N/A | N/A | N/A |
| cg20389635 | NO | YES | NO | N/A | N/A | N/A |
| cg02456288 | NO | YES | NO | N/A | N/A | N/A |
| cg25162948 | NO | YES | NO | N/A | N/A | N/A |
| cg23837438 | NO | YES | NO | N/A | N/A | N/A |
| cg22309696 | NO | YES | NO | N/A | N/A | N/A |
| cg02788146 | NO | YES | NO | N/A | N/A | N/A |
| cg15123562 | NO | YES | NO | N/A | N/A | N/A |
| cg13844474 | NO | YES | NO | N/A | N/A | N/A |
| cg17878972 | NO | YES | NO | N/A | N/A | N/A |
| cg01568784 | NO | YES | NO | N/A | N/A | N/A |
| cg03588039 | NO | YES | NO | N/A | N/A | N/A |
| cg12978575 | NO | NO | NO | N/A | N/A | N/A |
| cg00411411 | NO | YES | NO | N/A | N/A | N/A |
| cg21093807 | NO | YES | NO | N/A | N/A | N/A |
| cg07477282 | NO | YES | NO | N/A | N/A | N/A |
| cg11892747 | NO | NO | NO | N/A | N/A | N/A |
| cg16009734 | NO | NO | NO | N/A | N/A | N/A |
| cg18395636 | NO | YES | NO | N/A | N/A | N/A |
| cg16433737 | NO | NO | NO | N/A | N/A | N/A |
| cg02997982 | NO | NO | NO | N/A | N/A | N/A |
| cg07728507 | NO | YES | NO | N/A | N/A | N/A |
| cg21424782 | NO | YES | NO | N/A | N/A | N/A |
| cg08996748 | NO | YES | NO | N/A | N/A | N/A |
| cg09579953 | NO | YES | NO | N/A | N/A | N/A |
| cg12163823 | NO | YES | NO | N/A | N/A | N/A |
| cg12899421 | NO | YES | NO | N/A | N/A | N/A |
| cg09181644 | NO | NO | NO | N/A | N/A | N/A |
| cg01649611 | NO | YES | NO | N/A | N/A | N/A |
| cg19558029 | NO | YES | NO | N/A | N/A | N/A |
| cg07310916 | NO | YES | NO | N/A | N/A | N/A |
| cg20194973 | NO | NO | NO | N/A | N/A | N/A |
| cg26833936 | NO | YES | NO | N/A | N/A | N/A |
| cg22579950 | NO | YES | NO | N/A | N/A | N/A |
| cg08592707 | NO | NO | NO | N/A | N/A | N/A |
| cg27535538 | NO | NO | NO | N/A | N/A | N/A |
| cg11998703 | NO | YES | NO | N/A | N/A | N/A |
| cg09004287 | NO | NO | NO | N/A | N/A | N/A |
| cg00755063 | NO | YES | NO | N/A | N/A | N/A |
| cg13783238 | NO | YES | NO | N/A | N/A | N/A |
| cg13306815 | NO | YES | NO | N/A | N/A | N/A |
| cg22437153 | NO | NO | NO | N/A | N/A | N/A |
| cg06028917 | NO | YES | NO | N/A | N/A | N/A |
| cg25820257 | NO | YES | NO | N/A | N/A | N/A |
| cg13894021 | NO | YES | NO | N/A | N/A | N/A |
| cg03145963 | NO | NO | NO | N/A | N/A | N/A |
| cg16549027 | NO | YES | NO | N/A | N/A | N/A |
| cg13793048 | NO | YES | NO | N/A | N/A | N/A |
| cg20234855 | NO | YES | NO | N/A | N/A | N/A |
| cg10002569 | NO | NO | NO | N/A | N/A | N/A |
| cg11594887 | NO | NO | NO | N/A | N/A | N/A |
| cg05247391 | NO | YES | NO | N/A | N/A | N/A |
| cg12359904 | NO | YES | NO | N/A | N/A | N/A |
| cg17185710 | NO | YES | NO | N/A | N/A | N/A |
| cg05208878 | NO | YES | NO | N/A | N/A | N/A |
| cg13699808 | NO | YES | NO | N/A | N/A | N/A |
| cg14480507 | NO | YES | NO | N/A | N/A | N/A |
| cg12845952 | NO | YES | NO | N/A | N/A | N/A |
| cg25645310 | NO | YES | NO | N/A | N/A | N/A |
| cg05886574 | NO | NO | NO | N/A | N/A | N/A |
| cg11600807 | NO | YES | NO | N/A | N/A | N/A |
| cg12209165 | NO | YES | NO | N/A | N/A | N/A |
| cg15313320 | NO | YES | NO | N/A | N/A | N/A |
| cg25655593 | NO | YES | NO | N/A | N/A | N/A |
| cg07194321 | NO | YES | NO | N/A | N/A | N/A |
| cg04256466 | NO | NO | NO | N/A | N/A | N/A |
| cg24607283 | NO | YES | NO | N/A | N/A | N/A |
| cg11138362 | NO | NO | NO | N/A | N/A | N/A |
| cg17971578 | NO | NO | NO | N/A | N/A | N/A |
| cg26268968 | NO | YES | NO | N/A | N/A | N/A |
| cg07131451 | NO | YES | NO | N/A | N/A | N/A |
| cg17015340 | NO | YES | NO | N/A | N/A | N/A |
| cg01984858 | NO | NO | NO | N/A | N/A | N/A |
| cg23538901 | NO | YES | NO | N/A | N/A | N/A |
| cg15463803 | NO | YES | NO | N/A | N/A | N/A |
| cg21683284 | NO | YES | NO | N/A | N/A | N/A |
| cg01671895 | NO | NO | NO | N/A | N/A | N/A |
| cg05846166 | NO | YES | NO | N/A | N/A | N/A |
| cg15092561 | NO | NO | NO | N/A | N/A | N/A |
| cg07184013 | NO | NO | NO | N/A | N/A | N/A |
| cg06638966 | NO | YES | NO | N/A | N/A | N/A |
| cg12165758 | NO | YES | NO | N/A | N/A | N/A |
| cg14315058 | NO | NO | NO | N/A | N/A | N/A |
| cg00754688 | NO | NO | NO | N/A | N/A | N/A |
| cg04110283 | NO | NO | NO | N/A | N/A | N/A |
| cg03525385 | NO | YES | NO | N/A | N/A | N/A |
| cg20275507 | NO | NO | NO | N/A | N/A | N/A |
| cg10855746 | NO | NO | NO | N/A | N/A | N/A |
| cg25569840 | NO | YES | NO | N/A | N/A | N/A |
| cg10025830 | NO | YES | NO | N/A | N/A | N/A |
| cg24290286 | NO | YES | NO | N/A | N/A | N/A |
| cg06628000 | NO | YES | NO | N/A | N/A | N/A |
| cg13546858 | NO | YES | NO | N/A | N/A | N/A |
| cg04212239 | NO | YES | NO | N/A | N/A | N/A |
| cg01839993 | NO | YES | NO | N/A | N/A | N/A |
| cg25507001 | NO | YES | NO | N/A | N/A | N/A |
| cg07469815 | NO | YES | NO | N/A | N/A | N/A |
| cg17866732 | NO | YES | NO | N/A | N/A | N/A |
| cg01821557 | NO | NO | NO | N/A | N/A | N/A |
| cg08767938 | NO | YES | NO | N/A | N/A | N/A |
| cg06480265 | NO | YES | NO | N/A | N/A | N/A |
| cg11967546 | NO | YES | NO | N/A | N/A | N/A |
| cg19955284 | NO | YES | NO | N/A | N/A | N/A |
| cg19015951 | NO | YES | NO | N/A | N/A | N/A |
| cg25195288 | NO | YES | NO | N/A | N/A | N/A |
| cg26757711 | NO | YES | NO | N/A | N/A | N/A |
